# Synthesis and anti-hepatocellular carcinoma activity of aminopyridinol–sorafenib hybrids

**DOI:** 10.1080/14756366.2021.1953997

**Published:** 2021-08-02

**Authors:** Bhuwan Prasad Awasthi, Prakash Chaudhary, Diwakar Guragain, Jun-Goo Jee, Jung-Ae Kim, Byeong-Seon Jeong

**Affiliations:** a College of Pharmacy, Yeungnam University, Gyeongsan, Republic of Korea; b College of Pharmacy, Kyungpook National University, Daegu, Republic of Korea

**Keywords:** Molecular hybridisation, Raf kinase, hepatocellular carcinoma, tumour spheroid formation, antitumour activity

## Abstract

Sorafenib is recommended as the primary therapeutic drug for patients with hepatocellular carcinoma. To discover a new compound that avoids low response rates and toxic side effects that occur in sorafenib therapy, we designed and synthesized new hybrid compounds of sorafenib and 2,4,5-trimethylpyridin-3-ols. Compound **6** was selected as the best of 24 hybrids that inhibit each of the four Raf kinases. The anti-proliferative activity of **6** in HepG2, Hep3B, and Huh7 cell lines was slightly lower than that of sorafenib. However, in H6c7 and CCD841 normal epithelial cell lines, the cytotoxicity of **6** was much lower than that of sorafenib. In addition, similar to sorafenib, compound **6** inhibited spheroid forming ability of Hep3B cells *in vitro* and tumour growth in a xenograft tumour model of the chick chorioallantoic membrane implanted with Huh7 cells. Compound **6** may be a promising candidate targeting hepatocellular carcinoma with low toxic side effects on normal cells.

## 1. Introduction

**Figure 1.**
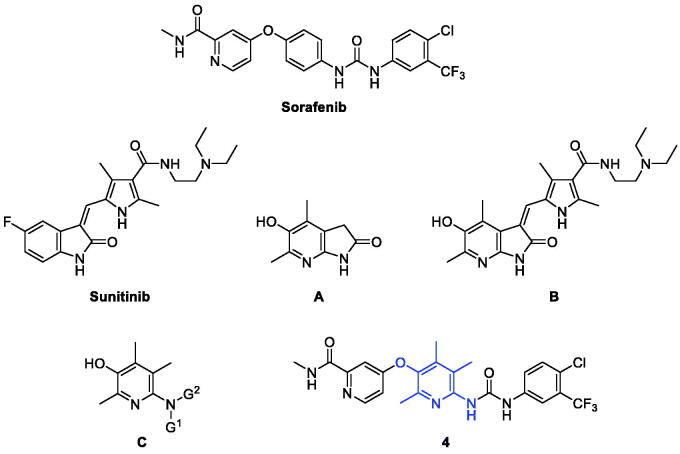
Sorafenib, sunitinib, and their pyridinol hybrids.

Protein kinase is an enzyme catalysing the phosphorylation of a hydroxyl group of a particular protein residue and classified into three broad groups: serine/threonine protein kinases (e.g. Raf, MAPK), tyrosine protein kinases (e.g. VEGFR, PDGFR), and dual-specificity protein kinases (e.g. MEK)^1^. Raf is a serine/threonine kinase, and three different Raf isoforms originating from three independent genes can be distinguished in mammals: A-Raf, B-Raf, and C-Raf (also known as Raf-1)^2^. B-Raf/C-Raf, the predominant heterodimer, activated by growth factor receptor signalling or constitutive Ras activity^3^ play important roles in cell proliferation, differentiation, and survival^4^. Somatic mutations in B-Raf frequently with valine 600 mutation found in some types of cancer cause constitutive activation of the enzyme without heterodimerization^5^ to induce abnormal cell proliferation and survival^6^. The most frequent B-Raf gene mutation is translated into a mutation of valine 600 to glutamic acid (V600E), which has been observed in more than 90% of melanoma^7^. Several inhibitors of B-Raf(V600E) such as vemurafenib and dabrafenib are currently used to treat melanoma in patients with a B-Raf(V600E) mutation^8^^,^^9^. However, patients develop resistance to B-Raf(V600E) inhibitors due to enhanced Raf dimerisation potential by the inhibitors^10^^,^^11^, leading to paradoxical activation of the MAPK signalling pathway. One way to overcome the paradoxical MAPK activation is by developing pan-Raf monomer/dimer inhibitors. The other way to achieve ultimate suppression of cancer growth detouring the Raf dimer dilemma is to block both Raf kinases (B-Raf, C-Raf, and mutant B-Raf) and receptor tyrosine kinases linked to activation of MAPKs, such as VEGFR-2. Sorafenib (Figure 1) is an oral multikinase inhibitor targeting intracellular serine/threonine kinases (C-Raf, B-Raf, and B-Raf(V600E)) and receptor tyrosine kinases (mainly VEGFR-2 and PDGFR-β) which is currently approved for the second-line treatment of advanced renal cell carcinoma (RCC), hepatocellular carcinoma (HCC), acute myeloid leukaemia (AML), and radioactive iodine resistant differentiated advanced thyroid carcinoma (DTC)^12^^,^^13^.

Molecular hybridisation is a structural modification approach that chemically fuses two or more pharmacophores into one compound of a new structure^14^. In most cases, considering new drug design using a molecular hybridisation strategy mainly aims to increase efficacy with broad-spectrum, avoid drug resistance, reduce side effects and toxicity, or improve druggability. Not a few successful cases demonstrated the high feasibility of this strategy and also made molecular hybridisation be recognised as one of the reliable and useful strategies for new drug discovery^15^^,^^16^. We recently reported a study on the design and synthesis of new hybrid compounds (e.g. **B**) that fuses sunitinib with 4,6-dimethyl-5-hydroxy-7-azaindolin-2-one (**A**) (Figure 1)^17^. Sunitinib is an oral multitargeted tyrosine kinase inhibitor and is approved for the treatment of renal cell carcinoma (RCC) and imatinib-resistant gastrointestinal stromal tumour (GIST)^18^. Compound **B** showed *in vitro* cytotoxicity comparable to sunitinib against several cancer cells, and surprisingly, it showed very little toxicity to normal cells. Compared to sunitinib which showed similar levels of cytotoxicity between cancer cells and normal cells, the hybrid compound **B** has much higher safety windows than sunitinib, which can be an advantageous point in the development of a new anticancer drug with lesser toxicity.

As an extension of our efforts for the discovery of new promising compounds by molecular hybridisation, we chose sorafenib as the target compound to fuse with our pyridin-3-ol skeleton as in the structure of **C** for this study. Pyridoxine-derived 2,4,5-trimethylpyridin-3-ol derivatives with various nitrogen-containing functional groups at *para*-position to the hydroxyl group have been designed and developed by us in the last decade^19–33^. This series of compounds **C** showed good pharmacological actions against reactive oxygen species, angiogenesis, inflammatory bowel disease, autoimmune disease, and cancer. In the structure of new hybrid compounds **4**, the central aminophenol substructure in sorafenib is replaced by the 2,4,5-trimethylpyridin-3-ol moiety in compound **C**.

## 2. Results and discussion

### 2.1. Synthesis of the sorafenib–aminopyridinol hybrid compound 4

**Scheme 1.**
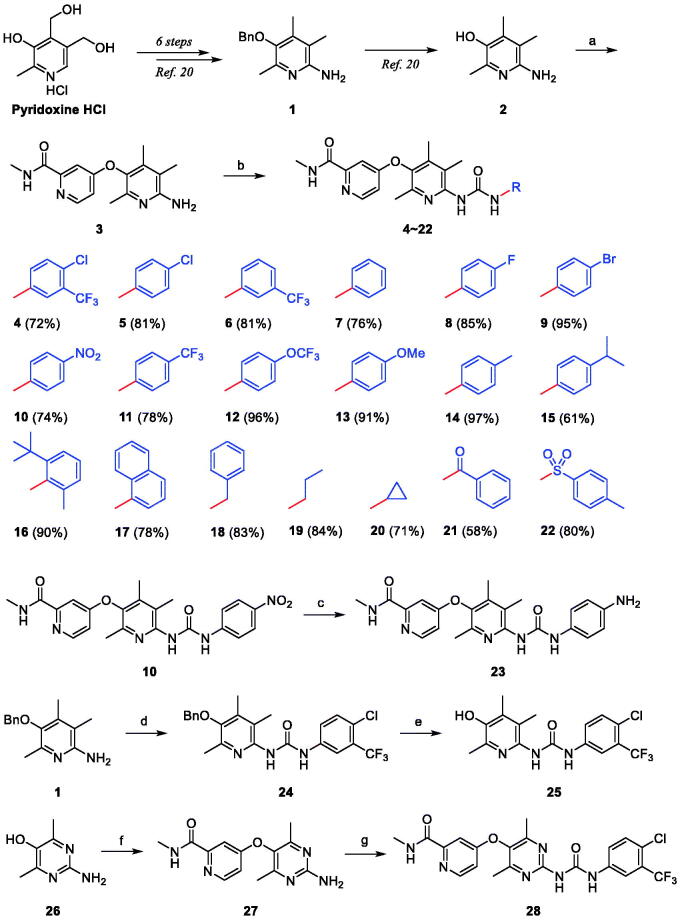
Synthesis of pyridinol-sorafenib hybrid compounds. *Reagents and conditions*: (a) KO*^t^*Bu, DMF, μW, 200 °C, 4.5 h, 87%; (b) R-N = C = O, DCM, r.t.; (c) SnCl_2_, EtOH-CHCl_3_ (1:1), 80 °C, 12 h, 62%; (d) 4-chloro-3-(trifluoromethyl)phenyl isocyanate, DCM, r.t., 2 h, 81%; (e) BCl_3_, pentamethylbenzene, DCM, 0 °C, 30 min, 87%; (f) 4-chloro-*N*-methylpicolinamide, KO*^t^*Bu, DMF, μW, 200 °C, 4.5 h, 80%; (g) 4-chloro-3-(trifluoromethyl)phenyl isocyanate, DCM, r.t., 18 h, 58%.

A key feature of the synthesis of compound **4**, a hybrid structure of sorafenib and aminopyridinol, is to combine the moieties at both ends of sorafenib, i.e. 4-chloro-*N*-methylpicolinamide and 4-chloro-3-(trifluoromethyl)phenyl isocyanate, with centrally located 6-amino-2,4,5-trimethylpyridin-3-ol (**2**) (Scheme 1). The formation of the ethereal bond of **3** by nucleophilic aromatic substitution reaction between the oxygen atom of the hydroxyl group in **2** and the carbon atom with chloride in 4-chloro-*N*-methylpicolinamide was carried out under microwave irradiation with potassium *tert*-butoxide base in a sealed tube. Then, the other substructure of sorafenib was linked to the aminopyridinol centre by nucleophilic addition reaction of the nitrogen atom in the amino group of **3** to the carbon atom in the isocyanate group in 4-chloro-3-(trifluoromethyl)phenyl isocyanate to form ureido compound **4**. The key intermediate **2** was readily prepared by the well-established method which was developed by us starting from pyridoxine hydrochloride^20^.

### 2.2. Comparison of inhibitory activity of the two compounds, sorafenib and 4, against four Raf kinases

**Figure 2.**
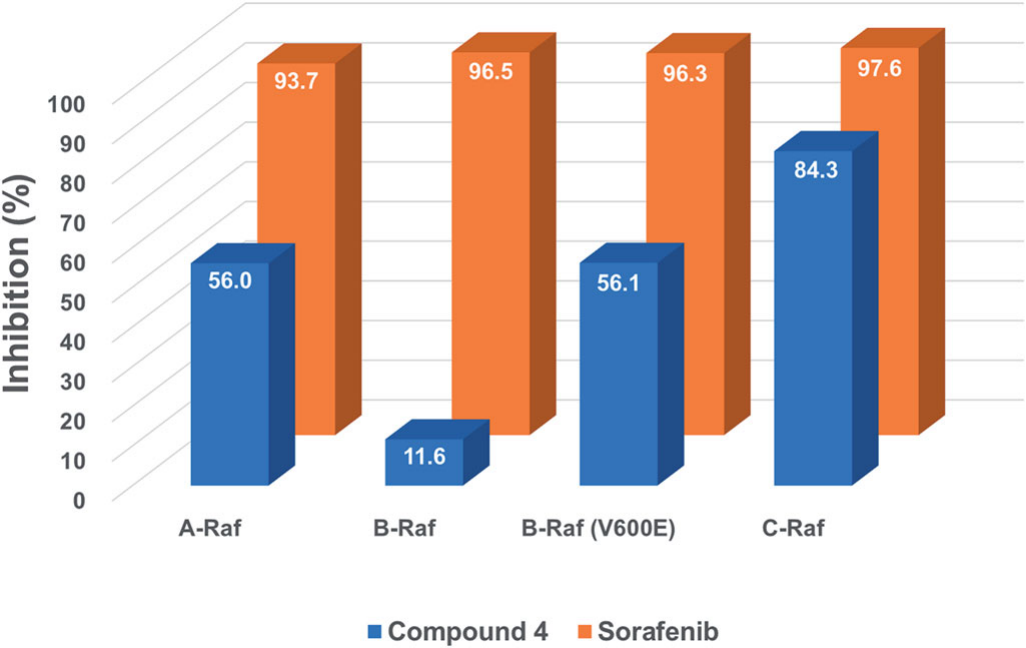
Inhibitory activities of compound **4** and sorafenib at a fixed concentration (1 μM) against four Raf kinases. The numbers in the bar-graphs are mean values obtained from two independent experiments performed by the Reaction Biology Corp. (Malvern, PA).

Inhibitory effects of the new hybrid compound **4** on four key target kinases of sorafenib, namely A-Raf, B-Raf, B-Raf(V600E), and C-Raf, were measured by cell-free kinase assay systems at a fixed concentration (1 μM). As shown in Figure 2, the assay results, against our expectations, showed general low inhibitory activities of compound **4** compared to sorafenib. The inhibitory activity of compound **4** against C-Raf showed a bit less than that of sorafenib, but it was far behind in the cases of the other three Rafs. In particular, the inhibition of B-Raf with compound **4** was seriously low. These somewhat disappointing results have strongly driven the need for the design and synthesis of derivatives with more diverse structures.

### 2.3. Structural modification of compound 4

For structural modification of compound **4**, we first noted a paper published in 2002 by the Bayer Corporation, the original developer of sorafenib^34^. This paper summarises the history of design and discovery from the hit compound found through high-throughput screening targeting C-Raf kinase to the final sorafenib using the medicinal chemistry approach. Considering the three key facts which are (i) the existence of carboxamide functionality in the pyridine ring was found to be the essential factor for potency, (ii) the primary amides showed better activity than the secondary amides due to providing a hydrogen-bond doner in this region, and (iii) the *N*-methyl substituent was the best compared to other bulkier ones, we decided to design new derivatives that mainly modified the structure of the urea-side part without touching the *N*-methylpicolinamide part. Many of the new derivatives designed have *N*-phenyl group with various substituents as in compounds **5**–**17**, and **23**. Included were compounds with *N*-benzyl **18**, *N*-alkyl **19** and **20**, *N-*benzoyl **21**, and *N*-tosyl **22** in the list of new derivatives as well. Compound **24** where *N*-methylpicolinamide moiety of compound **4** is replaced by benzyl group was picked for biological evaluation to see the effect of that moiety on potency. Moreover, the free OH analogue **25** in which *N*-methylpicolinamide was entirely removed was also included in the compound list. Lastly, we wanted to examine how the dimethylpyrimidine ring in **28** instead of the trimethylpyridine ring in **4** affects inhibitory activity. The 18 derivatives **5**–**17** were readily prepared by the coupling reaction between the common substrate **3** and the commercially available corresponding isocyanates^28^. The aminophenyl derivative **23** was obtained from the corresponding nitrophenyl compound **10** by reduction with tin(II) chloride. The benzyloxy analogue **24** was transformed to free hydroxy compound **25** by boron trichloride along with pentamethylbenzene as a non-Lewis basic cation scavenger. For the synthesis of the pyrimidine-centred analogue **28**, 2-amino-4,6-dimethylpyrimidin-5-ol (**26**) was needed and prepared by slightly modifying the known methods^35^^,^^36^. The synthetic strategy used to prepare compound **4** was also applied here to synthesise compound **28** finally.

### 2.4. Raf inhibitory abilities of new compounds

**Figure 3.**
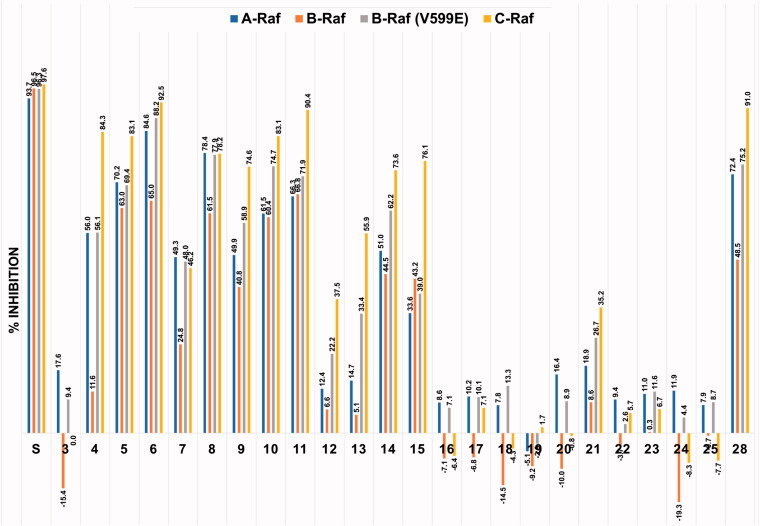
Inhibitory activities of the twenty-three derivatives of compound **4** along with sorafenib (**S**) at a fixed concentration (1 μM) against four Raf kinases. The numbers in the bar-graphs are mean values of % inhibition obtained from two independent experiments performed by the Reaction Biology Corp. (Malvern, PA).

The new derivatives were subjected to cell-free kinase assay to examine their inhibitory activities against the four Raf kinases at a fixed concentration of 1 μM. The assay results are shown in Figure 3 and the mean value of % inhibition for each compound was obtained from two independent experiments.

As shown in Figure 3, sorafenib (**S**) was observed to have similar potent inhibitory effects on all four Raf kinases. On the other hand, looking at the sorafenib–trimethylpyridinol hybrid compounds with inhibitory activity, the degree of inhibition against B-Raf was particularly low compared to the inhibition tendency against other Rafs, and the inhibition against C-Raf showed the highest. This trend was most noticeably observed in compound **4**, which had the structure closest to sorafenib. Its level of c-Raf inhibition was 84%, which contrasted sharply with 12% inhibition against B-Raf. The inhibitory effect of compound **3**, which was used as the key intermediate in the preparation of many derivatives, was also measured. This was to confirm the correlation between the presence of the urea-side moiety and the Rafs inhibitory activity. In the absence of the ureido-part, it was observed that the inhibitory effect almost disappeared. As in compounds **5** and **6**, the inhibitory activity was significantly restored if only one of the two substituents, chloro and trifluoromethyl, remained in the urea-side moiety of sorafenib structure. However, as in compound **7**, when both substituents of the phenyl group were removed, the inhibitory ability considerably deteriorated. In the series of derivatives in which various substituents were introduced at the *para*-position of the urea-side phenyl ring, it was confirmed that the inhibitory activity tends to vary depending on the type of the substituent. First, as in compounds **8**–**11**, it was observed that the inhibitory activity was maintained to some extent by the substituents, F, Br, NO_2_, and CF_3_, that withdraw electrons. Second, in the case of substituents in which oxygen is directly connected to the phenyl ring, such as trifluoromethoxy **12** and methoxy **13**, the inhibitory activity was remarkably reduced, whereas in the cases of alkyl group such as methyl **14** and isopropyl **15** there was no significant decrease in inhibitory activity. Lastly, in the case of compound **23** in which an amino group was introduced, the inhibitory activity almost disappeared. As in compounds **16** and **17**, when steric bulkiness occurred at the *ortho*-position of the urea in the phenyl ring, the inhibitory ability dropped sharply. The benzyl compound **18**, which has a methylene linker between the ureido nitrogen and the phenyl ring, was much less effective than compound **7**, in which ureido nitrogen is directly connected to phenyl ring. Compound **19**, which has no phenyl group and *n*-propyl group directly connected to ureido nitrogen had almost zero inhibitory activity, and the cyclopropyl-containing compound **20** showed slightly better results than n-propyl one, but still had little activity. Compounds **21** and **22**, each containing a carbonyl group and a sulphonyl group between urea and phenyl ring, respectively, showed weak or inhibitory activity. The importance of the existence of the *N*-methylpicolinamide moiety was briefly investigated. Both compound **24**, which replaced it with benzyl moiety, and compound **25**, which completely removed it, caused a serious loss of inhibitory activity. in compound **4**, when the trimethylpyridine core ring was changed to dimethylpyrimidine ring as in compound **28**, the inhibitory activity was slightly improved.

Compounds **4** and **6** were further examined for half maximal inhibitory concentration (IC_50_) against four different Raf kinases and the results are summarised in Table 1. The IC_50_ values showed a tendency to be roughly similar to the percent inhibition values measured at the 1 μM concentration. The IC_50_ values of compound **4** were 6.9–90 times higher than those of sorafenib, whereas compound **6** showed IC_50_ values of 1.8–3.3 times higher than that of sorafenib. The difference in the inhibitory activity against each of the four Raf kinases was the same as sorafenib which showed the weakest inhibitory activity against B-Raf.

**Table 1.** IC_50_ values of the selected compounds against Raf kinases.

Compound No.	*In vitro* Raf kinase inhibition IC_50_ (nM)^a^			
	A-Raf	B-Raf	B-Raf(V600E)	C-Raf
Sorafenib	19 ± 3.3	65 ± 2.5	24 ± 1.7	12 ± 1.4
**4**	312 ± 25.9	5,860 ± 360.6	650 ± 2.3	80 ± 8.1
**6**	57 ± 8.6	216 ± 43.6	70 ± 0.4	22 ± 0.8

^a^The values are mean ± SD of two independent experiments performed by the Reaction Biology Corp. (Malvern, PA).

### 2.5. Molecular docking

**Figure 4.**
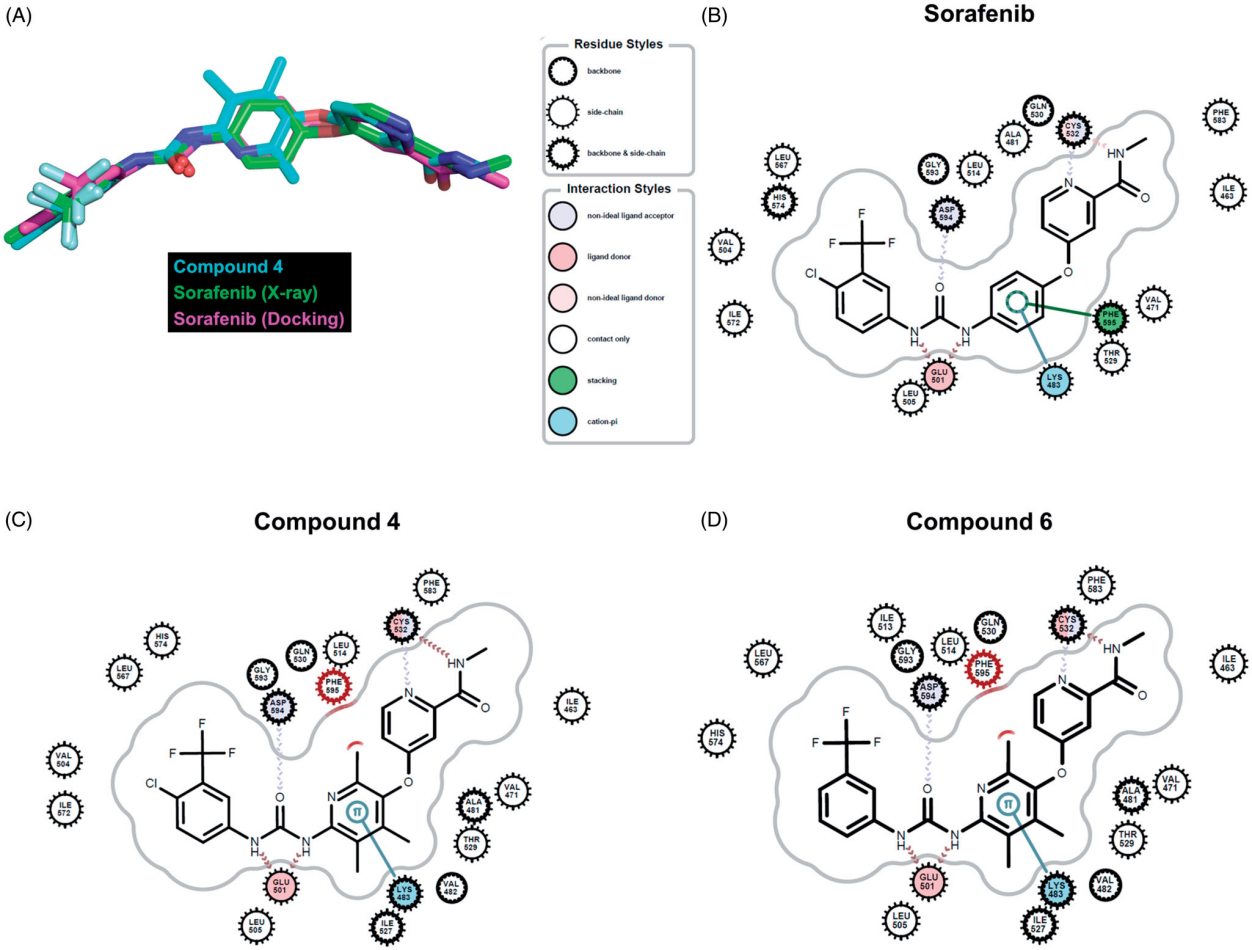
Comparison of docking poses. (A) Overlaid conformers of sorafenib (purple) in the crystal structure (Chain A of 1UWJ), sorafenib (green) and compound **4** (cyan) in the docking. (B)∼(D) Intermolecular interactions between sorafenib (B), compound **4** (C), compound **6** (D), and B-Raf (1UWJ) were visualised in the docked poses prepared by Glide-SP^36^. The annotations follow the definition of the Grapheme Toolkit of Openeye package (Santa Fe, NM).

Eight kinase structures containing sorafenib have been known. B-Raf, CDK8, KDR, and P38 belong to the kinases. When we align the complex structures with an identical direction, all sorafenib conformations are almost invariant, suggesting the critical intermolecular interactions are shared. To understand the difference of the inhibitory activities in the synthesised molecules at the atomic level, we performed docking studies using sorafenib, compound **4**, and compound **6**. To improve two key factors in the docking, poses and energies, we undertook a series of tasks. First, we selected the template coordinate that reproduces the crystal poses of the ligands the most. The RCSB protein data bank includes 45 inhibitors complexed with B-Raf proteins. We docked the inhibitors into the RCSB-deposited 86 B-Raf coordinates using Glide-SP^37^ and counted the poses reproduced within less than 2 Å rmsd (root mean square deviation in the heavy atoms) compared to the crystal ligands. The coordinate of 1UWJ-A faithfully reproduced the poses the most in 22 cases with reasonable geometries (Figure S1 in Supplemental material). Interestingly, the co-crystalized ligand with 1UWJ-A is sorafenib (BAY43-9006). The value of rmsd between crystal and docked sorafenib was 0.374 Å. We, second, generated the physicochemically matched but topologically different 50 decoys per inhibitor from the 45 crystal inhibitors using the DUD-E server^38^ and handled them as false positives. The docking with 1UWJ-A and the mixture of true positives and false negatives showed early enrichment of true positives over false positives in the scores. The values of AUC and LogAUC in the docked results were 0.84 and 0.42 for the case of 1UWJ-A, clearly indicating the early enrichments (Figure S2 in Supplemental material). Note that the higher values of AUC and LogAUC can reflect the faithfulness of the docking^39^. Third, we performed MMPBSA refinements using three different 20 ns molecular dynamics simulations per inhibitor to approximate the free energies upon the binding^40^. Sorafenib, compound **4**, and compound **6** showed the values of −15.0 (± 7.0), −9.3 (± 6.7), and −17.3 (± 1.2) kcal/mol, respectively (Figure S3 in Supplemental material). The results qualitatively agree with the observation that the inhibitory activities of compound **4** deviated from those of sorafenib and compound **6**. Then can the docked poses explain the difference in the inhibitions? Three molecules shared the apparently similar poses. Small but noticeable changes between sorafenib and the other two were the phenoxy moiety of sorafenib deviated slightly from compounds **4** and **6** (Figure 4(A)). The trimethyl groups of compounds **4** and **6** seemingly cause the difference. The moiety participates in two intermolecular interactions with Lys-483 and Phe-595 of B-Raf. Lys-483 forms the cation-π contact, whereas Phe-595 is involved in the aromatic–aromatic interaction. The subtle change of the phenoxy moiety in compound **4**, and compound **6** resulted in the loss of the aromatic–aromatic interaction through Phe-595 (Figure 4(C,D)). It may explain the decreased inhibition of compounds **4** and **6** from sorafenib, though the difference between sorafenib and compound **6** was not distinguishable in MMPBSA.

### 2.6. Inhibitory activities of compounds 4 and 6 against VEGFR-2 tyrosine kinase

The anticancer effect of sorafenib is also associated with inhibition of angiogenesis in HCC which is a highly vascularised tumour, and the antiangiogenic effect of sorafenib is mediated through blocking VEGFR-2 and other receptor tyrosine kinases. We also compared the inhibitory activity of the hybrid compounds **4** and **6** against VEGFR-2 tyrosine kinase with sorafenib. At a fixed concentration (1 µM), sorafenib showed about 95% inhibitory activity against VEGFR-2 tyrosine kinase, whereas inhibitory activities of compounds **4** and **6** were about 32% and −6%, respectively.

### 2.7. Comparison of antiproliferative and cytotoxic activities of the hybrid compounds and sorafenib

Anti-proliferative activities of compounds **4** and **6** were examined by using three representative HCC cell lines, HepG2, Hep3B, and Huh7, in which Ras/Raf/MAPK signalling pathway is abnormally activated^41^ in along with overexpression of EGFR, IGFR, VEGFR, and c-Met^42^. In the three HCC cell lines, the growth inhibitory effects of compounds **4** and **6** were slightly lower than that of sorafenib (Table 2).

**Table 2.** IC_50_ values of the selected compounds against three human liver cancer cell lines.

Compound No.	Cancer cell proliferation IC_50_ (μM)^a^		
	HepG2	Hep3B	Huh7
Sorafenib	1.29 ± 0.02*	1.22 ± 0.05*	1.17 ± 0.04*
**4**	6.98 ± 0.08*	6.23 ± 0.07*	5.29 ± 0.11*
**6**	5.02 ± 0.06*	6.49 ± 0.12*	4.77 ± 0.09*

^a^The values are mean ± SEM of three independent experiments performed in triplicate.

*p < 0.05 versus the vehicle-treated control group.

In patients receiving sorafenib, adverse events have been reported, and the events are predominantly diarrhoea, weight loss, and hand-foot skin reactions^43^. Since the side effects of the orally available anticancer drug sorafenib seem to be closely related to epithelial cell injury in the gastrointestinal tract and skin, the cytotoxic effects of the hybrid compounds and sorafenib in normal epithelial cells were compared. Cytotoxic activities of compounds **4** and **6** turned out to be much lower than that of sorafenib. In H6c7, a normal pancreatic ductal epithelial cell line, and CCD841, a normal colon epithelial cell line, the half maximal cytotoxic concentrations (CC_50_) of sorafenib were about 4.4 µM and 4.1 µM, respectively, whereas CC_50_ of compounds **4** and **6** were over 10 µM in both H6c7 and CCD841 cell lines (Table 3).

**Table 3.** CC_50_ of the selected compounds against two human normal cell lines.

Compound No.	Normal cell cytotoxicity CC_50_ (μM)^a^	
	H6c7	CCD841
Sorafenib	4.36 ± 0.21	4.08 ± 0.58
**4**	>10	>10
**6**	>10	>10

^a^The values are mean ± SEM of three independent experiments performed in triplicate.

### 2.8. Effects of hybrid compounds and sorafenib on sphere forming ability of HCC cell lines

**Figure 5.**
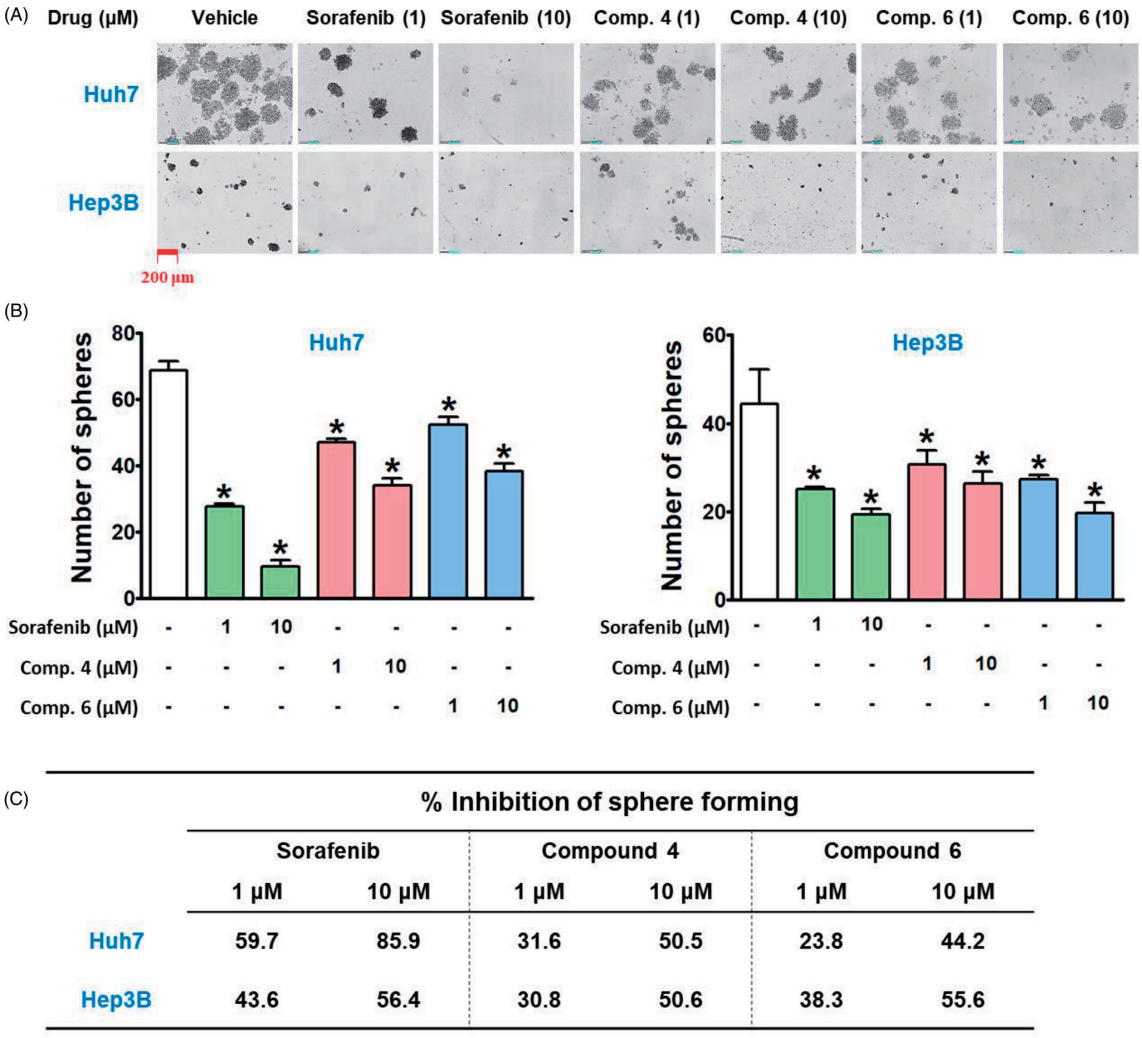
Inhibitory effects of hybrid compounds and sorafenib on sphere forming ability of Huh7 and Hep3B cells. (A) Spheroid cultures of Huh7 and Hep3B cells. After cells were treated with drugs for 48 h, the cells were trypsinised and seeded with equal number of cells in each well of ultra-low attachment 24-well plate in stem cell permissive medium for 13 days. The red bar in the image indicates 200 μm. (B) The number of spheroids over 50 μm in diameter on the captured images by Celloger Mini (4× objective magnification) was counted by using Image J software. *p < 0.05 compared to the vehicle-treated group.

Cancer stem cells (CSCs), a subset within tumour tissues, possess the ability to self-renewal and change their properties depending on their surrounding environments, resulting in resistance to conventional therapies, recurrent growth, and distant metastasis^44^^,^^45^. We compared the effects of hybrid compounds and sorafenib on the maintenance of CSC population of HCC cell lines by using a sphere-forming assay. In the anchorage-independent sphere-forming assay with serum-free, non-adherent, and nutrition-deficient culture conditions, differentiated tumour cells undergo apoptosis, while CSCs survive, adapt, and proliferate, resulting in the enrichment of the potential CSC subpopulation^46^^,^^47^. Because the presence of hepatitis B virus X protein (HBx) is responsible for resistance to targeted therapies in HCC^48^, Hep3B (HBx-positive) and Huh7 (HBx-negative) cells were selected to investigate their spheroid forming ability and reactivity to the hybrid compounds and sorafenib. Huh7 cells formed more and bigger spheroids than Hep3B cells (Figure 5(A)), and a spheroid-suppressing effect of sorafenib was stronger in Huh7 than in Hep3B cells. However, the inhibitory effect of compound **4** on spheroid formation was similar in both cell lines, whereas compound **6** showed a better inhibitory effect on Hep3B than on Huh7 spheroid formation (Figure 5(B)).

### 2.9. Anti-tumour effects of hybrid compounds on a CAM tumour model implanted with Huh7 cells

**Figure 6.**
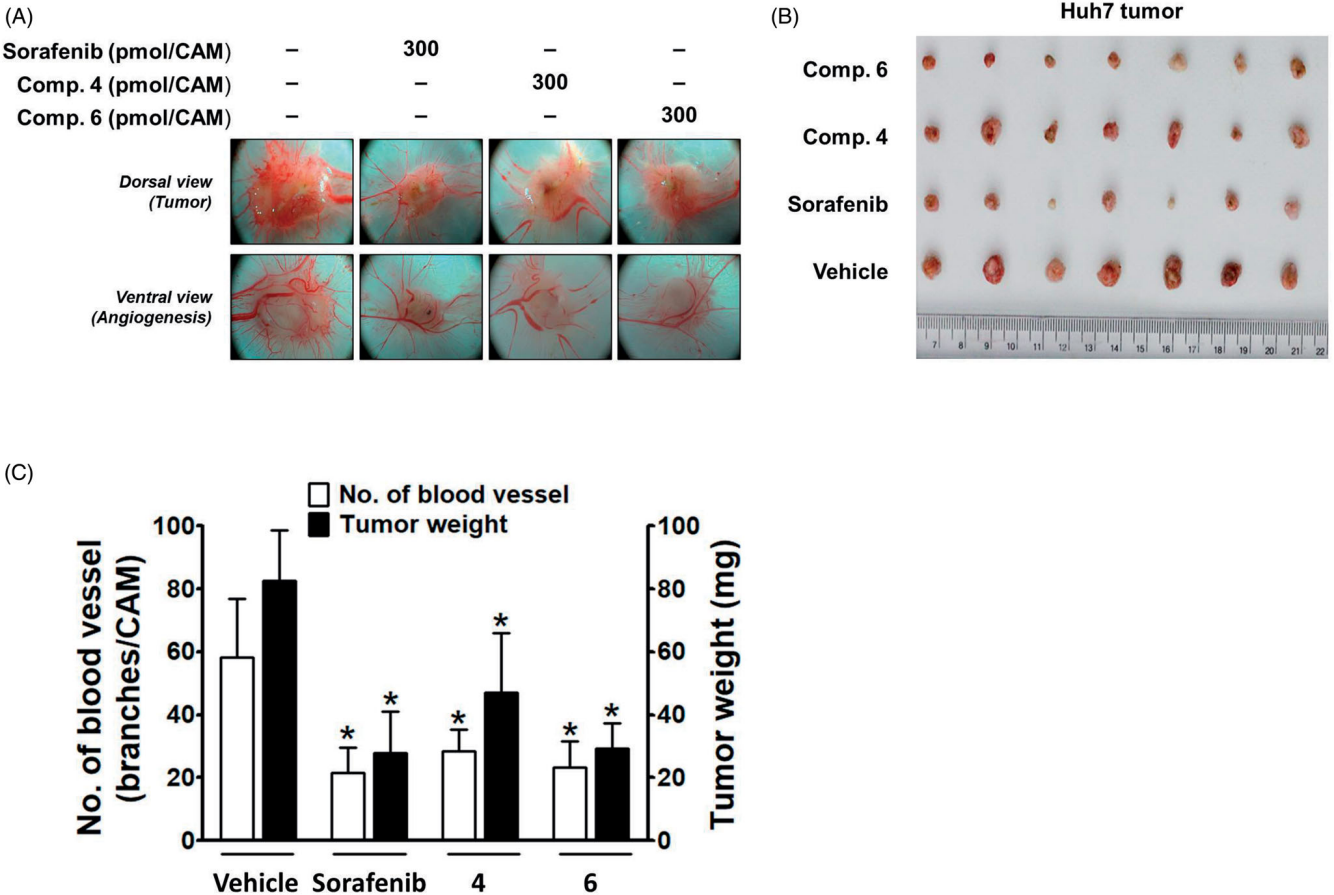
Inhibitory effects of compounds **4** and **6** along with sorafenib on tumour growth and tumour-induced angiogenesis in Huh7-xenografted CAM tumour model. Five days after Huh7 xenograft, both tumour growth and tumour-induced angiogenesis on the CAM tissues are shown in (A). The weight of tumour masses isolated from CAMs (B) and the number of new vessel branches formed on CAM were quantitated and counted using Image J program (C). *p < 0.05 compared to the vehicle-treated group.

Next, the *in vivo* efficacy of compounds **4** and **6** on the growth of Huh7 cells xenografted onto CAM was compared with that of sorafenib. In the CAM tumour model, implanted Huh7 cells developed a tumour mass and tumour-induced angiogenesis (Figure 6(A)). The inhibitory effect of compound **4** on tumour weight was weaker than that of sorafenib, whereas compound **6** showed an inhibitory effect similar to sorafenib (Figure 6(B,C)). The tumour-induced angiogenesis was also significantly blocked by sorafenib, and compounds **4** and **6** showed a similar degree of inhibition as sorafenib (Figure 6(C)).

## 3. Conclusion

We designed and synthesised a series of novel compounds by molecular hybridisation of 6-amino-2,4,5-trimethylpyridin-3-ol and sorafenib. From the *in vitro* kinase assay using four different Raf kinases, compound **6** was found to have similar inhibitory activity to sorafenib. The detailed analysis using docking simulation may provide a clue for the quantitative understanding of B-Raf and inhibitors' interaction. The *in vitro* inhibitory activity of the hybrid compounds **4** and **6** on the proliferation of HCC cell lines were correlated with the inhibitory activity against Raf kinases, and the degree of inhibitory activity was strong in the order of sorafenib, **6**, and **4**. HCC cell lines had different spheroid-forming abilities, with Huh7 forming larger spheroid faster. Sorafenib showed stronger inhibitory activity on the spheroid forming ability of Huh7 than Hep3B, whereas compound **6** more strongly inhibited the spheroid formation of Hep3B. The results suggest that compound **6** has good inhibitory activity against CSC formation of HBx-associated HCC. In conjunction with the previous report that HBx is responsible for resistance to targeted therapies in HCC^49^, the stronger action of compound **6** against Hep3B than Huh7 also suggest that compound **6** may be a better choice than sorafenib for the treatment of a type of HCC, HBx-positive, which develops resistance easily. In conclusion, although inhibitory activities of compound **6** against Raf kinases and VEGFR2 were weaker than those of sorafenib, the similar degree of *in vivo* anti-tumour efficacy suggests that compound **6** may be considered as an effective compound targeting HBx-positive HCC while causing a less toxic effect on normal epithelial cells. Our current study did not dissect the difference in the inhibitory activities of the small molecules for B-Raf, B-Raf(V600E), and C-Raf. To know how the change of polypharmacological profiles at the enzyme level leads to the different phenotypes in cell and animal models will be an interesting and challenging topic. Our data may be a meaningful addition to this direction.

## 4. Materials and methods

### 4.1. Synthesis

#### 4.1.1. General

Unless noted otherwise, materials were purchased from commercial suppliers and used without further purification. Air or moisture-sensitive reactions were carried out under an inert gas atmosphere. The reaction progress was monitored by thin layer-chromatography (TLC) using silica gel F_254_ plates. The products were purified by flash column chromatography using silica gel 60 (70–230 mesh) or by using the Biotage “Isolera One” system with indicated solvents. Melting points were determined using a Fisher–Johns melting point apparatus and were not corrected. Low-resolution mass spectra (LRMS) were obtained using a Jeol ColdSpray-LC-TOF-MS and recorded in a positive ion mode with an electrospray (ESI) source. NMR spectra were obtained using a Bruker-250 spectrometer (250 MHz for ^1^H-NMR, and 62.5 MHz for ^13^C-NMR) and a Bruker Avance Neo 400 spectrometer (400 MHz for ^1^H-NMR, and 100 MHz for ^13^C-NMR). Chemical shifts (*δ*) were expressed in ppm using a solvent as an internal standard and the coupling constant (J) in Hertz. HPLC analyses were performed on a system consisted of an LC-20AD pump, a CBM-20A communication bus module, an SPD-20A UV–visible detector, and a DGU-20A5 degasser from Shimadzu Corporation (Kyoto, Japan). A Phenomenex Luna^®^ C18 column (250 × 4.6 mm, 5.0 µm) was used with a gradient solvent system consisted of acetonitrile and water (from 10% to 100% of acetonitrile over 15 min, then 100% of acetonitrile for 10 min) at a flow rate of 1.0 ml/min at 254 nm UV detection. The purity of compound was recorded as a percentage (%) and the retention time was given in minutes.

#### 4.1.2. 4-((6-Amino-2,4,5-trimethylpyridin-3-yl)oxy)-N-methylpicolinamide (3)

To a solution of 6-amino-2,4,5-trimethylpyridin-3-ol (**2**, 440 mg, 2.89 mmol) in *N*,*N*-dimethylformamide (15 ml) was added 1 M potassium *tert*-butoxide in *tert*-butanol (4.6 ml). After stirring for 30 min, 4-chloro-*N*-methyl-2-pyridinecarboxamide (543 mg, 3.18 mmol) was added and the mixture was microwaved with power 200 W for 4.5 h at 200 °C. The reaction mixture was diluted with ethyl acetate and water, and the aqueous layer was extracted with ethyl acetate. Organic layer was dried over anhydrous magnesium sulphate and concentrated. The residue was washed several times with diethyl ether to get **3** (716 mg, 87%). Beige solid; *R*_f_. 0.20 (CH_2_Cl_2_:MeOH = 20:1); m.p. 193 °C; MS (ESI) *m/z* 287.2 [M + H]^+^; ^1^H-NMR (DMSO-d_6_) *δ* 8.74 (d, *J* = 4.8 Hz, 1H), 8.46 (d, *J* = 5.6 Hz, 1H), 7.23 (d, *J* = 2.5 Hz, 1H), 7.03 (dd, *J* = 5.6, 2.6 Hz, 1H), 5.65 (s, 2H), 2.77 (d, *J* = 4.8 Hz, 3H), 1.98 (d, *J* = 2.1 Hz, 6H), 1.89 (s, 3H); ^13 ^C-NMR (DMSO-d_6_) *δ* 165.5, 163.8, 155.5, 152.6, 150.5, 144.4, 138.0, 137.4, 113.1, 112.9, 107.5, 26.0, 18.4, 12.9, 12.3; HPLC retention time 11.0 min, purity 97.8%.

#### 4.1.3. General procedure for the synthesis of compounds 4–22

To a solution of **3** (150 mg, 0.52 mmol) in dichloromethane (4 ml) was added an isocyanate (0.83 mmol). The mixture was stirred at room temperature. After concentration of the reaction mixture, the residue was purified by silica gel column chromatography to give compounds **4**–**22**.

#### 4.1.4. 4-((6–(3-(4-Chloro-3-(trifluoromethyl)phenyl)ureido)-2,4,5-trimethylpyridin-3-yl)oxy)-N-methylpicolinamide (4)

Reaction time 6 h; 72% yield; White solid; *R*_f_. 0.31 (CH_2_Cl_2_:MeOH = 20:1); m.p. 265 °C; MS (ESI) *m/z* 530.0 [M + H]^+^; ^1^H-NMR (CDCl_3_) *δ* 12.53 (s, 1H), 8.42 (d, *J* = 5.6 Hz, 1H), 8.00 (d, *J* = 5.1 Hz, 1H), 7.93 (d, *J* = 2.5 Hz, 1H), 7.75 (dd, *J* = 8.7, 2.6 Hz, 1H), 7.50 (d, *J* = 2.4 Hz, 1H), 7.45 (d, *J* = 8.8 Hz, 1H), 7.06 (s, 1H), 6.93 (dd, *J* = 5.6, 2.6 Hz, 1H), 3.01 (d, *J* = 5.1 Hz, 3H), 2.33 (s, 3H), 2.23 (s, 3H), 2.11 (s, 3H); HPLC retention time 20.0 min, purity 99.6%. Ditosylate salt **4–1** of compound **4** was prepared for further analyses.

#### 4.1.5. 4-((6–(3-(4-Chloro-3-(trifluoromethyl)phenyl)ureido)-2,4,5-trimethylpyridin-3-yl)oxy)-N-methylpicolinamide ditosylate (4–1)

To a suspension of **4** (20 mg, 0.039 mmol) in ethanol (2 ml) was added *p*-toluenesulphonic acid (15.0 mg, 0.078 mmol). The mixture was stirred at 60 °C for 1 h. The resulting solution was concentrated to give **4–1** (100%). White solid; *R*_f_. 0.31 (CH_2_Cl_2_:MeOH = 20:1); ^1^H-NMR (CD_3_OD) *δ* 8.79 (d, *J* = 6.6 Hz, 1H), 8.05 (dd, *J* = 13.3, 2.4 Hz, 2H), 7.80 (dd, *J* = 6.5, 2.5 Hz, 1H), 7.65 (d, *J* = 8.1 Hz, 6H), 7.56 (d, *J* = 8.8 Hz, 1H), 7.19 (d, *J* = 8.0 Hz, 5H), 2.88 (s, 3H), 2.53 (s, 3H), 2.37 (s, 3H), 2.32 (s, 9H); ^13^C-NMR (CD_3_OD) *δ* 170.8, 160.4, 154.6, 152.7, 148.4, 147.4, 147.0, 143.3, 142.8, 141.9 (2 C), 140.8, 138.1, 133.3, 129.9 (4 C), 129.3, 127.5, 126.9 (4 C), 126.3, 125.6, 125.2, 119.6 (d, *J* = 5.6 Hz), 116.8, 111.9, 27.2, 21.3 (2 C), 15.5, 14.8, 13.3.

#### 4.1.6. 4-((6–(3-(4-Chlorophenyl)ureido)-2,4,5-trimethylpyridin-3-yl)oxy)-N-methylpicolinamide (5)

Reaction time 8 h; 81% yield; White solid; *R*_f_. 0.32 (CH_2_Cl_2_:MeOH = 20:1); m.p. 248 °C; MS (ESI) *m/z* 462.1 [M + Na]^+^; ^1^H-NMR (CDCl_3_) *δ* 12.28 (s, 1H), 8.41 (d, *J* = 5.6 Hz, 1H), 8.01 (dd, *J* = 9.2, 4.2 Hz, 1H), 7.54 (d, *J* = 8.7 Hz, 3H), 7.29 (d, *J* = 8.9 Hz, 2H), 6.91 (dd, *J* = 5.5, 2.1 Hz, 2H), 3.01 (d, *J* = 5.1 Hz, 3H), 2.31 (s, 3H), 2.20 (s, 3H), 2.09 (s, 3H); ^13 ^C-NMR (CDCl_3_) *δ* 165.4, 164.5, 152.9, 152.6, 150.2, 148.0, 144.9, 141.8, 141.5, 139.6, 137.4, 129.1 (2 C), 128.4, 121.2 (2 C), 113.0, 108.5, 26.3, 19.1, 13.4, 12.9; HPLC retention time 18.7 min, purity 98.9%.

#### 4.1.7. N-Methyl-4-((2,4,5-trimethyl-6–(3-(3-(trifluoromethyl)phenyl)ureido)pyridin-3-yl)oxy)picolinamide (6)

Reaction time 12 h; 81% yield; White solid; *R*_f_ 0.36 (CH_2_Cl_2_:MeOH = 20:1); m.p. 244 °C; MS (ESI) *m/z* 474.2 [M + H]^+^; ^1^H-NMR (CDCl_3_) *δ* 12.49 (s, 1H), 8.42 (d, *J* = 5.6 Hz, 1H), 8.01 (dd, *J* = 9.9, 4.9 Hz, 1H), 7.93 (s, 1H), 7.73 (d, *J* = 8.2 Hz, 1H), 7.53 (d, *J* = 2.5 Hz, 1H), 7.45 (t, *J* = 7.9 Hz, 1H), 7.32 (d, *J* = 7.8 Hz, 1H), 7.15 (s, 1H), 6.91 (dd, *J* = 5.6, 2.6 Hz, 1H), 3.01 (d, *J* = 5.1 Hz, 3H), 2.33 (s, 3H), 2.24 (s, 3H), 2.10 (s, 3H); ^13 ^C-NMR (CDCl_3_) *δ* 165.4, 164.5, 152.9, 152.8, 150.2, 148.0, 144.9, 141.9, 141.5, 139.4, 131.8, 131.3, 129.6, 124.2 (d, *J* = 272.3 Hz), 123.0, 112.0 (q, *J* = 3.5 Hz), 117.4, 116.7 (q, *J* = 4.0 Hz), 113.0, 108.6, 26.3, 19.2, 13.4, 12.9; HPLC retention time 15.2 min, purity 99.8%.

#### 4.1.8. N-Methyl-4-((2,4,5-trimethyl-6-(3-phenylureido)pyridin-3-yl)oxy)picolinamide (7)

Reaction time 18 h; 76% yield; White solid; *R*_f_ 0.25 (CH_2_Cl_2_:MeOH = 20:1); m.p. 229 °C; MS (ESI) m/z 428.4 [M + Na]^+^; ^1^H-NMR (CDCl_3_) *δ* 12.19 (s, 1H), 8.41 (d, *J* = 5.5 Hz, 1H), 8.01 (d, *J* = 4.5 Hz, 1H), 7.57 (dd, *J* = 10.0, 5.0 Hz, 3H), 7.34 (t, *J* = 7.9 Hz, 2H), 7.08 (t, *J* = 7.4 Hz, 1H), 6.89 (dd, *J* = 5.5, 2.5 Hz, 2H), 3.01 (d, *J* = 5.1 Hz, 3H), 2.32 (s, 3H), 2.21 (s, 3H), 2.09 (s, 3H).; ^13 ^C-NMR (CDCl_3_) *δ* 165.4, 164.5, 152.8, 150.1, 148.1, 144.8, 141.7, 141.3, 138.6, 129.1 (2 C), 123.5, 120.1 (2 C), 117.0, 114.9, 112.9, 108.7, 26.3, 19.1, 13.4, 12.9; HPLC retention time 17.1 min, purity 98.5%.

#### 4.1.9. 4-((6-(3-(4-Fluorophenyl)ureido)-2,4,5-trimethylpyridin-3-yl)oxy)-N-methylpicolinamide (8)

Reaction time 48 h; 85% yield; White solid; *R*_f_ 0.26 (CH_2_Cl_2_:MeOH = 40:1); m.p. 253 °C; MS (ESI) *m/z* 446.1[M + Na]^+^; ^1^H-NMR (CDCl_3_) *δ* 12.19 (s, 1H), 8.41 (d, *J* = 5.6 Hz, 1H), 8.02 (d, *J* = 4.8 Hz, 1H), 7.66–7.48 (m, 3H), 7.14–6.97 (m, 3H), 6.90 (dd, *J* = 5.6, 2.6 Hz, 1H), 3.00 (d, *J* = 5.1 Hz, 3H), 2.30 (s, 3H), 2.21 (s, 3H), 2.09 (s, 3H); ^13 ^C-NMR (CDCl_3_) *δ* 165.4, 164.5, 161.0, 157.2, 152.9, 150.2, 148.2, 144.7, 141.4, 134.8, 134.7, 121.7, 121.6, 115.9, 115.5, 112.9, 108.6, 77.2, 26.3, 19.1, 13.4, 12.9; HPLC retention time 17.3 min, purity 99.4%.

#### 4.1.10. 4-((6–(3-(4-Bromophenyl)ureido)-2,4,5-trimethylpyridin-3-yl)oxy)-N-methylpicolinamide (9)

Reaction time 24 h; 95% yield; White solid; *R*_f_ 0.22 (CH_2_Cl_2_:MeOH = 20:1); m.p. 268 °C; MS (ESI) *m/z* 506.1 [M + Na]^+^; ^1^H-NMR (CDCl_3_) *δ* 12.27 (s, 1H), 8.42 (d, *J* = 5.5 Hz, 1H), 8.02 (s, 1H), 7.60–7.39 (m, 5H), 7.00–6.81 (m, 2H), 3.01 (d, *J* = 5.1 Hz, 3H), 2.32 (s, 3H), 2.21 (s, 3H), 2.10 (s, 3H); ^13 ^C-NMR (CDCl_3_) *δ* 165.4, 164.5, 152.9, 150.2, 148.0, 142.0, 141.5, 137.9, 135.8, 132.1 (2 C), 124.5, 121.6 (2 C), 116.0, 113.0, 108.5, 26.3, 19.1, 13.5, 12.9; HPLC retention time 19.0 min, purity 98.9%.

#### 4.1.11. N-Methyl-4-((2,4,5-trimethyl-6-(3-(4-nitrophenyl)ureido)pyridin-3-yl)oxy)picolinamide (10)

Reaction time 12 h; 74% yield; White solid; *R*_f_ 0.26 (CH_2_Cl_2_:MeOH = 40:1); m.p. 262 °C; MS (ESI) *m/z* 451.4 [M + H]^+^; ^1^H-NMR (CDCl_3_) *δ* 12.81 (s, 1H), 8.44 (d, *J* = 5.7 Hz, 1H), 8.24 (d, *J* = 9.1 Hz, 2H), 8.01 (d, *J* = 6.2 Hz, 1H), 7.75 (d, *J* = 9.2 Hz, 2H), 7.48 (d, *J* = 2.5 Hz, 1H), 7.00–6.87 (m, 2H), 3.01 (d, *J* = 5.1 Hz, 3H), 2.36 (s, 3H), 2.23 (s, 3H), 2.12 (s, 3H); HPLC retention time 17.4 min, purity 98.5%. Ditosylate salt **10–1** of compound **10** was prepared for further analyses.

#### 4.1.12. N-Methyl-4-((2,4,5-trimethyl-6-(3-(4-nitrophenyl)ureido)pyridin-3-yl)oxy)picolinamide ditosylate (10–1)

To a suspension of **10** (22.5 mg, 0.05 mmol) in ethanol (3 ml) was added *p*-toluenesulphonic acid (19.02 mg, 0.10 mmol). The mixture was stirred at 60 °C for 1 h and the resulting solution was concentrated to give **10–1** (100%). White solid; *R*_f_ 0.26 (CH_2_Cl_2_:MeOH = 40:1); ^1^H-NMR (CD_3_OD) *δ* 8.80 (d, *J* = 6.6 Hz, 1H), 8.20 (d, *J* = 9.1 Hz, 2H), 8.05 (s, 1H), 7.83 (dd, *J* = 6.6, 2.3 Hz, 1H), 7.74 (d, *J* = 9.1 Hz, 2H), 7.65 (d, *J* = 8.0 Hz, 5H), 7.19 (d, *J* = 7.8 Hz, 5H), 2.87 (s, 3H), 2.54 (s, 3H), 2.36 (s, 3H), 2.31 (s, 9H); ^13 ^C-NMR (CD_3_OD) *δ* 170.9, 160.1, 154.3, 152.8, 148.2, 147.2, 146.7, 144.8, 143.3, 142.9, 141.9 (2 C), 141.0, 129.9 (5 C), 126.9 (5 C), 125.9 (2 C), 125.8, 120.3 (2 C), 117.0, 112.0, 27.2, 21.3 (2), 15.5, 14.9, 13.3.

#### 4.1.13. N-Methyl-4-((2,4,5-trimethyl-6-(3-(4-(trifluoromethyl)phenyl)ureido)pyridin-3-yl)oxy)picolinamide (11)

Reaction time 48 h; 78% yield; White solid; *R*_f_ 0.27 (CH_2_Cl_2_:MeOH = 40:1); m.p. 254 °C; MS (ESI) *m/z* 474.2 [M + H]^+^; ^1^H-NMR (CDCl_3_) *δ* 12.60 (s, 1H), 8.41 (d, *J* = 5.6 Hz, 1H), 8.03 (dd, *J* = 9.5, 4.5 Hz, 1H), 7.69 (d, *J* = 8.6 Hz, 2H), 7.56 (d, *J* = 8.7 Hz, 2H), 7.51 (d, *J* = 2.6 Hz, 1H), 7.45 (s, 1H), 6.91 (dd, *J* = 5.6, 2.6 Hz, 1H), 2.99 (d, *J* = 5.1 Hz, 3H), 2.32 (s, 3H), 2.23 (s, 3H), 2.08 (s, 3H); ^13 ^C-NMR (CDCl_3_) *δ* 165.3, 164.5, 152.9, 152.8, 150.2, 148.0, 144.7, 142.0 (d, *J* = 1.2 Hz), 142.0, 141.5, 126.3 (q, *J* = 3.8 Hz), 125.3, 124.7, 124.4 (d, *J* = 271.4 Hz), 119.4 (2 C), 117.6, 113.0, 108.4, 26.3, 19.1, 13.4, 12.9; HPLC retention time 19.1 min, purity 99.9%.

#### 4.1.14. N-Methyl-4-((2,4,5-trimethyl-6-(3-(4-(trifluoromethoxy)phenyl)ureido)pyridin-3-yl)oxy)picolinamide (12)

Reaction time 48 h; 96% yield; White solid; *R*_f_ 0.26 (CH_2_Cl_2_:MeOH = 40:1); m.p. 185 °C; MS (ESI) 512.1 *m/z* [M + Na]^+^; ^1^H-NMR (CDCl_3_) *δ* 12.37 (s, 1H), 8.40 (d, *J* = 5.6 Hz, 1H), 8.01 (d, *J* = 5.0 Hz, 1H), 7.60 (d, *J* = 9.0 Hz, 2H), 7.51 (d, *J* = 2.5 Hz, 1H), 7.34 (s, 1H), 7.17 (d, *J* = 8.6 Hz, 2H), 6.90 (dd, *J* = 5.6, 2.6 Hz, 1H), 2.99 (d, *J* = 5.1 Hz, 3H), 2.30 (s, 3H), 2.22 (s, 3H), 2.08 (s, 3H); ^13 ^C-NMR (CDCl_3_) *δ* 165.4, 164.5, 153.0, 152.9, 150.1, 148.1, 144.7, 141.8, 141.4, 137.6, 122.7, 121.8, 120.9, 118.6, 117.5, 112.9, 108.5, 26.2, 19.0, 13.4, 12.8; HPLC retention time 19.3 min, purity 99.3%.

#### 4.1.15. 4-((6-(3-(4-Methoxyphenyl)ureido)-2,4,5-trimethylpyridin-3-yl)oxy)-N-methylpicolinamide (13)

Reaction time 12 h; 91% yield; White solid; *R*_f_ 0.29 (CH_2_Cl_2_:MeOH = 40:1); m.p. 258 °C; MS (ESI) *m/z* 436.2 [M + H]^+^; ^1^H-NMR (CDCl_3_) *δ* 11.98 (s, 1H), 8.40 (d, *J* = 5.5 Hz, 1H), 8.02 (s, 1H), 7.52 (dd, *J* = 13.9, 5.4 Hz, 3H), 7.02 (s, 1H), 6.97–6.83 (m, 1H), 3.80 (s, 3H), 3.00 (d, *J* = 5.1 Hz, 3H), 2.34–2.01 (m, 3H); ^13 ^C-NMR (CDCl_3_) *δ* 165.5, 164.5, 156.1, 153.0, 152.9, 150.1, 148.3, 144.7, 141.6, 141.3, 131.8, 121.8, 117.1, 114.4, 112.8, 108.7, 55.7, 26.3, 19.0, 13.4, 12.9; HPLC retention time 16.6 min, purity 98.2%.

#### 4.1.16. N-Methyl-4-((2,4,5-trimethyl-6-(3-(p-tolyl)ureido)pyridin-3-yl)oxy)picolinamide (14)

Reaction time 4 days; 74% yield; White solid; *R*_f_ 0.32 (CH_2_Cl_2_:MeOH = 20:1); m.p. 247 °C; MS (ESI) *m/z* 442.0 [M + Na]^+^; ^1^H-NMR (CDCl_3_) *δ* 12.14 (s, 1H), 8.39 (d, *J* = 5.6 Hz, 1H), 8.02 (d, *J* = 5.0 Hz, 1H), 7.55 (d, *J* = 2.5 Hz, 1H), 7.46 (d, *J* = 8.3 Hz, 2H), 7.27 (d, *J* = 3.1 Hz, 1H), 7.12 (d, *J* = 8.2 Hz, 2H), 6.86 (dd, *J* = 5.6, 2.6 Hz, 1H), 2.99 (d, *J* = 5.1 Hz, 3H), 2.30 (d, *J* = 3.3 Hz, 6H), 2.21 (s, 3H), 2.07 (s, 3H); ^13 ^C NMR (CDCl_3_) *δ* 165.4, 164.5, 153.0, 152.8, 150.1, 148.3, 144.6, 141.5, 141.2, 136.1, 132.9, 129.5 (2 C), 120.0 (2 C), 117.2, 112.7, 108.7, 26.2, 20.9, 19.0, 13.3, 12.9; HPLC retention time 18.1 min, purity 99.2%.

#### 4.1.17. 4-((6-(3-(4-Isopropylphenyl)ureido)-2,4,5-trimethylpyridin-3-yl)oxy)-N-methylpicolinamide (15)

Reaction time 7 days; 61% yield; White solid; *R*_f_ 0.30 (CH_2_Cl_2_:MeOH = 20:1); m.p. 188 °C; MS (ESI) *m/z* 448.2 [M + H]^+^; ^1^H-NMR (CDCl_3_) *δ* 12.17 (s, 1H), 8.38 (d, *J* = 5.5 Hz, 1H), 8.04 (d, *J* = 4.8 Hz, 1H), 7.55 (d, *J* = 2.1 Hz, 2H), 7.49 (d, *J* = 7.9 Hz, 2H), 7.18 (d, *J* = 7.8 Hz, 2H), 6.86 (dd, *J* = 5.3, 2.2 Hz, 1H), 2.99 (d, *J* = 5.0 Hz, 3H), 2.93–2.77 (m, 1H), 2.28 (s, 3H), 2.22 (s, 3H), 2.06 (s, 3H), 1.23 (d, *J* = 6.9 Hz, 6H).; ^13 ^C-NMR (CDCl_3_) *δ* 165.3, 164.4, 152.7, 150.0, 148.3, 144.4, 144.0, 141.5, 141.1, 136.3, 126.9, 120.1, 117.4, 112.7, 108.7, 33.6, 26.2, 24.1, 18.9, 13.3, 12.9; HPLC retention time 20.0 min, purity 98.8%.

#### 4.1.18. 4-((6-(3-(2-(Tert-Butyl)-6-methylphenyl)ureido)-2,4,5-trimethylpyridin-3-yl)oxy)-N-methylpicolinamide (16)

Reaction time 7 days; 90% yield; White solid; *R*_f_ 0.26 (CH_2_Cl_2_:MeOH = 40:1); m.p. 206 °C; MS (ESI) *m/z* 498.3 [M + Na]^+^; ^1^H-NMR (CDCl_3_) *δ* 11.33 (s, 1H), 8.41 (d, *J* = 5.6 Hz, 1H), 8.04 (d, *J* = 4.9 Hz, 1H), 7.51 (d, *J* = 2.5 Hz, 1H), 7.31 (dt, *J* = 7.6, 3.8 Hz, 1H), 7.20–7.13 (m, 2H), 7.05 (s, 1H), 6.95 (dd, *J* = 5.6, 2.6 Hz, 1H), 3.00 (d, *J* = 5.1 Hz, 3H), 2.34 (s, 3H), 2.18 (d, *J* = 9.1 Hz, 6H), 2.08 (s, 3H), 1.45 (s, 9H); ^13 ^C-NMR (CDCl_3_) *δ* 165.4, 164.5, 153.6, 152.7, 150.0, 148.4, 147.3, 145.0, 141.4, 141.2, 138.6, 133.9, 129.0, 127.2, 124.5, 116.7, 113.2, 108.3, 35.2, 31.1, 26.2, 19.0, 18.6, 13.4, 12.9; HPLC retention time 19.5 min, purity 98.2%.

#### 4.1.19. N-Methyl-4-((2,4,5-trimethyl-6-(3-(naphthalen-1-yl)ureido)pyridin-3-yl)oxy)picolinamide (17)

Reaction time 8 h; 78% yield; White solid; *R*_f_ 0.29 (CH_2_Cl_2_:MeOH = 20:1); m.p. 266 °C; MS (ESI) m/z 478.0 [M + H]^+^; ^1^H-NMR (CDCl_3_) *δ* 12.49 (s, 1H), 8.42 (d, *J* = 5.4 Hz, 1H), 8.26 (d, *J* = 8.0 Hz, 2H), 8.08–7.96 (m, 1H), 7.93–7.83 (m, 1H), 7.65 (d, *J* = 7.9 Hz, 1H), 7.61–7.42 (m, 4H), 7.07–6.83 (m, 2H), 3.02 (d, *J* = 4.9 Hz, 3H), 2.39 (s, 3H), 2.26 (s, 3H), 2.13 (s, 3H)); HPLC retention time 15.2 min, purity 98.9%. Ditosylate salt **17–1** of compound **17** was prepared for further analyses. *N-Methyl-4-((2,4,5-trimethyl-6–(3-(naphthalen-1-yl)ureido)pyridin-3-yl)oxy)picolinamide ditosylate (****17–1****)* To a suspension of **17** (30 mg, 0.066 mmol) in ethanol (3 ml) was added *p*-toluenesulphonic acid (25 mg, 0.132 mmol). The mixture was stirred at 60 °C for 1 h and the resulting solution was concentrated to give **17–1** (100%). White solid; *R*_f_ 0.29 (CH_2_Cl_2_:MeOH = 20:1); ^1^H-NMR (CD_3_OD) *δ* 8.75 (d, *J* = 6.1 Hz, 1H), 8.18–8.04 (m, 1H), 7.92 (d, *J* = 7.8 Hz, 3H), 7.79 (d, *J* = 7.8 Hz, 1H), 7.67 (d, *J* = 7.5 Hz, 5H), 7.58–7.46 (m, 3H), 7.17 (d, *J* = 7.4 Hz, 4H), 2.90 (s, 3H), 2.44 (d, *J* = 10.9 Hz, 6H), 2.30 (s, 9H); ^13 ^C-NMR (CD_3_OD) *δ* 170.0, 161.4, 155.8, 152.4, 149.4, 148.0, 147.8, 143.5, 142.6, 141.8, 140.3, 135.7, 132.8, 129.8 (5 C), 129.7, 128.7, 127.7, 127.4, 127.3, 126.9 (5 C), 126.6, 124.8, 122.3, 121.8, 116.4, 111.5, 27.1, 21.1 (2 C), 15.5, 14.7, 13.2.

#### 4.1.20. 4-((6-(3-Benzylureido)-2,4,5-trimethylpyridin-3-yl)oxy)-N-methylpicolinamide (18)

Reaction time 8 days; 83% yield; White solid; *R*_f_ 0.26 (CH_2_Cl_2_:MeOH = 40:1); m.p. 195 °C; MS (ESI) *m/z* 420.9 [M + H]^+^; ^1^H-NMR (CDCl_3_) *δ* 10.12 (s, 1H), 8.36 (d, *J* = 5.5 Hz, 1H), 8.01 (d, *J* = 4.6 Hz, 1H), 7.50 (d, *J* = 2.2 Hz, 1H), 7.42–7.28 (m, 4H), 7.22 (d, *J* = 6.9 Hz, 1H), 7.09 (s, 1H), 6.84 (dd, *J* = 5.5, 2.5 Hz, 1H), 4.61 (d, *J* = 5.4 Hz, 2H), 2.98 (d, *J* = 5.1 Hz, 3H), 2.14 (s, 3H), 2.10 (s, 3H), 2.03 (s, 3H); ^13 ^C NMR (CDCl_3_) *δ* 165.5, 164.5, 155.6, 152.7, 150.0, 148.5, 144.8, 141.0, 139.1, 128.7, 127.4, 127.2, 116.7, 112.8, 108.6, 44.1, 26.2, 18.9, 13.2, 12.8; HPLC retention time 13.0 min, purity 99.8%.

#### 4.1.21. N-Methyl-4-((2,4,5-trimethyl-6-(3-propylureido)pyridin-3-yl)oxy)picolinamide (19)

Reaction time 18 days; 84% yield; White solid; *R*_f_ 0.23 (CH_2_Cl_2_:MeOH = 30:1); m.p. 207 °C; MS (ESI) *m/z* 394.2 [M + Na]^+^; ^1^H-NMR (CDCl_3_) *δ* 9.68 (s, 1H), 8.37 (d, *J* = 5.6 Hz, 1H), 8.01 (d, *J* = 4.8 Hz, 1H), 7.49 (d, *J* = 2.5 Hz, 1H), 6.86 (dd, *J* = 5.6, 2.6 Hz, 2H), 3.35 (dd, *J* = 12.5, 6.7 Hz, 2H), 2.98 (d, *J* = 5.1 Hz, 3H), 2.19 (s, 3H), 2.14 (s, 3H), 2.04 (s, 3H), 1.72–1.53 (m, 2H), 0.99 (t, *J* = 7.4 Hz, 3H); ^13 ^C-NMR (CDCl_3_) *δ* 165.5, 164.6, 155.5, 152.8, 150.1, 148.7, 144.8, 140.9 (2 C), 116.5, 112.9, 108.6, 41.8, 26.3, 23.2, 19.1, 13.3, 12.9, 11.8; HPLC retention time 15.0 min, purity 97.6%.

#### 4.1.22. 4-((6-(3-Cyclopropylureido)-2,4,5-trimethylpyridin-3-yl)oxy)-N-methylpicolinamide (20)

Reaction time 15 h; 71% yield; White solid; *R*_f_ 0.25 (CHCl_3_:MeOH = 50:1); m.p. 192 °C; MS (ESI) *m/z* 370.2 [M + H]^+^; ^1^H-NMR (CDCl_3_) *δ* 9.77 (s, 1H), 8.36 (d, *J* = 5.6 Hz, 1H), 8.01 (d, *J* = 4.6 Hz, 1H), 7.46 (d, *J* = 2.5 Hz, 1H), 6.89 (s, 1H), 6.85 (dd, *J* = 5.6, 2.6 Hz, 1H), 2.97 (d, *J* = 5.1 Hz, 3H), 2.80 (ddd, *J* = 10.3, 6.1, 2.5 Hz, 1H), 2.16 (s, 3H), 2.11 (s, 3H), 2.02 (s, 3H), 0.84–0.73 (m, 2H), 0.63–0.54 (m, 2H); ^13 ^C-NMR (CDCl_3_) *δ* 165.4, 164.5, 156.6, 152.7, 150.0 (2 C), 148.4, 144.7, 141.0, 140.9, 116.7, 112.9, 108.5, 77.2, 26.2, 22.5, 19.0, 13.2, 12.8, 6.7; HPLC retention time 14.0 min, purity 97.1%.

#### 4.1.23. 4-((6-(3-Benzoylureido)-2,4,5-trimethylpyridin-3-yl)oxy)-N-methylpicolinamide (21)

Reaction time 48 h; 58% yield; White solid; *R*_f_ 0.20 (CH_2_Cl_2_:MeOH = 40:1); m.p. 177 °C; MS (ESI) *m/z* 434.2 [M + H]^+^; ^1^H-NMR (CDCl_3_) *δ* 10.85 (s, 1H), 8.34 (d, *J* = 5.6 Hz, 1H), 8.01 (t, *J* = 6.2 Hz, 3H), 7.62 (s, 1H), 7.52 (t, *J* = 7.3 Hz, 1H), 7.39 (t, *J* = 7.5 Hz, 2H), 6.76 (dd, *J* = 5.2, 2.1 Hz, 1H), 2.98 (d, *J* = 5.1 Hz, 3H), 2.25 (s, 3H), 2.19 (s, 3H), 2.05 (s, 3H); ^13 ^C-NMR (CDCl_3_) *δ* 168.5, 165.1, 164.5, 152.8, 152.2, 150.1, 147.7, 145.5, 144.7, 141.2, 133.2, 132.4, 128.8 (2 C), 128.1 (2 C), 126.9, 112.4, 109.2, 26.2, 19.1, 14.6, 13.3; HPLC retention time 15.0 min, purity 98.5%.

#### 4.1.24. N-Methyl-4-((2,4,5-trimethyl-6-(3-tosylureido)pyridin-3-yl)oxy)picolinamide (22)

Reaction time 72 h; 80% yield; White solid; *R*_f_ 0.29 (CH_2_Cl_2_:MeOH = 20:1); m.p. 187 °C; MS (ESI) *m/z* 506.1 [M + Na]^+^; ^1^H-NMR (CDCl_3_) *δ* 13.71 (s, 1H), 8.42 (d, *J* = 5.6 Hz, 1H), 8.00 (d, *J* = 8.2 Hz, 3H), 7.59–7.41 (m, 2H), 7.32 (d, *J* = 8.2 Hz, 2H), 6.92 (dd, *J* = 5.5, 2.5 Hz, 1H), 3.01 (d, *J* = 5.1 Hz, 3H), 2.42 (s, 3H), 2.30 (s, 3H), 2.15 (s, 3H), 2.08 (s, 3H); ^13 ^C-NMR (CDCl_3_) *δ* 165.1, 164.5, 152.8, 150.6, 150.2, 146.7, 145.3, 144., 142.7, 142.1, 136.8, 129.6 (2 C), 128.5 (2 C), 118.1, 113.1, 108.3, 26.3, 21.8, 18.9, 13.5, 12.8; HPLC retention time 15.8 min, purity 97.5%.

#### 4.1.25. 4-((6-(3-(4-Aminophenyl)ureido)-2,4,5-trimethylpyridin-3-yl)oxy)-N-methylpicolinamide (23)

To a solution of **10** (23 mg, 0.05 mmol) in a mixed solvent of ethanol-chloroform (1:1, 2 ml) was added tin(II) chloride dihydrate (57 mg, 0.26 mmol). The mixture was refluxed for 12 h and concentrated. The residue was diluted with chloroform and the pH of the mixture was adjusted to 8 using saturated NaHCO_3_. After extraction of the mixture with chloroform, the chloroform layer was washed with brine, dried over anhydrous magnesium sulphate, and concentrated. The residue was purified by silica gel column chromatography (CH_2_Cl_2_:MeOH =100:1 to 30:1) to give **23** (13 mg, 62%). White solid; *R*_f_ 0.21 (CH_2_Cl_2_:MeOH = 30:1); m.p. 205 °C; MS (ESI) *m/z* 421.2 [M + H]^+^; ^1^H-NMR (CDCl_3_) *δ* 11.86 (s, 1H), 8.40 (d, *J* = 5.6 Hz, 1H), 8.13–7.89 (m, 1H), 7.55 (d, *J* = 2.5 Hz, 1H), 7.35 (d, *J* = 8.7 Hz, 2H), 6.94–6.82 (m, 2H), 6.69 (d, *J* = 8.7 Hz, 2H), 3.58 (s, 2H), 3.01 (d, *J* = 5.1 Hz, 3H), 2.28 (s, 3H), 2.18 (s, 3H), 2.08 (s, 3H); ^13 ^C-NMR (CDCl_3_) *δ* 165.5, 164.6, 153.1, 152.8, 150.1, 148.3, 144.8, 142.8, 141.4, 141.2, 130.0, 122.2, 116.8, 115.8, 112.8, 108.8, 26.3, 19.1, 13.4, 12.9; HPLC retention time 13.8 min, purity 91.1%.

#### 4.1.26. 1-(5-(Benzyloxy)-3,4,6-trimethylpyridin-2-yl)-3-(4-chloro-3-(trifluoromethyl)phenyl)urea (24)

To a solution of **1** (100 mg, 0.41 mmol) in dichloromethane (4 ml) was added 4-chloro-3-(trifluoromethyl)phenyl isocyanate (110 mg, 0.5 mmol). The mixture was stirred at room temperature for 2 h. The reaction mixture was filtered and thoroughly washed with ethyl acetate to give **24** (158 mg, 81%). White solid; *R*_f_ 0.25 (hexanes:EtOAc = 5:1); m.p. 206 °C; MS (ESI) *m/z* 464.1 [M + H]^+^; ^1^H-NMR (CDCl_3_) *δ* 12.74 (s, 1H), 7.90 (d, *J* = 2.3 Hz, 1H), 7.77 (dd, *J* = 8.7, 2.3 Hz, 1H), 7.48–7.37 (m, 6H), 6.78 (s, 1H), 4.78 (s, 2H), 2.50 (s, 3H), 2.27 (s, 3H), 2.17 (s, 3H); HPLC retention time 18.8 min, purity 99.1%. Monotosylate salt **24–1** of compound **24** was prepared for further analyses.

#### 4.1.27. 1-(5-(Benzyloxy)-3,4,6-trimethylpyridin-2-yl)-3-(4-chloro-3-(trifluoromethyl)phenyl)urea monotosylate (24–1)

To a suspension of **24** (30 mg, 0.0647 mmol) in ethanol (2 ml) was added *p*-toluenesulphonic acid (12.3 mg, 0.0647 mmol). The mixture was stirred at 60 °C for 1 h, and concentrated to give **24–1** (100%). White solid; R_f_ 0.31 (CH_2_Cl_2_:MeOH = 20:1); ^1^H-NMR (CD_3_OD) *δ* 8.08 (d, *J* = 2.5 Hz, 1H), 7.68 (dd, *J* = 10.6, 5.4 Hz, 3H), 7.55 (d, 1H), 7.43 (dt, *J* = 7.1, 2.9 Hz, 5H), 7.20 (d, *J* = 8.0 Hz, 2H), 4.97 (s, 2H), 2.54 (s, 3H), 2.44 (s, 3H), 2.39–2.30 (m, 6H); ^13 ^C NMR (CD_3_OD) *δ* 154.7, 154.6, 150.0, 143.8, 141.8, 141.7, 141.6, 138.4, 137.2, 133.3, 129.9, 129.8 (3 C), 129.8 (2 C), 129.7, 129.4, 127.4, 126.9 (2 C), 126.3, 125.1, 122.0, 119.6 (dd, *J* = 11.7, 5.9 Hz), 77.5, 21.3, 15.4, 14.9, 13.1.

#### 4.1.28. 1-(4-Chloro-3-(trifluoromethyl)phenyl)-3-(5-hydroxy-3,4,6-trimethylpyridin-2-yl)urea (25)

To a solution of **24** (114 mg, 0.25 mmol) in dichloromethane (6 ml) was added pentamethylbenzene (111 mg, 0.75 mmol). 1 M boron trichloride in dichloromethane (0.5 ml, 0.5 mmol) was added dropwise to the mixture in iced-bath and was allowed to stir for 30 min. The reaction was quenched with 10 ml of chloroform and methanol (9:1). The mixture was concentrated and the residue was purified by silica gel column chromatography (CH_2_Cl_2_:MeOH =50:1) to give **25** (80 mg, 87%). White solid; R_f_ 0.30 (CH_2_Cl_2_:MeOH = 40:1); m.p. 242 °C; MS (ESI) *m/z* 374.5 [M + H]^+^; ^1^H-NMR (DMSO-d_6_) *δ* 11.19 (s, 1H), 8.42 (d, *J* = 10.7 Hz, 2H), 8.14 (d, *J* = 2.4 Hz, 1H), 7.67 (dd, *J* = 8.8, 2.3 Hz, 1H), 7.59 (d, *J* = 8.8 Hz, 1H), 2.38 (s, 3H), 2.15 (s, 3H), 2.11 (s, 3H); ^13 ^C-NMR (DMSO-d_6_) *δ* 152.8, 145.7, 141.6, 139.6, 139.0, 135.4, 131.8, 126.6 (q, *J* = 30.7 Hz), 123.2, 122.6 (d, *J* = 273.1 Hz), 122.3 (d, *J* = 1.6 Hz), 120.9, 117.0 (q, *J* = 5.7 Hz), 18.8, 13.2, 12.4; HPLC retention time 18.8 min, purity 98.5%.

#### 4.1.28. 4-((2-Amino-4,6-dimethylpyrimidin-5-yl)oxy)-N-methylpicolinamide (27)

To a solution of **26** (338 mg, 2.43 mmol) in *N*,*N*-dimethylformamide was added potassium *tert*-butoxide (436 mg, 3.88 mmol. After stirring for 30 min, was added 4-chloro-*N*-methyl-2-pyridinecarboxamide (456 mg, 2.66 mmol) and microwaved with 200 W for 4.5 h at 200 °C. The reaction mixture was diluted with ethyl acetate and water, and the aqueous layer was extracted using ethyl acetate. Organic layer was dried over anhydrous magnesium sulphate and concentrated. The residual solid was purified by fractional crystallisation with dichloromethane to **27** (528 mg, 80%). Beige solid; *R*_f_. 0.20 (CH_2_Cl_2_:MeOH = 20:1); m.p. 188 °C; MS (ESI) *m/z* 274 [M + H]^+^; ^1^H-NMR (CDCl_3_) *δ* 8.32 (d, *J* = 5.6 Hz, 1H), 8.03 (d, *J* = 4.9 Hz, 1H), 7.57 (d, *J* = 2.5 Hz, 1H), 6.80 (dd, *J* = 5.6, 2.6 Hz, 1H), 5.63 (s, 2H), 2.94 (d, *J* = 5.1 Hz, 3H), 2.07 (s, 6H); ^13 ^C-NMR (CDCl_3_) *δ* 165.3, 164.4, 160.9, 160.3, 152.7, 150.0, 137.5, 112.4, 108.8, 26.2, 18.7 (2C).

#### 4.1.29. 4-((2-(3-(4-Chloro-3-(trifluoromethyl)phenyl)ureido)-4,6-dimethylpyrimidin-5-yl)oxy)-N-methylpicolinamide (28)

To a solution of **27** (50 mg, 0.18 mmol) in dichloromethane (4 ml) was added 4-chloro-3-(trifluoromethyl)phenyl isocyanate (53 mg, 0.24 mmol). The mixture was stirred at room temperature for 8 h. After concentration of the reaction mixture, the residue was purified by silica gel column chromatography (CH_2_Cl_2_:MeOH =50:1 to 25:1) to give **28** (51 mg, 58%). White solid; R_f_ 0.25 (CH_2_Cl_2_:MeOH = 30:1); m.p. 251 °C; MS (ESI) *m/z* 517.1 [M + Na]^+^; ^1^H-NMR (CDCl_3_) *δ* 11.58 (s, 1H), 8.47 (d, *J* = 5.6 Hz, 1H), 8.03 (dd, *J* = 9.2, 4.8 Hz, 1H), 7.91 (s, 1H), 7.74 (d, *J* = 8.5 Hz, 1H), 7.56 (d, *J* = 2.6 Hz, 1H), 7.45 (d, *J* = 8.6 Hz, 1H), 6.96 (dd, *J* = 5.6, 2.6 Hz, 1H), 3.02 (d, *J* = 5.1 Hz, 3H), 2.34 (s, 6H); HPLC retention time 18.5 min, purity 98.7%. Trifluoroacetate salt **28–1** of compound **28** was prepared for further analyses.

#### 4.1.30. 4-((2-(3-(4-Chloro-3-(trifluoromethyl)phenyl)ureido)-4,6-dimethylpyrimidin-5-yl)oxy)-N-methylpicolinamide trifluoroacetic acid (28–1)

To a suspension of **28** (10 mg, 0.020 mmol) in chloroform (1 ml) was added TFA in excess amount. The mixture was stirred at r.t. for 1 h and the resulting solution was concentrated to give **28–1** (100%). White solid; R_f_ 0.25 (CH_2_Cl_2_:MeOH = 30:1); ^1^H-NMR (CDCl_3_) *δ* 11.71 (s, 1H), 8.50 (d, *J* = 5.7 Hz, 1H), 8.24 (d, *J* = 5.4 Hz, 1H), 7.87 (d, *J* = 2.6 Hz, 1H), 7.73 (dd, *J* = 8.7, 2.6 Hz, 1H), 7.59 (d, *J* = 2.5 Hz, 1H), 7.47 (d, *J* = 8.7 Hz, 1H), 7.01 (dd, *J* = 5.7, 2.6 Hz, 1H), 3.04 (d, *J* = 5.1 Hz, 3H), 2.35 (s, 6H); ^13 ^C-NMR (CDCl_3_) *δ* 165.0, 164.5, 160.0 (q, *J* = 41.3 Hz), 153.9, 152.5, 152.3, 150.3, 140.2, 136.7, 132.2, 129.0 (q, *J* = 31.4 Hz), 127.0 (d, *J* = 2.2 Hz), 124.3, 119.3 (q, *J* = 5.6 Hz), 118.9 (d, *J* = 496.2 Hz), 118.8 (d, *J* = 1055.6 Hz), 113.3, 108.8, 26.7, 19.2 (2C).

### 4.2. Biological evaluation

#### 4.2.1. Cell culture

Hepatocellular carcinoma cell lines (HepG2, Huh7), human pancreatic ductal epithelial cell line (H6c7), and normal human colon cell line (CCD841) were obtained from the American Type Culture Collection (ATCC) (Manassas, VA, USA). Cells were cultured in DMEM/High Glucose media (Hep3B, Huh7, CCD841) or MEM/EBSS media (HepG2) containing 10% FBS, 100 IU/ml of penicillin and 100 µg/ml of streptomycin. H6c7 cells were cultured in keratinocyte serum free media supplemented with epidermal growth factor and bovine pituitary extract. Cells were maintained in 5% CO_2_ humidified atmosphere at 37 °C.

#### 4.2.2. Raf kinase assay

Kinase assays with 4 different Raf kinases were performed by Reaction Biology Corporation using Kinase HotSpot^TM^ assay platform (www.reactionbiology.com). Each human Raf kinase, A-Raf, B-Raf, B-Raf (V600E), or C-Raf (5–10 mU), was separately prepared in 25 µl reaction buffer solution with substrate (MEK1) in a concentration of 5, 3, and 1 µM, respectively. Compounds were delivered into each Raf reaction, followed ∼20 min later by addition of a mixture of ATP and [γ-^33^P-ATP] (specific activity approx. 500 cpm/pmol, concentration as required) to a final concentration of 10 µM. After incubation for 40 min at 25 °C, the reaction was stopped by the addition of a 3% phosphoric acid solution. Then, the reaction was spotted onto a P30 filtermat, and unbound phosphate was removed by washing three times for 5 min in 75 mM phosphoric acid and once in methanol prior to drying and scintillation counting. The background counting derived from control reactions containing inactive enzyme was subtracted, and specific kinase activity data were expressed as the percent remaining kinase activity in test compounds compared to vehicle (dimethyl sulphoxide) reactions. IC_50_ values and curve fits were obtained using Prism (GraphPad Software, La Jolla, CA).

#### 4.2.3. Vegfr-2 kinase assay

The VEGFR-2 (KDR) kinase assay was performed with a KDR (Promega, WI, USA) kinase enzyme system using the ADP-glow kinase assay kit (Promega) in accordance with the manufacturer's instructions. Kinase activity was detected by the addition 50 µM ATP to a mixture of 0.2 µg/µl poly (Glu4, Tyr1), test drug, and 1.5 ng/µl KDR enzyme. The reaction was carried out at 25 °C for 1 h in a total volume of 25 µl. Subsequently, 25 µl of ADP-Glow reagent was added to the mixture, and then incubated for 40 min at 25 °C. After the addition of 50 µl of ADP detection reagents for 30 min at 25 °C, luminescence was measured using a Fluostar Omega microplate reader (BMG LABTECH GmbH, Ortenberg, Germany).

#### 4.2.4. Cell proliferation assay

Cells were seeded in a 96-well plate and allowed to adhere for 24 h. The cells were serum starved in 1% serum containing medium for 12 h, and then treated with drugs in 10% serum-containing medium. After incubation for 48 h, cell proliferation was measured using MTT assay. Optical density was measured at 540 nm using a Spectrostar Nano microplate reader (BMG LABTECH GmbH, Ortenberg, Germany).

#### 4.2.5. Cytotoxicity assay

Cells were seeded in a 96-well plate and allowed to adhere for 24 h. Cells in low serum (1%)-containing medium were treated with drugs for 48 h. In case of H6c7 cells, cells in keratinocyte serum free media were treated with drugs for 48 h. Then, cell viability was measured using MTT assay. Optical density was measured at 540 nm using a Spectrostar Nano microplate reader (BMG LABTECH).

#### 4.2.6. Sphere formation assay

Sphere forming ability of Hep3B and Huh7 cells were examined after incubation with drugs for 48 h. Briefly, 3 × 10^3^ cells which were pre-treated with or without drugs in prEGM media (Lonza, Basel, Switzerland) were seeded on an ultra-low adhering 24 well plate (Corning Incorporated Costar, Corning, NY). Twice a week, 500 µL of used medium was replaced with fresh prEGM media. At 13 days after plating, images of spheres were captured using Celloger Mini (Curiosis Inc., Seoul, South Korea). The number of spheroids over 50 µm in diameter was counted by using Image J software (National Institute of Health, Bethesda, MD).

#### 4.2.7. Antitumour activity measurement in Huh7-xenografted CAM tumour model

Cancer cell xenograft onto chick chorioallantoic membrane (CAM), an *in vivo* tumour model, was used to evaluate anti-tumour efficacy of compounds. Fertilised chicken eggs purchased from Byeolbichon Farm (Gyeongbuk, South Korea) were incubated at 37 °C with 55% relative humidity. On day 9 of egg incubation (embryonic day 9, E9), a small hole was made in the shell over the air sac after the selection of bifurcated vessels. Another hole was made on the broadside using a needle by applying negative pressure from the wider part, creating false air sac. Using a grinding wheel (Dremel, Racine, WI), a small window (1 cm^2^) was created in the eggshell above the false air sac. For generation of xenograft CAM tumour model, Huh7 cells (1.5 × 10^6^ cells/CAM) were inoculated on the CAM with or without test drugs (10 µM final concentration). On day 13 of egg incubation (E13), tumour tissues attached to CAM were resected from the embryo and harvested. Photographs of tumour as well as blood vessel branches were taken using optical microscope (Olympus Corporation, Tokyo, Japan). Tumour tissues were weighted and the number of vessel branch points within the tumour region was counted.

Chick embryo experiments were approved beforehand by the Institutional Animal Care and Use Committee of Yeungnam University and were performed accordingly the guidelines issued by the Institute of Laboratory Animal Resources (1996) and Yeungnam University (The care and use of animals 2009).

### 4.3. Docking study

Small molecules were prepared into 3D coordinates considering tautomerization and electrostatic charge using LigPrep and Epik from Schrödinger. Docking screen with sorafenib, compound **4**, and compound **6** against the coordinates of B-Raf followed. Glide-SP^36^ of Schrödinger package (Schrödinger, LLC, New York, NY) was used as a docking engine and score. Intermolecular interactions were compared and visualised using the Grapheme Toolkit of Openeye package. The protocols in the previous study were employed for MMPBSA approach^50^. In brief, the SQM and LEaP programs in AMBER 18 generated the GAFF force fields of small molecules for the molecular dynamics (MD) simulation, whereas the ff14SB force field was employed for B-Raf (A chain of PDB code, 1UWJ). Four stages of minimisation, heating, equilibrium, and production comprised a single run. After 1500 cycles of minimisation, the heat of the system was elevated from 0 K to 300 K at a constant volume for 50 ps. The equilibrium step consisted of a constant pressure (1 atm) and temperature (300 K) continued for the next 100 ps. Of the 20 ns MD simulation, the latter 10 ns trajectories were employed for “MMPBSA.py” analyses^39^ to extract the change of free energy from the data. The GPU version of PMEMD generated the trajectory through MD simulation.

## Supplementary Material

Supplemental Material
