# Supplementary material for: Synthesis and anti-hepatocellular carcinoma activity of aminopyridinol–sorafenib hybrids
Source: J Enzyme Inhib Med Chem. 2021 Aug 2;36(1):1884–97. doi: 10.1080/14756366.2021.1953997 (PMC8344761; doi:10.1080/14756366.2021.1953997)
Supplement: Supplemental Material [file IENZ_A_1953997_SM8279.pdf]

Supplemental material

Synthesis and anti-hepatocellular carcinoma activity of aminopyridinol-sorafenib hybrids

Bhuwan Prasad Awasthi[a], Prakash Chaudhary[a], Diwakar Guragain[a], Jun-Goo Jee[b], Jung-Ae Kim[a], Byeong-Seon Jeong[a]

[a]*College of Pharmacy, Yeungnam University, Gyeongsan 38541, Republic of Korea*

[b]*College of Pharmacy, Kyungpook National University, Daegu 41566, Republic of Korea*

Correspondence

J.-G. Jee: Tel +82-53-950-8568; E-mail jjee@knu.ac.kr.

Jung-Ae Kim: Tel.: +82 53 810 2816; E-mail: jakim@yu.ac.kr

Byeong-Seon Jeong: Tel.: +82 53 810 2814; E-mail: jeongb@ynu.ac.kr

Contents

- ^{1}H and ^{13}C NMR spectra for all new compounds. (p2~29)
- HPLC chromatograms of the bio-tested compounds. (p30~41)
- Pose reproducibility in docking simulation. (Figure S1) (p42)
- Enrichments of true positives in docking simulation. (Figure S2) (p43)
- MMPBSA analyses of sorafenib, compounds 4 and 6. (Figure S3) (p44)

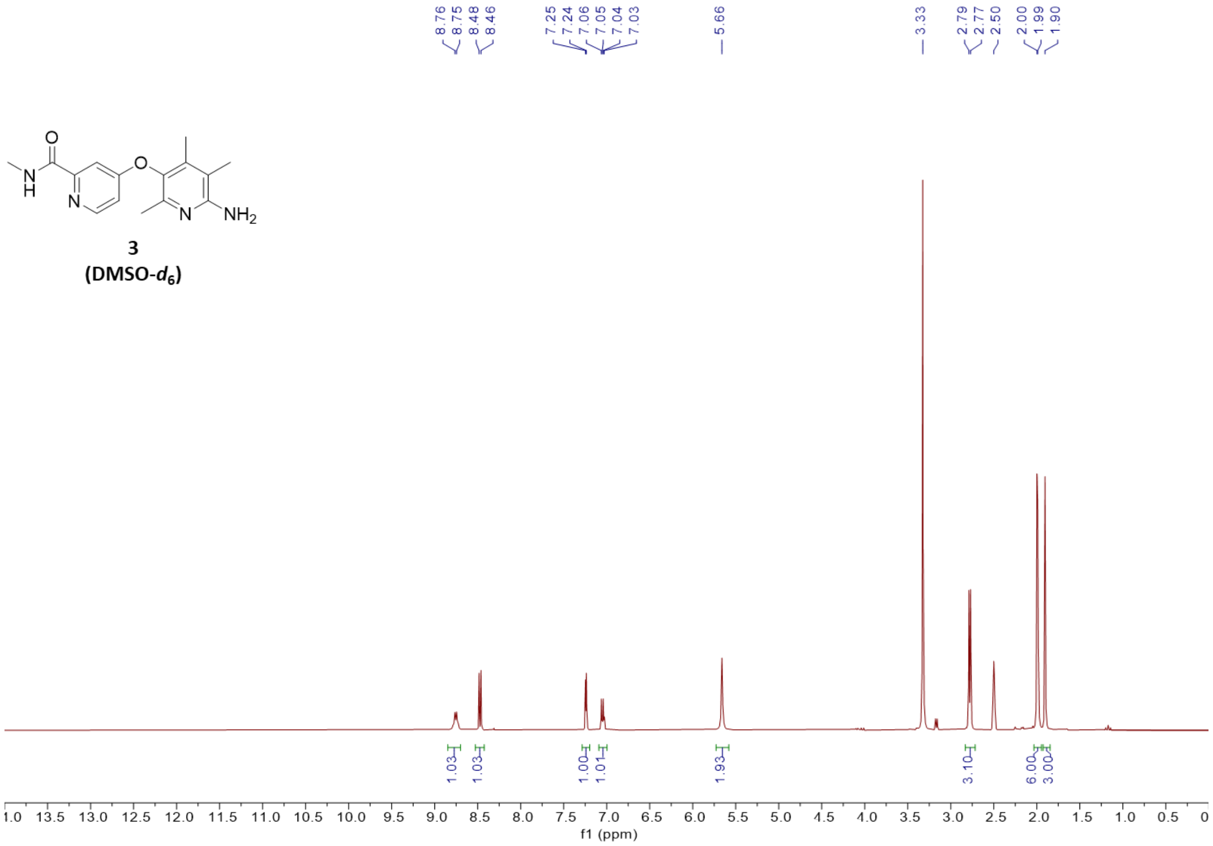
8.76
8.75
8.48
8.46
7.25
7.24
7.06
7.05
7.04
7.03
5.66
3.33
2.79
2.77
2.50
2.00
1.99
1.90
O
N
H
N
O
N
NH_2
3
(DMSO-d_6)
1.03
1.03
1.00
1.01
1.93
3.10
6.00
3.00
f1 (ppm)

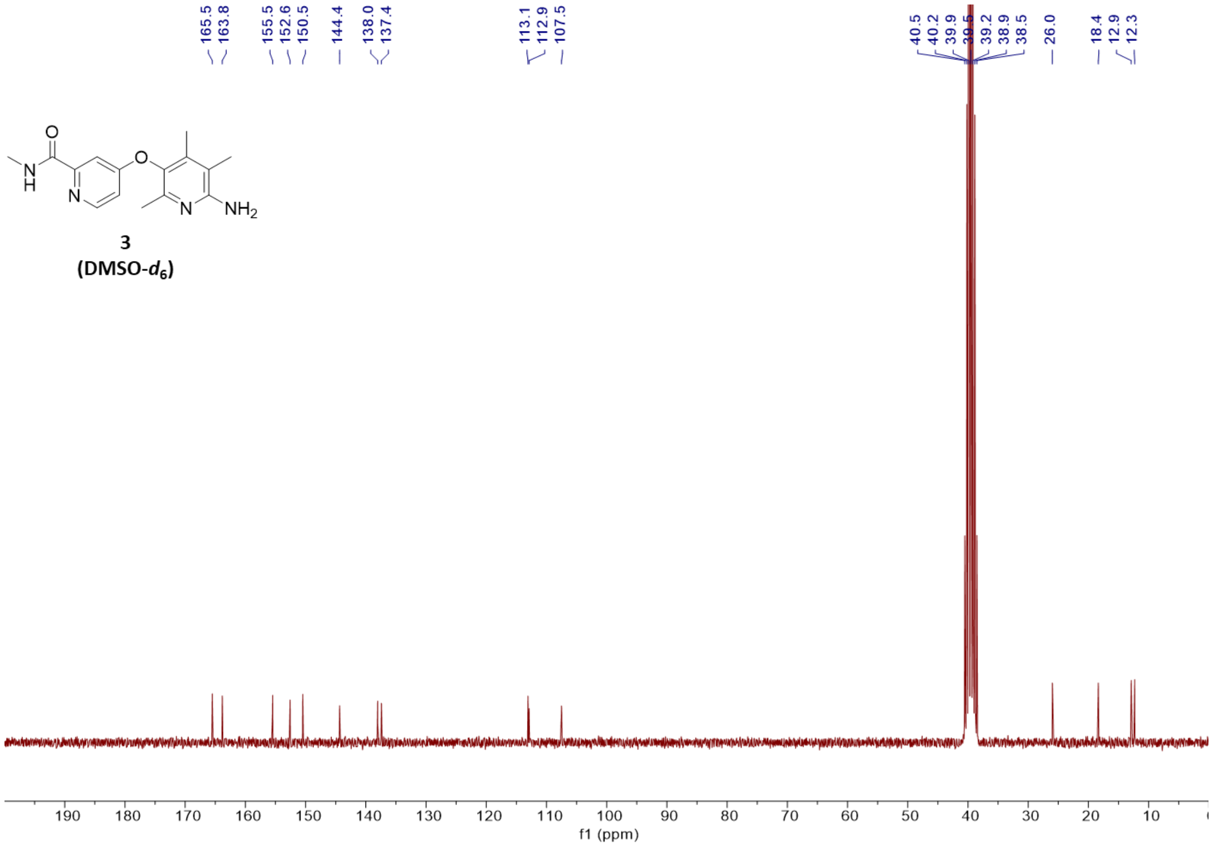
165.5
163.8
155.5
152.6
150.5
144.4
138.0
137.4
113.1
112.9
107.5
40.5
40.2
39.9
39.5
39.2
38.9
38.5
26.0
18.4
12.9
12.3
O
N
H
N
O
N
NH_2
3
(DMSO-d_6)
f1 (ppm)

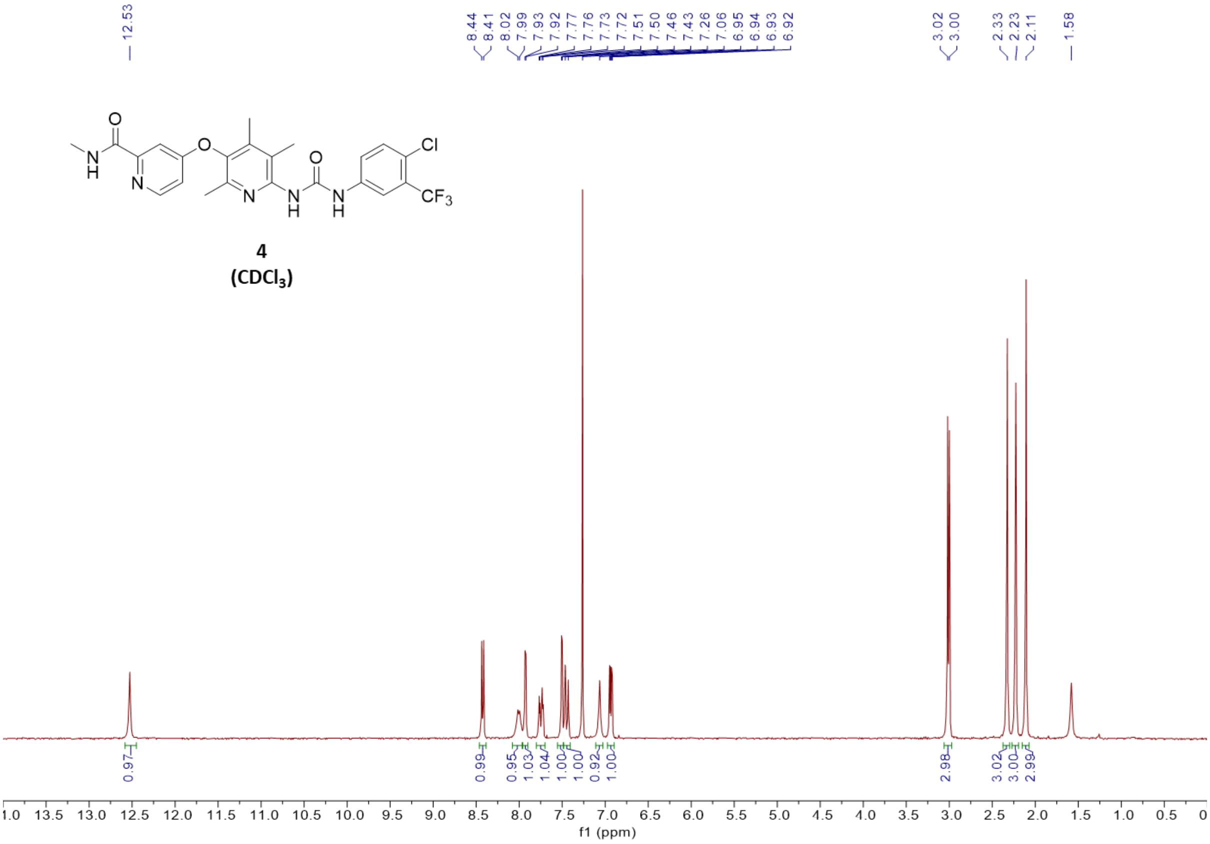
12.53
8.44 8.41 8.02 7.99 7.93 7.92 7.77 7.76 7.73 7.72 7.51 7.50 7.46 7.43 7.26 7.06 6.95 6.94 6.93 6.92
3.02 3.00
2.33 2.23 2.11
1.58
4
($CDCl_3$)
0.97 0.99 0.95 1.03 1.04 1.00 1.00 0.92 1.00 2.98 3.02 3.00 2.99
f1 (ppm)

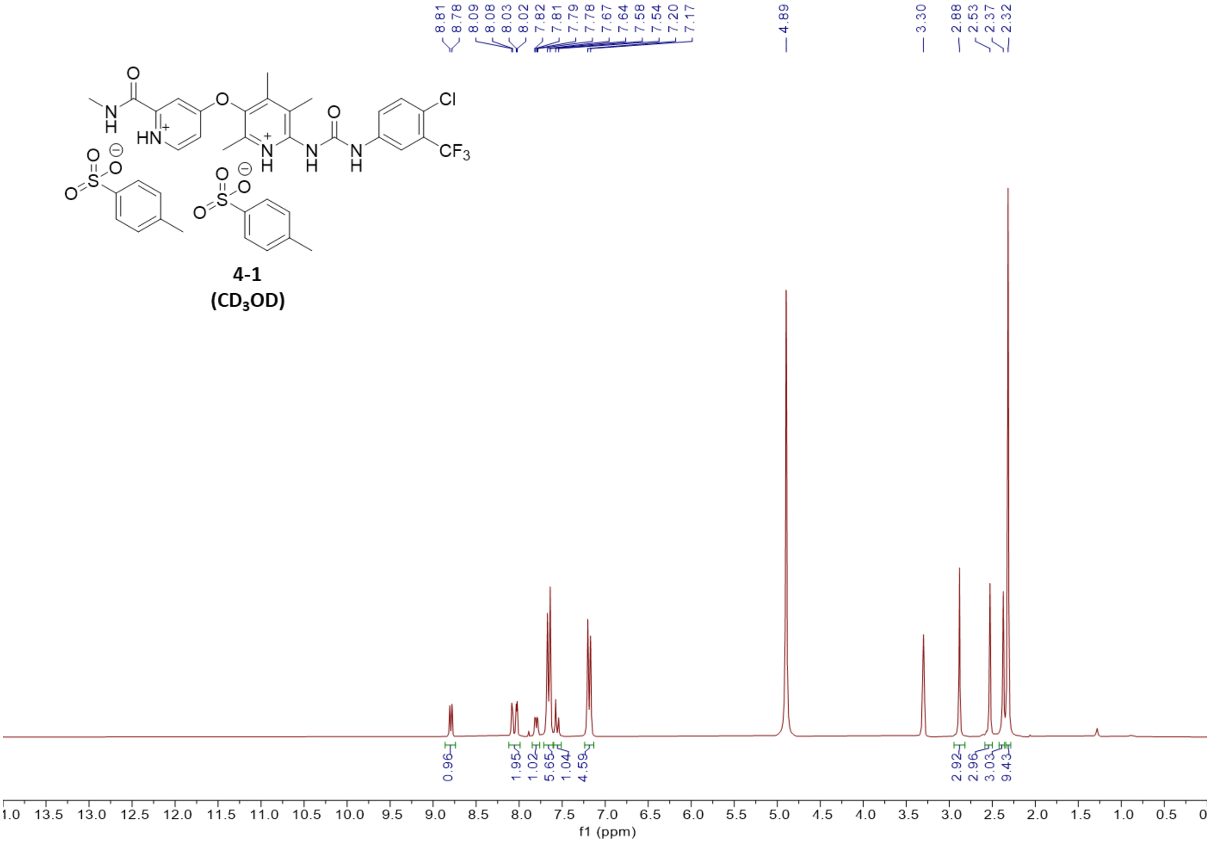
8.81 8.78 8.09 8.08 8.03 8.02 7.82 7.81 7.79 7.78 7.67 7.64 7.58 7.54 7.20 7.17
4.89
3.30
2.88 2.53 2.37 2.32
4-1
(CD_3OD)
0.96 1.95 1.02 5.65 1.04 4.59 2.92 2.96 3.03 9.43
f1 (ppm)

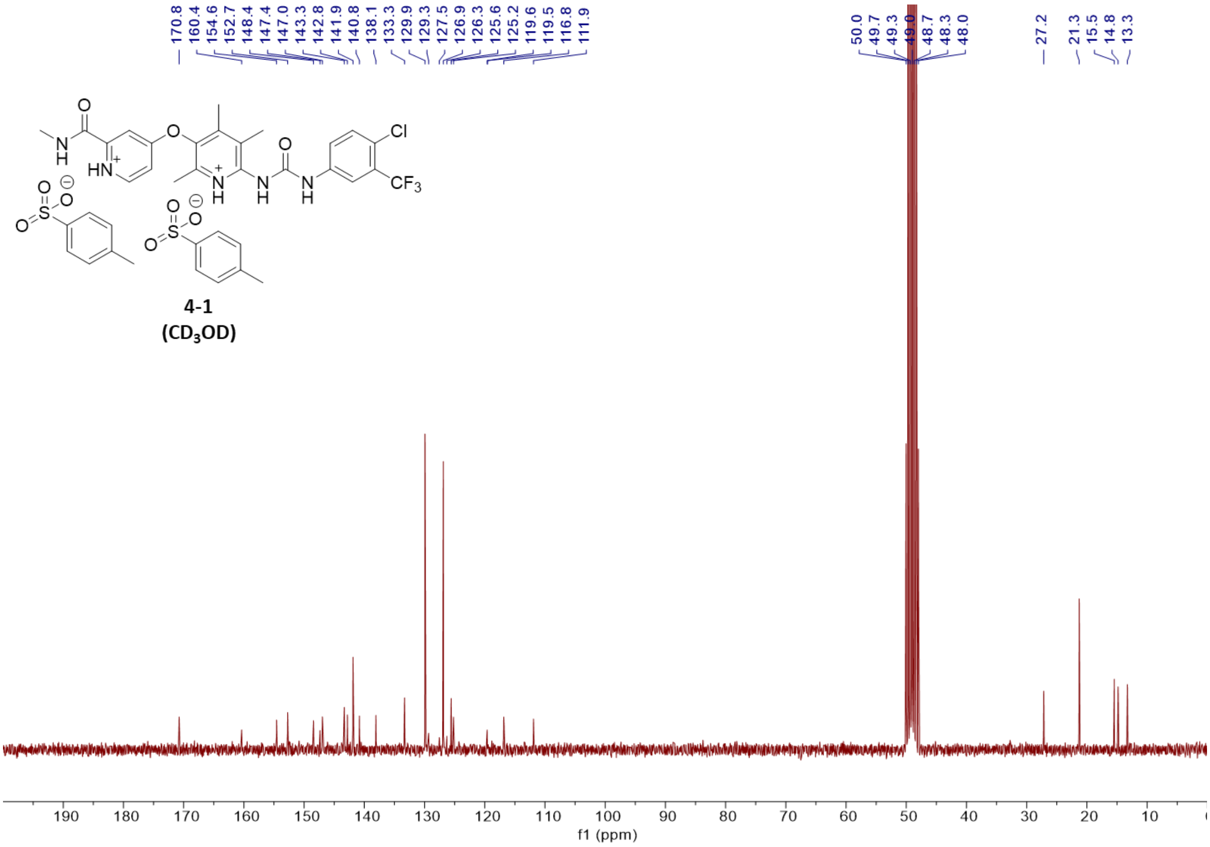
170.8
160.4
154.6
152.7
148.4
147.4
147.0
143.3
142.8
141.9
140.8
138.1
133.3
129.9
129.3
127.5
126.9
126.3
125.6
125.2
119.6
119.5
116.8
111.9
50.0
49.7
49.3
49.0
48.7
48.3
48.0
27.2
21.3
15.5
14.8
13.3
4-1
(CD_3OD)
f1 (ppm)

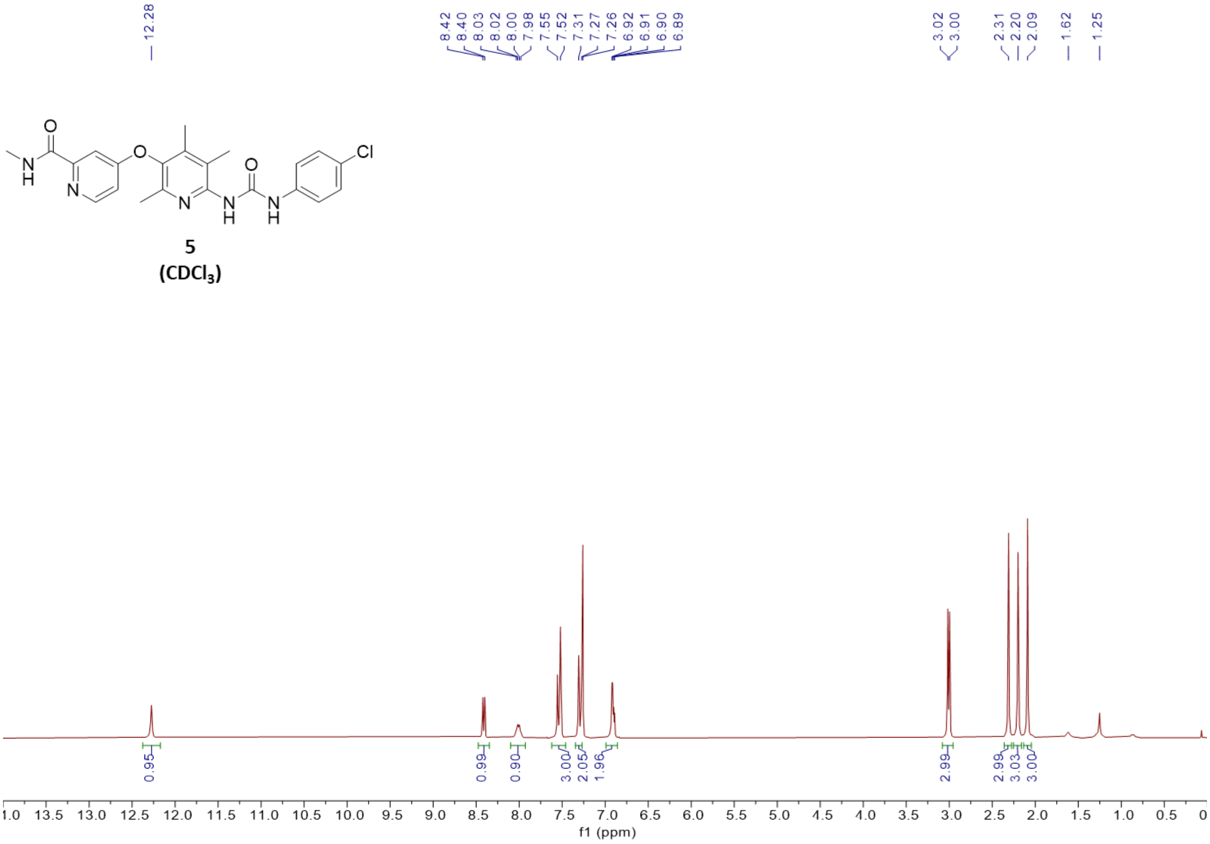
12.28
8.42
8.40
8.03
8.02
8.00
7.98
7.55
7.52
7.31
7.27
7.26
6.92
6.91
6.90
6.89
3.02
3.00
2.31
2.20
2.09
1.62
1.25
5
($CDCl_3$)
0.95
0.99
0.90
3.00
2.05
1.96
2.99
2.99
3.03
3.00
f1 (ppm)

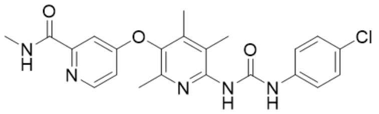

5
($CDCl_3$)

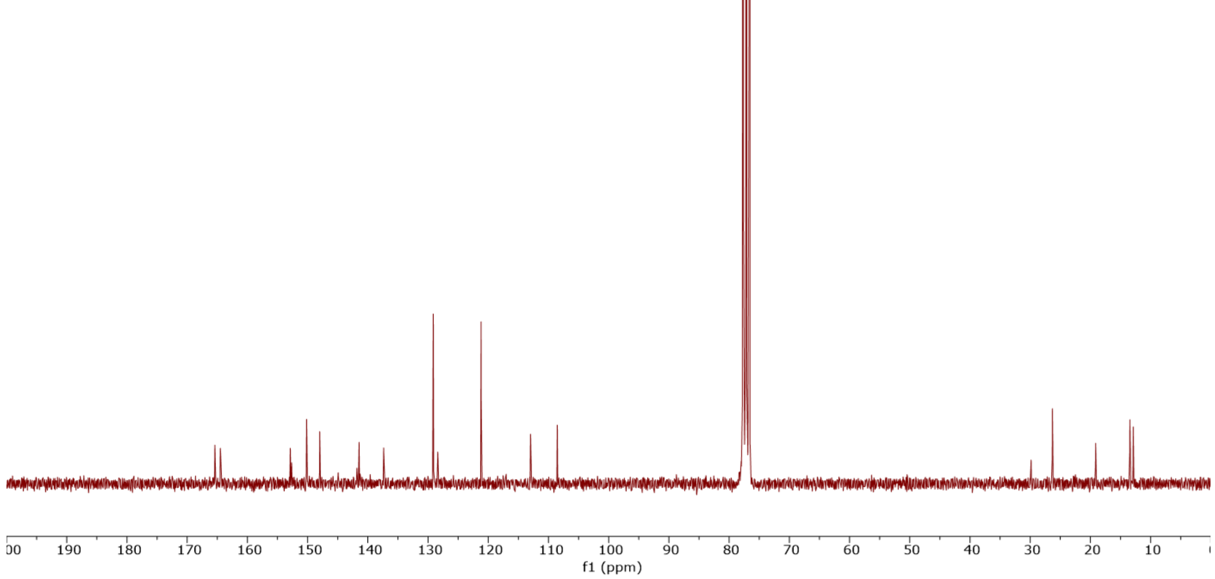

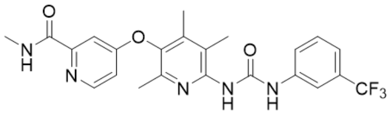

6
($CDCl_3$)

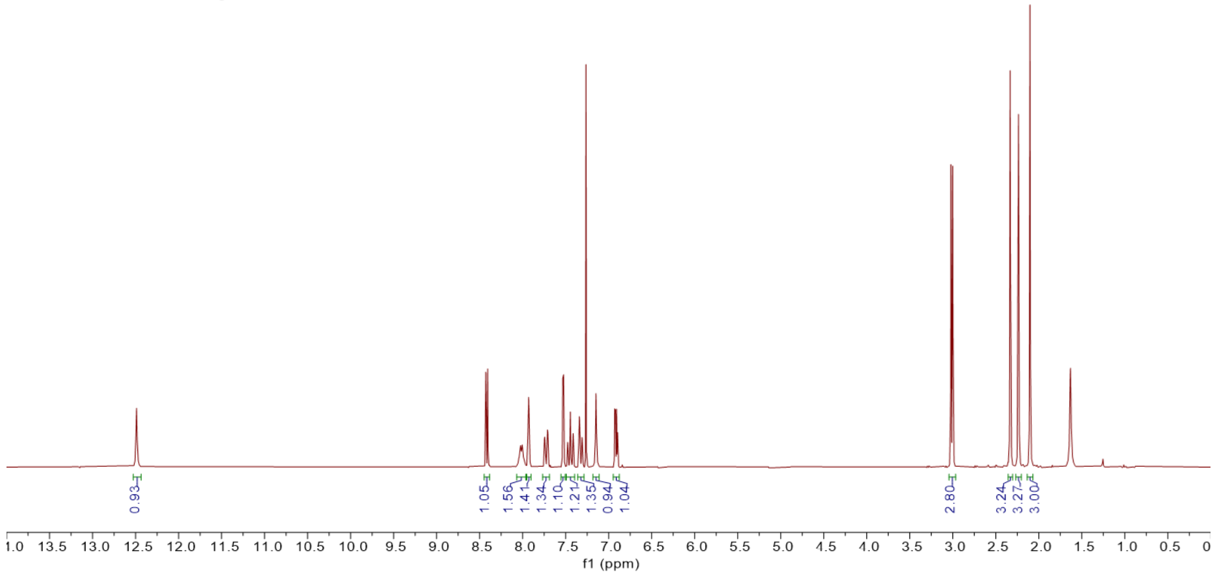

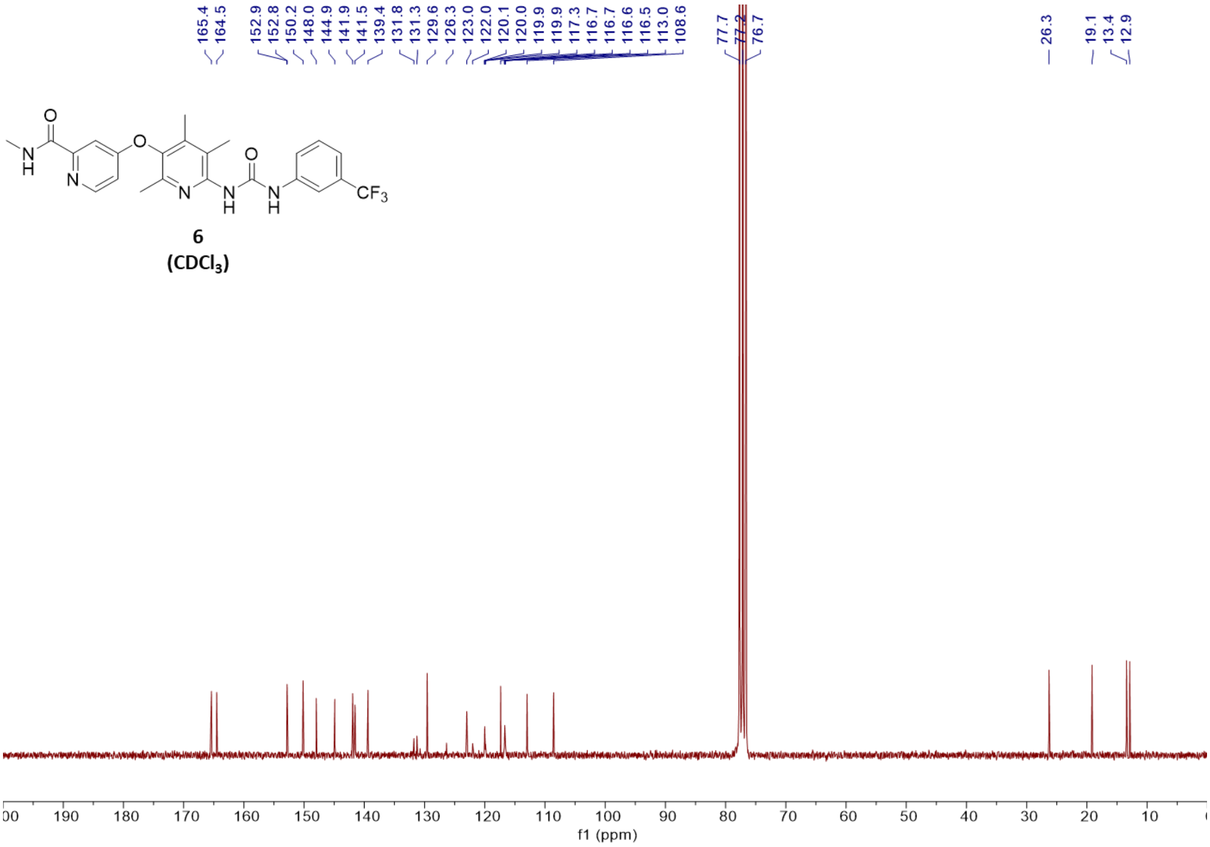

6
($CDCl_3$)

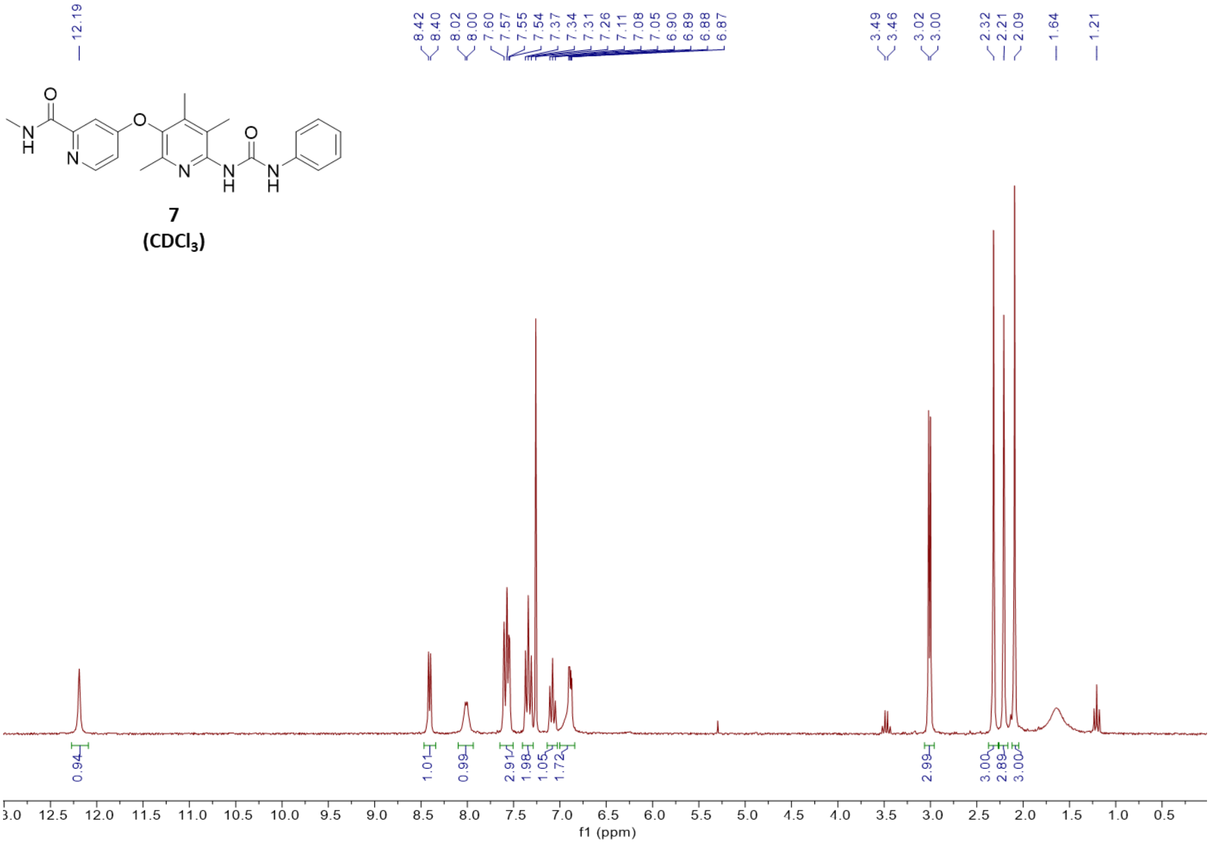

7
($CDCl_3$)

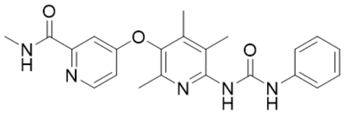

7
($CDCl_3$)

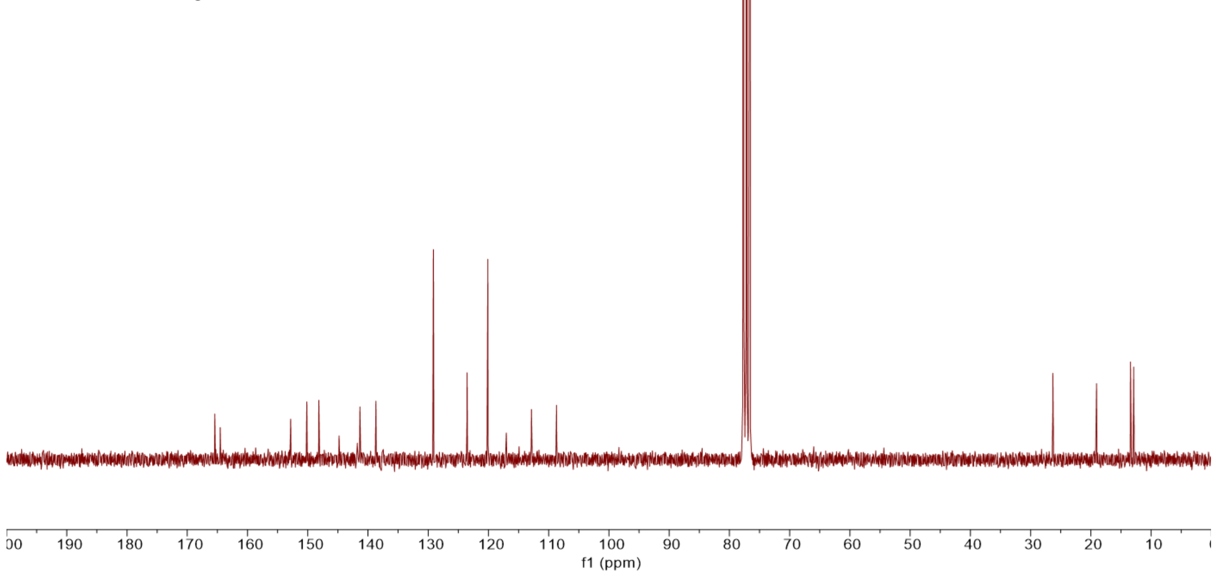

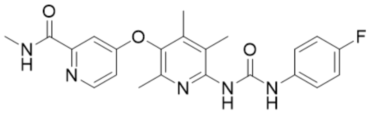

8
($CDCl_3$)

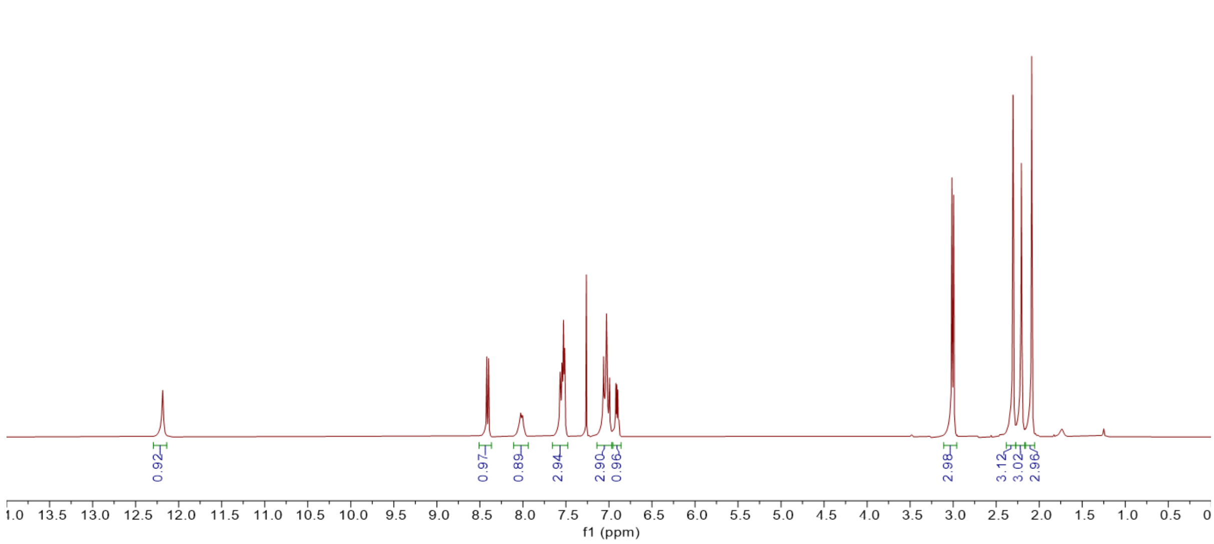

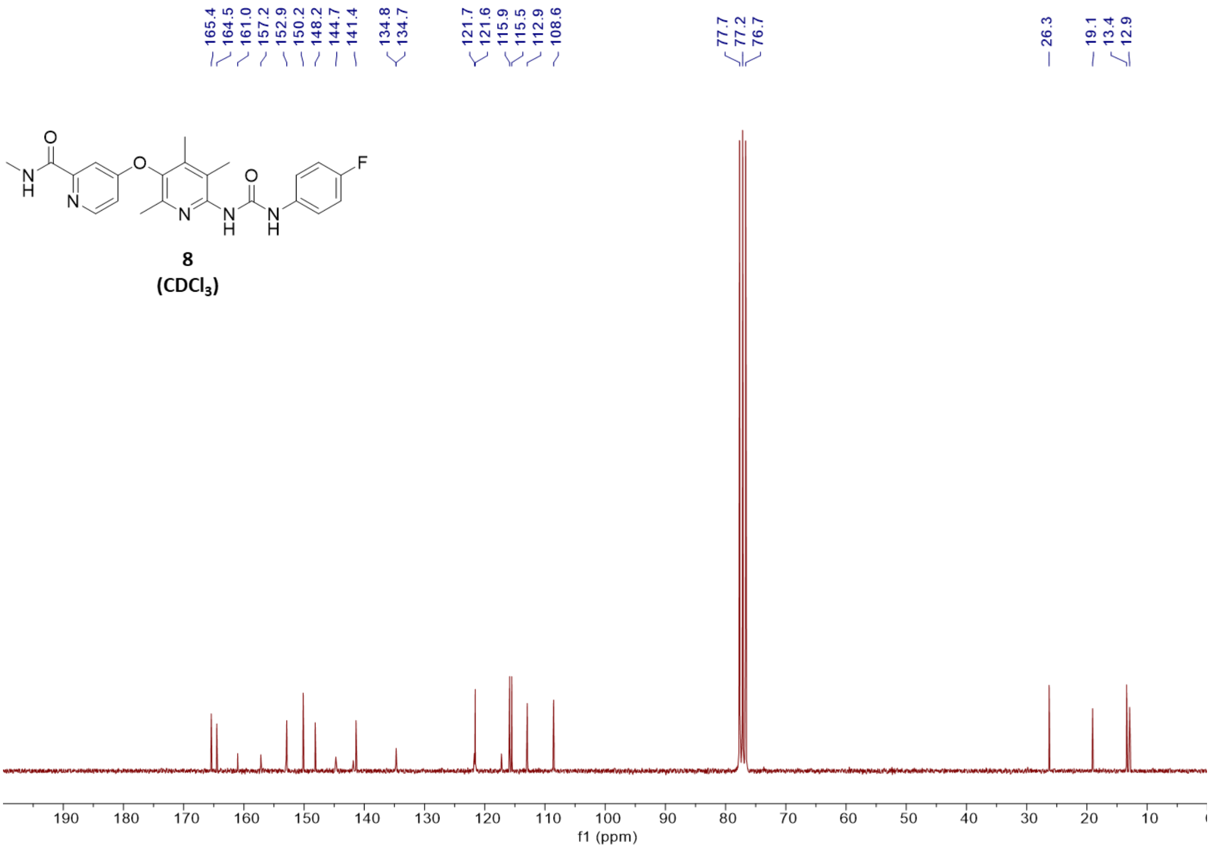

8
($CDCl_3$)

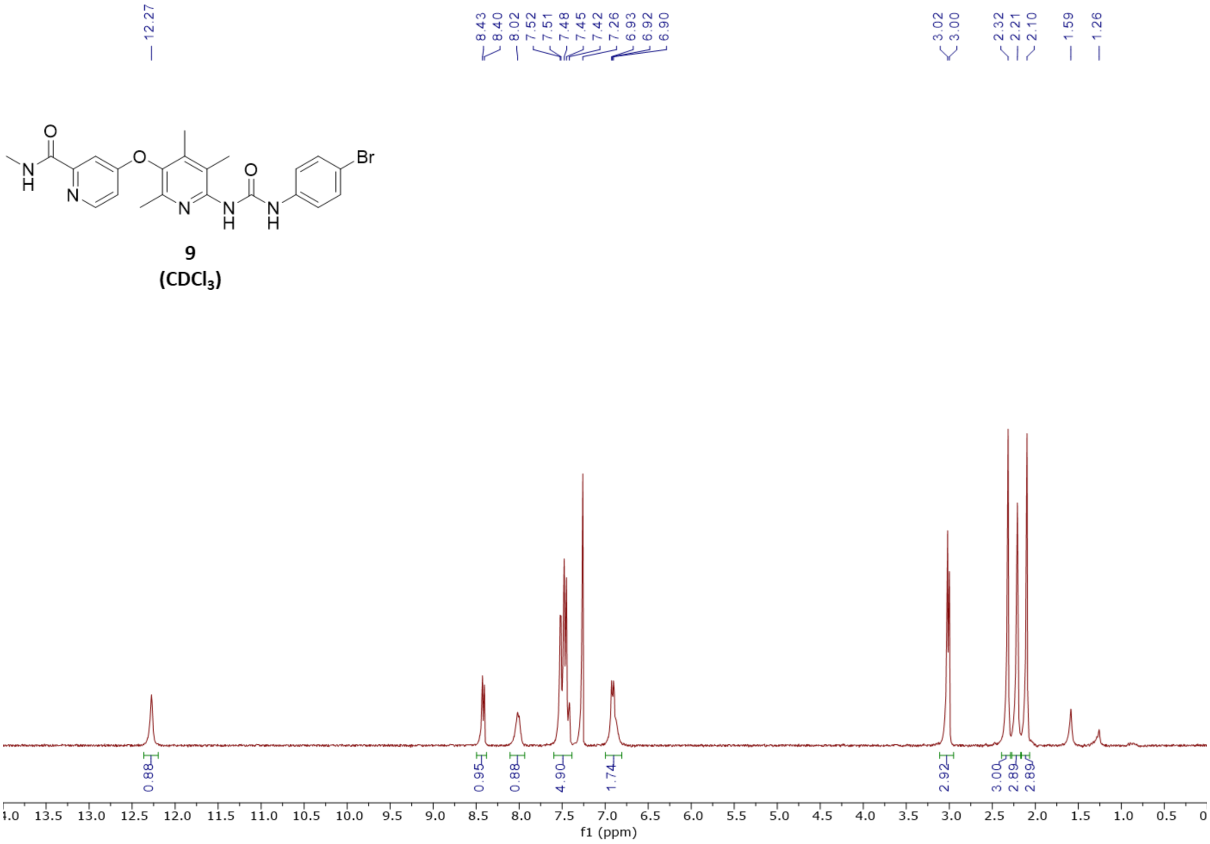

9
($CDCl_3$)

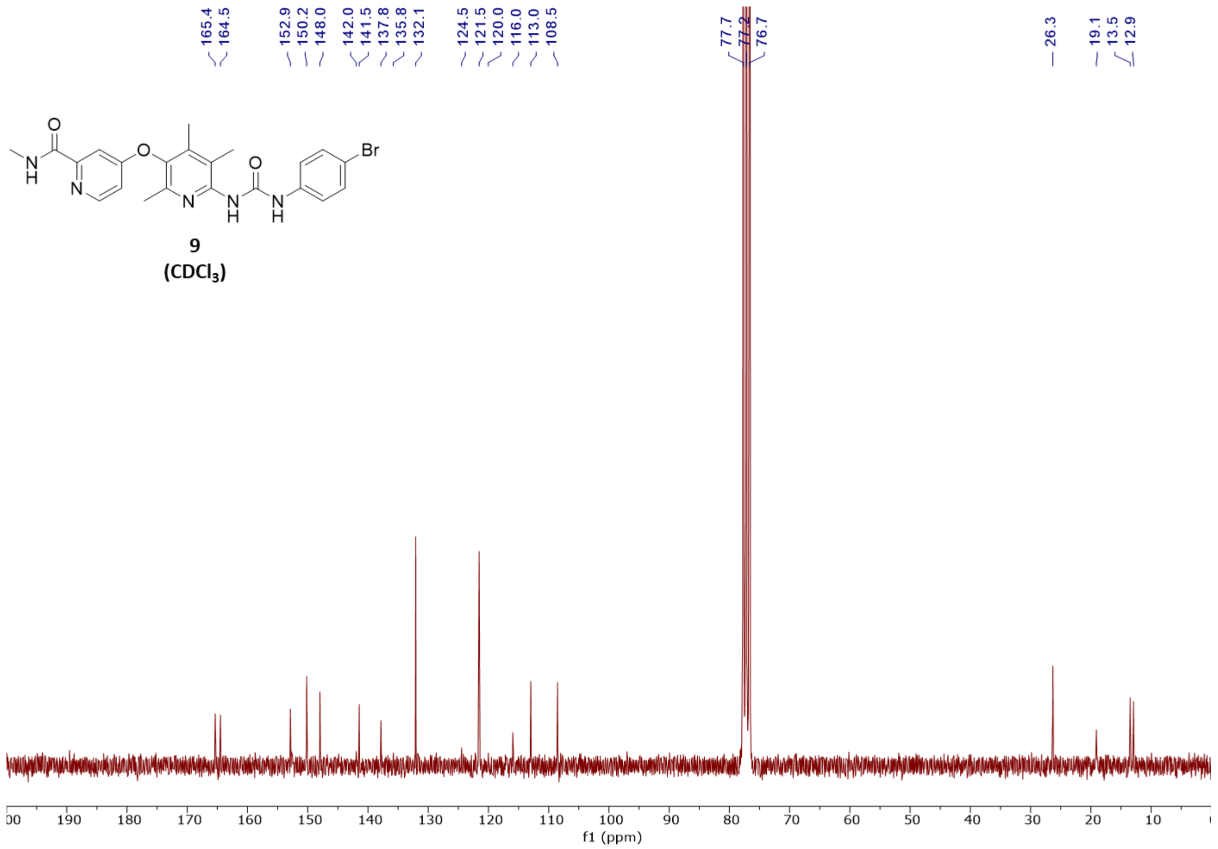

9
($CDCl_3$)

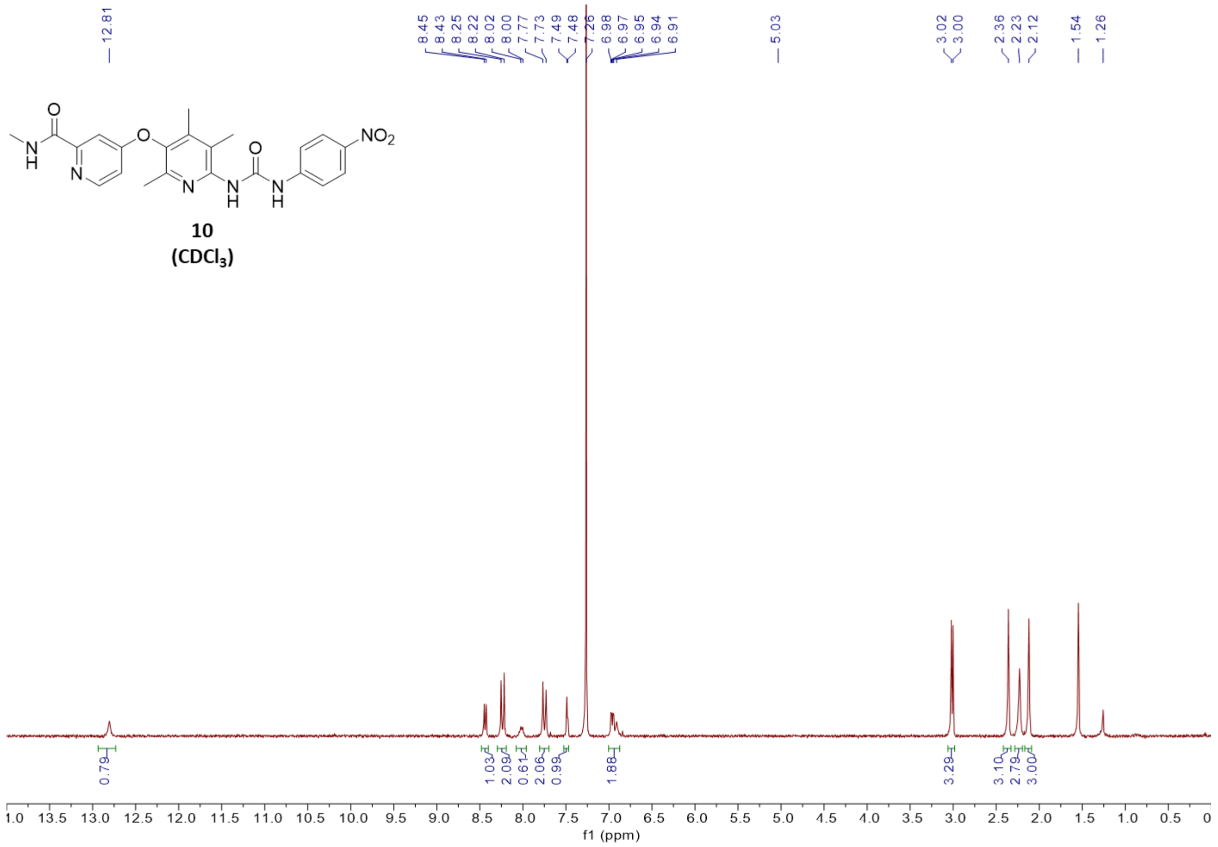

10
($CDCl_3$)

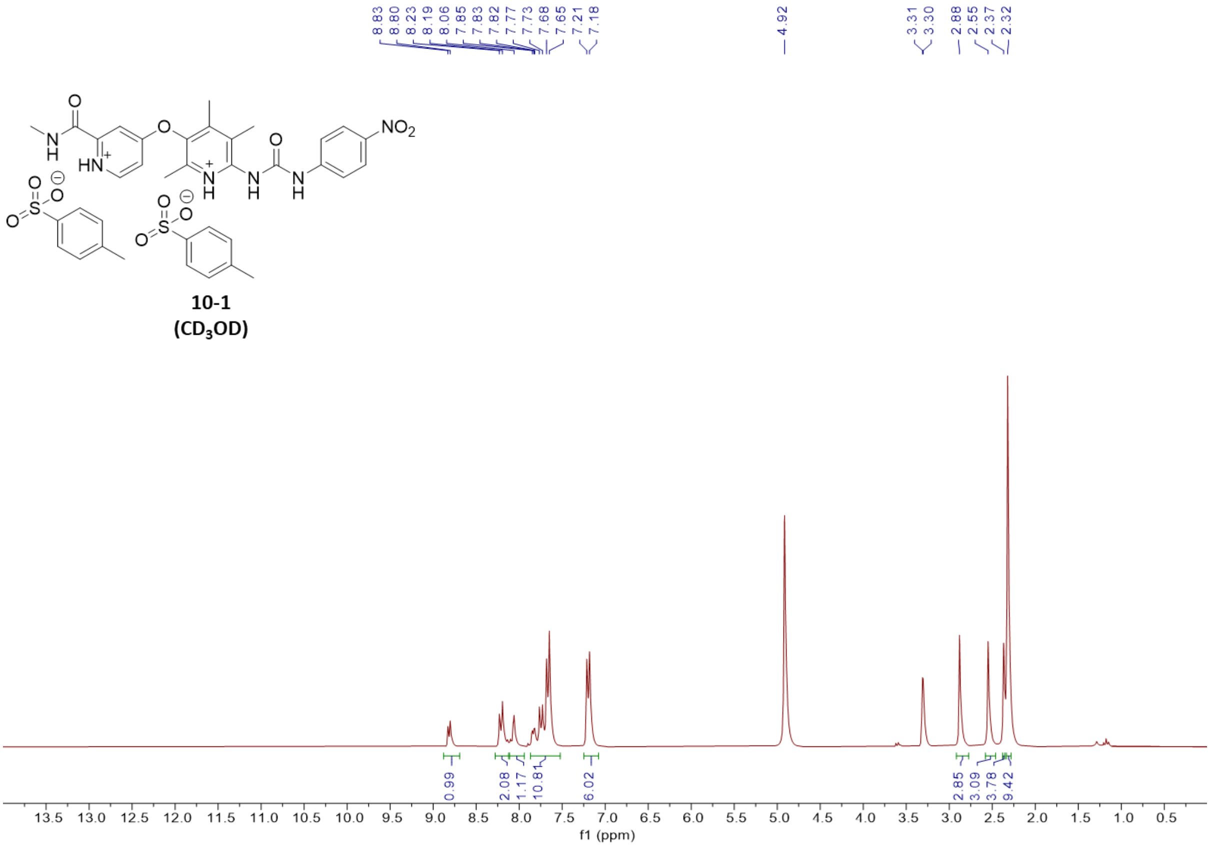
8.83
8.80
8.23
8.19
8.06
7.85
7.83
7.82
7.77
7.73
7.68
7.65
7.21
7.18
4.92
3.31
3.30
2.88
2.55
2.37
2.32
NO2
O
N
H
HN+
10-1
(CD3OD)
0.99
2.08
1.17
10.81
6.02
2.85
3.09
3.78
9.42
f1 (ppm)

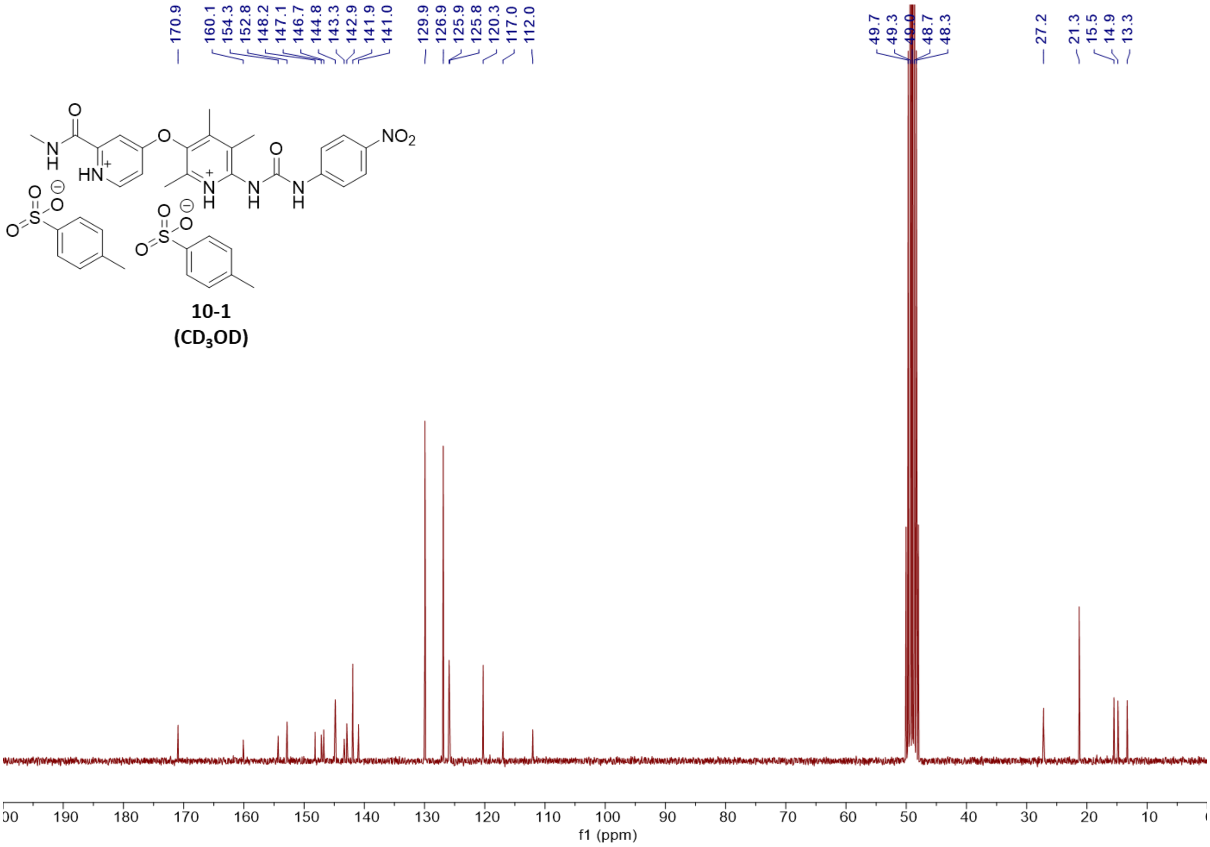
170.9
160.1
154.3
152.8
148.2
147.1
146.7
144.8
143.3
142.9
141.9
141.0
129.9
126.9
125.9
125.8
120.3
117.0
112.0
49.7
49.3
49.0
48.7
48.3
27.2
21.3
15.5
14.9
13.3
NO2
10-1
(CD3OD)
f1 (ppm)

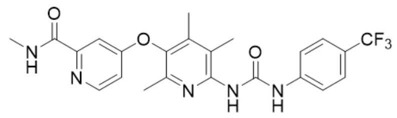

11
($CDCl_3$)

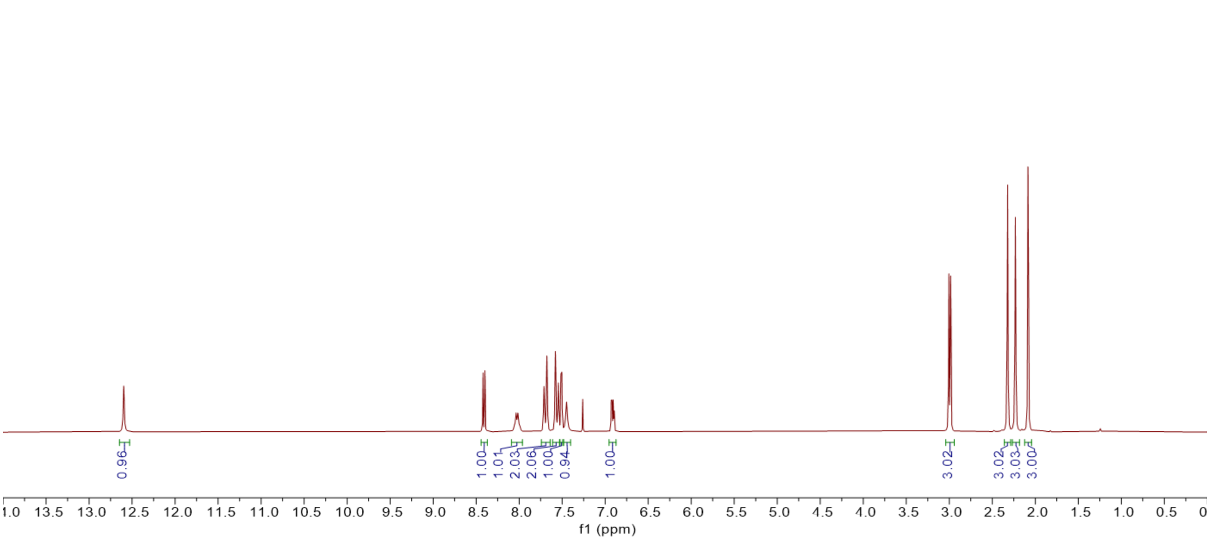

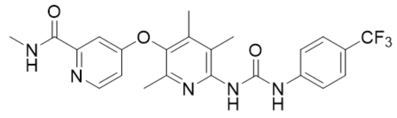

11
($CDCl_3$)

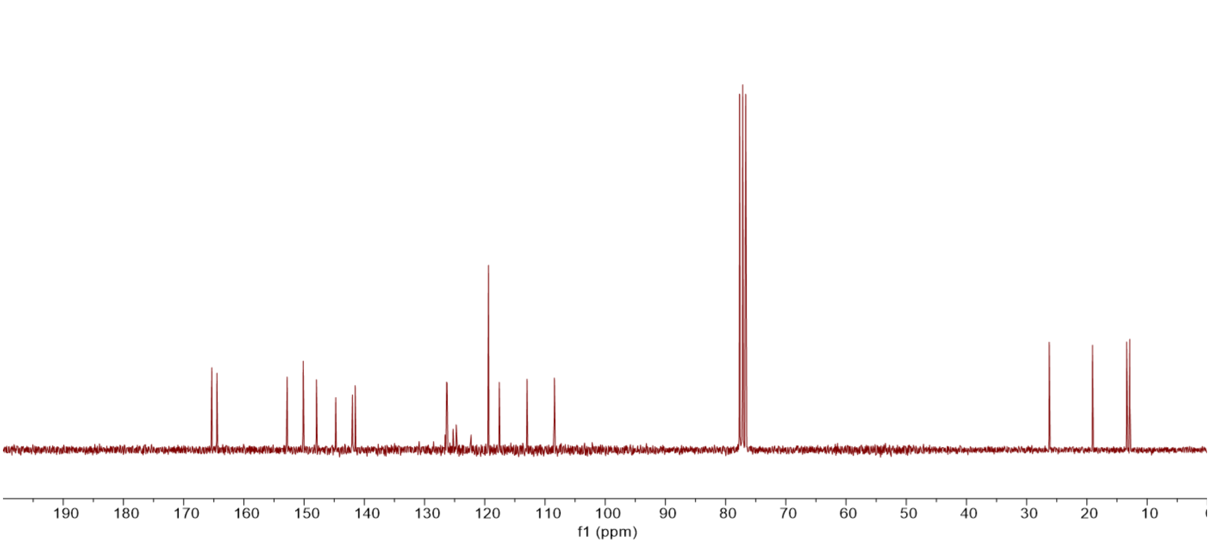

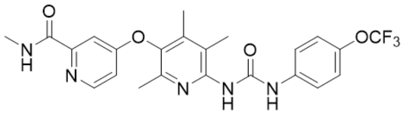

12
($CDCl_3$)

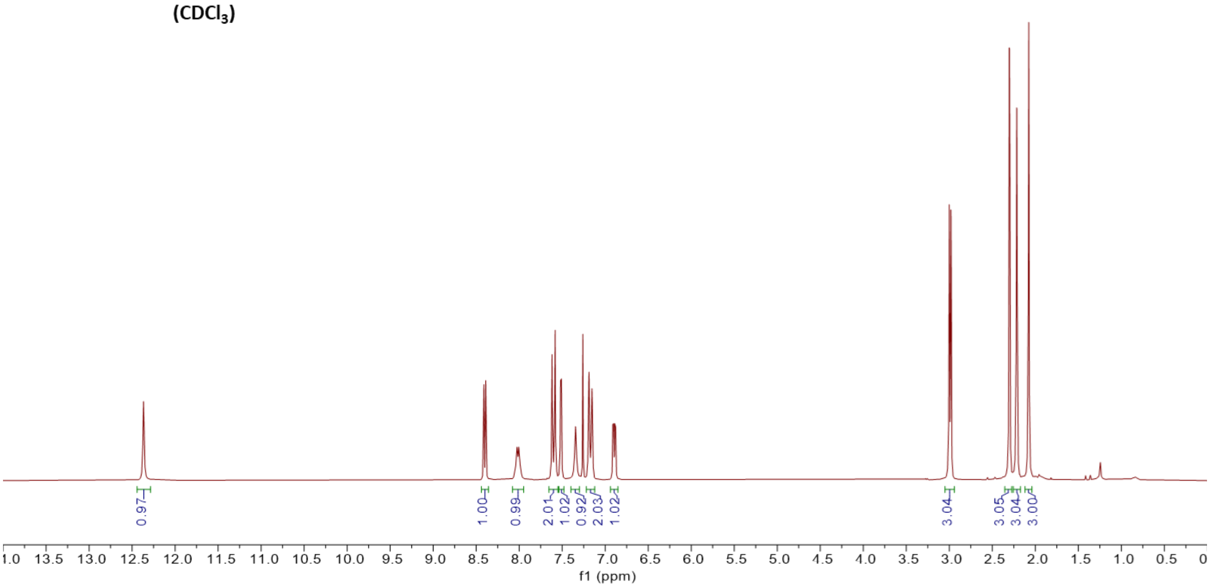

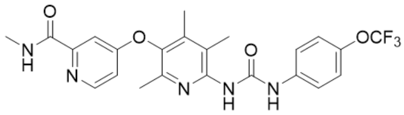

12
($CDCl_3$)

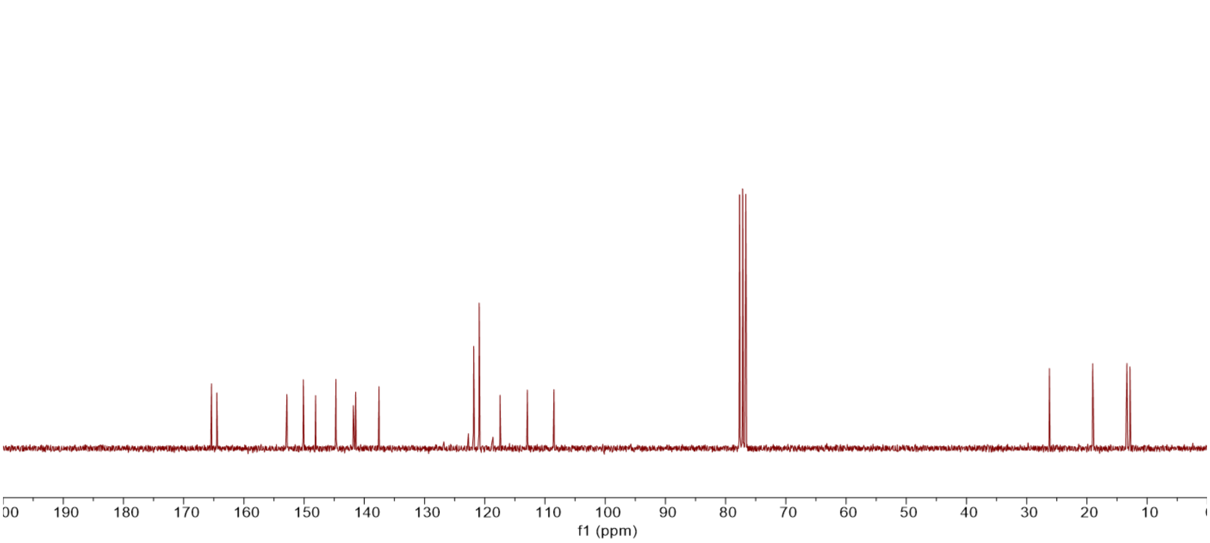

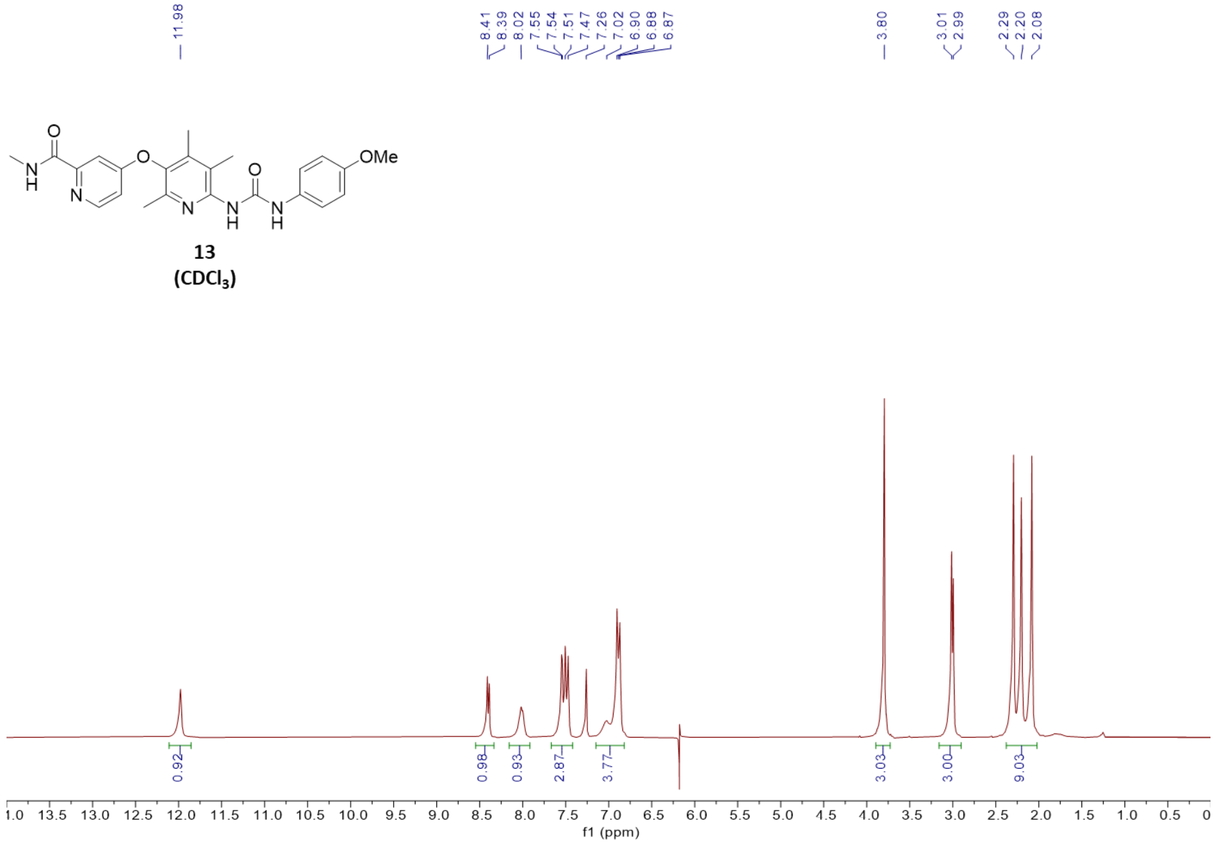
11.98
8.41 8.39 8.02 7.55 7.54 7.51 7.47 7.26 7.02 6.90 6.88 6.87
3.80
3.01 2.99
2.29 2.20 2.08
O
N
H
N
O
OMe
O
N
H
N
H
13
($CDCl_3$)
0.92 0.98 0.93 2.87 3.77 3.03 3.00 9.03
f1 (ppm)

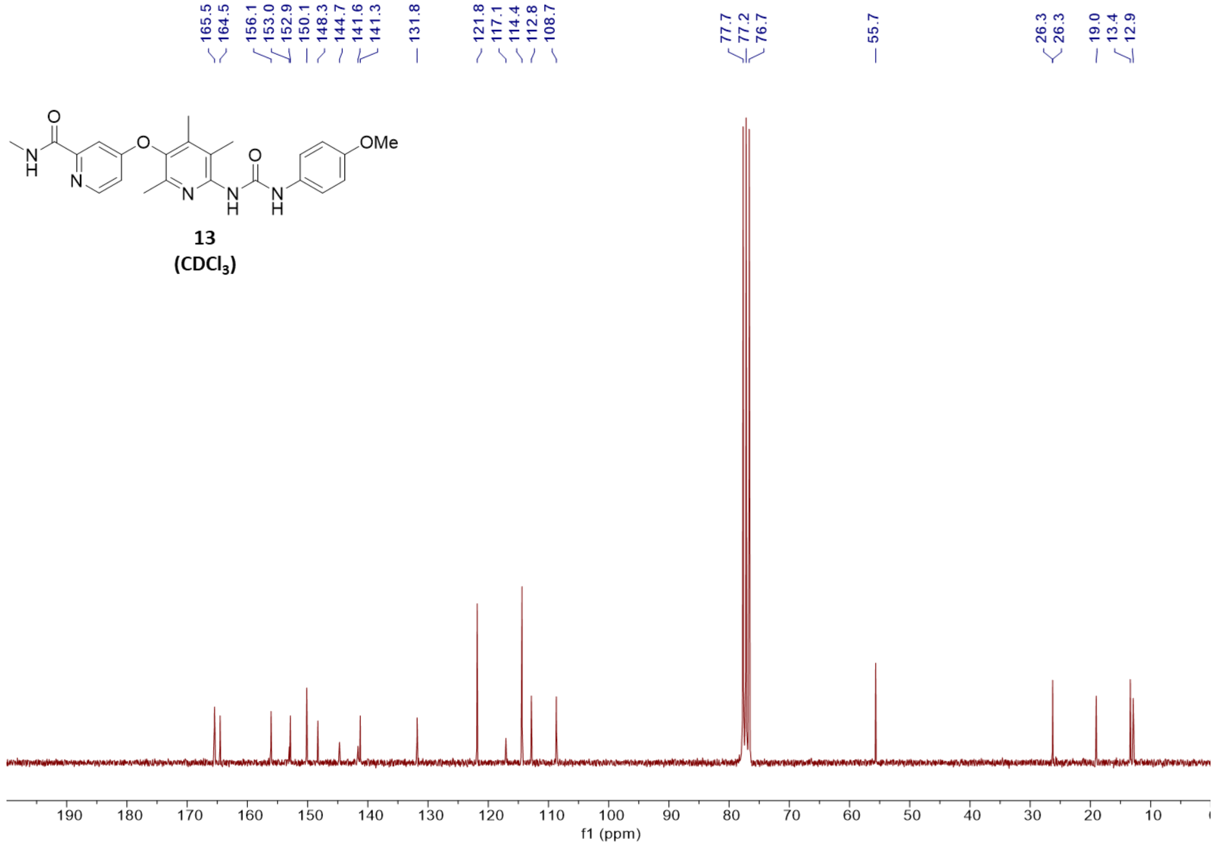
165.5 164.5 156.1 153.0 152.9 150.1 148.3 144.7 141.6 141.3 131.8 121.8 117.1 114.4 112.8 108.7 77.7 77.2 76.7 55.7 26.3 26.3 19.0 13.4 12.9
O
N
H
N
O
OMe
O
N
H
N
H
13
($CDCl_3$)
f1 (ppm)

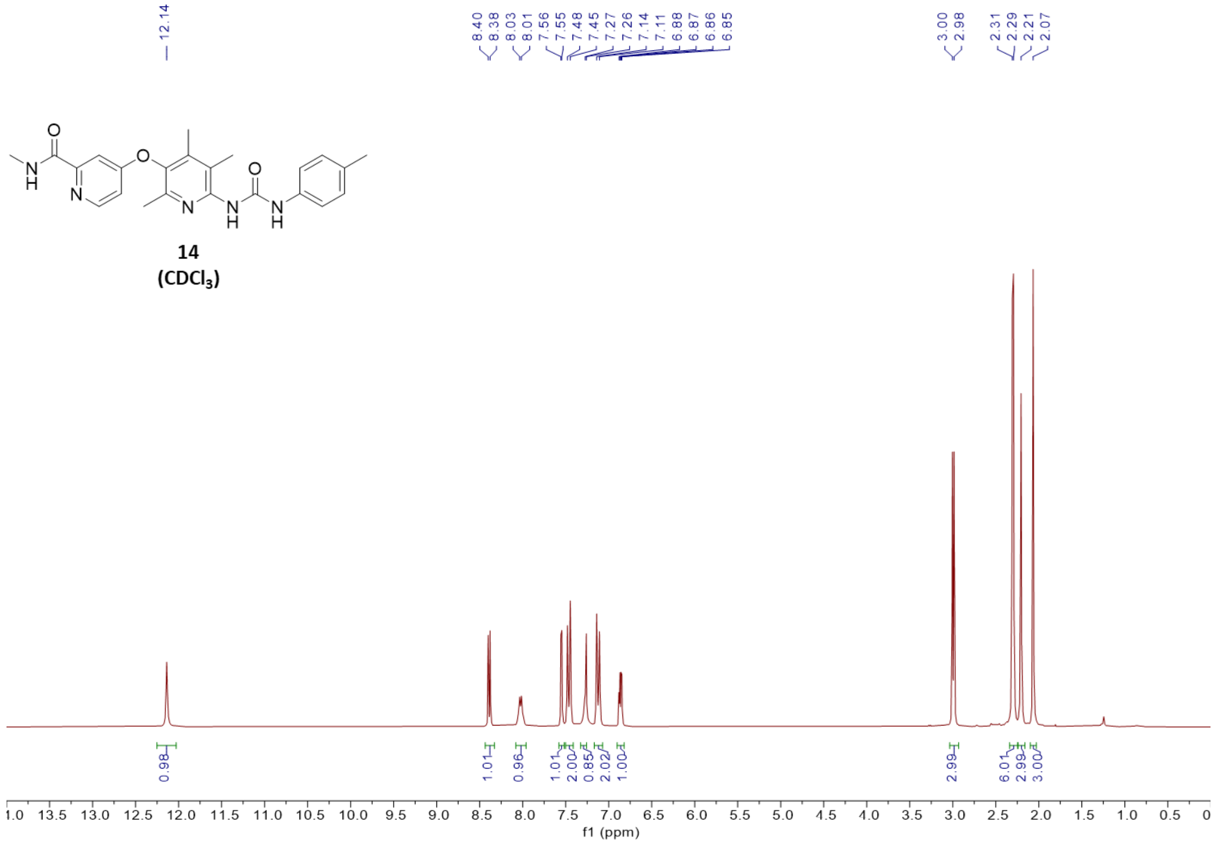

14
($CDCl_3$)

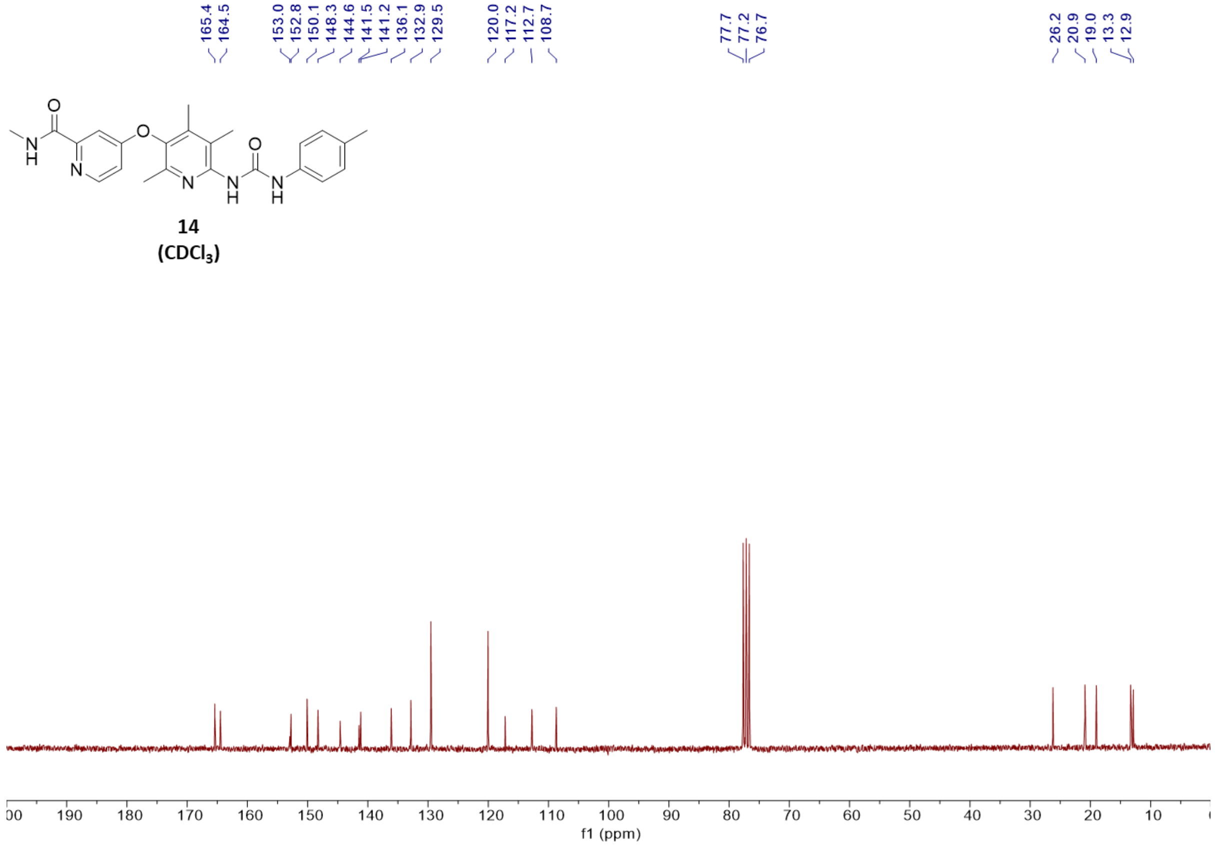

14
($CDCl_3$)

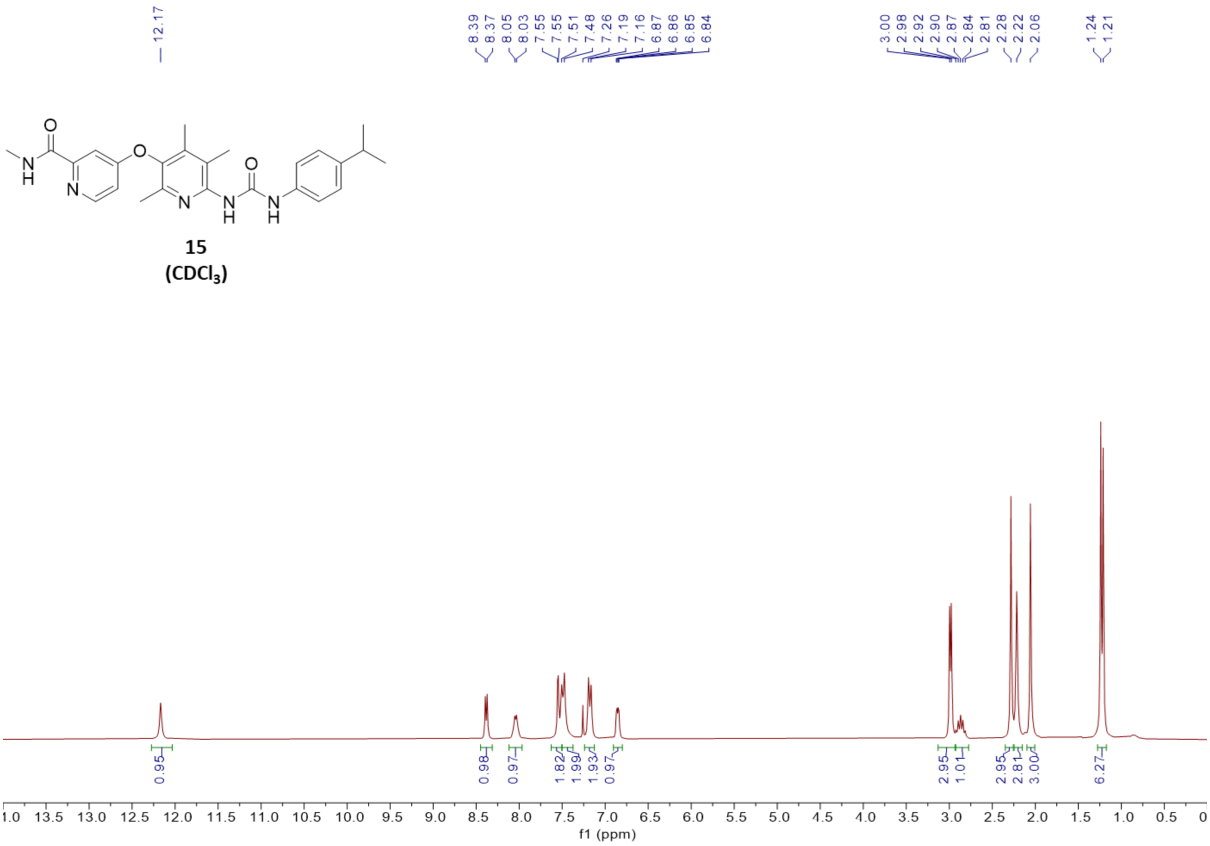

12.17
8.39
8.37
8.05
8.03
7.55
7.51
7.48
7.26
7.19
7.16
6.87
6.86
6.85
6.84
3.00
2.98
2.92
2.90
2.87
2.84
2.81
2.28
2.22
2.06
1.24
1.21
15
(CDCl3)
0.95
0.98
0.97
1.82
1.99
1.93
0.97
2.95
1.01
2.95
2.81
3.00
6.27
f1 (ppm)

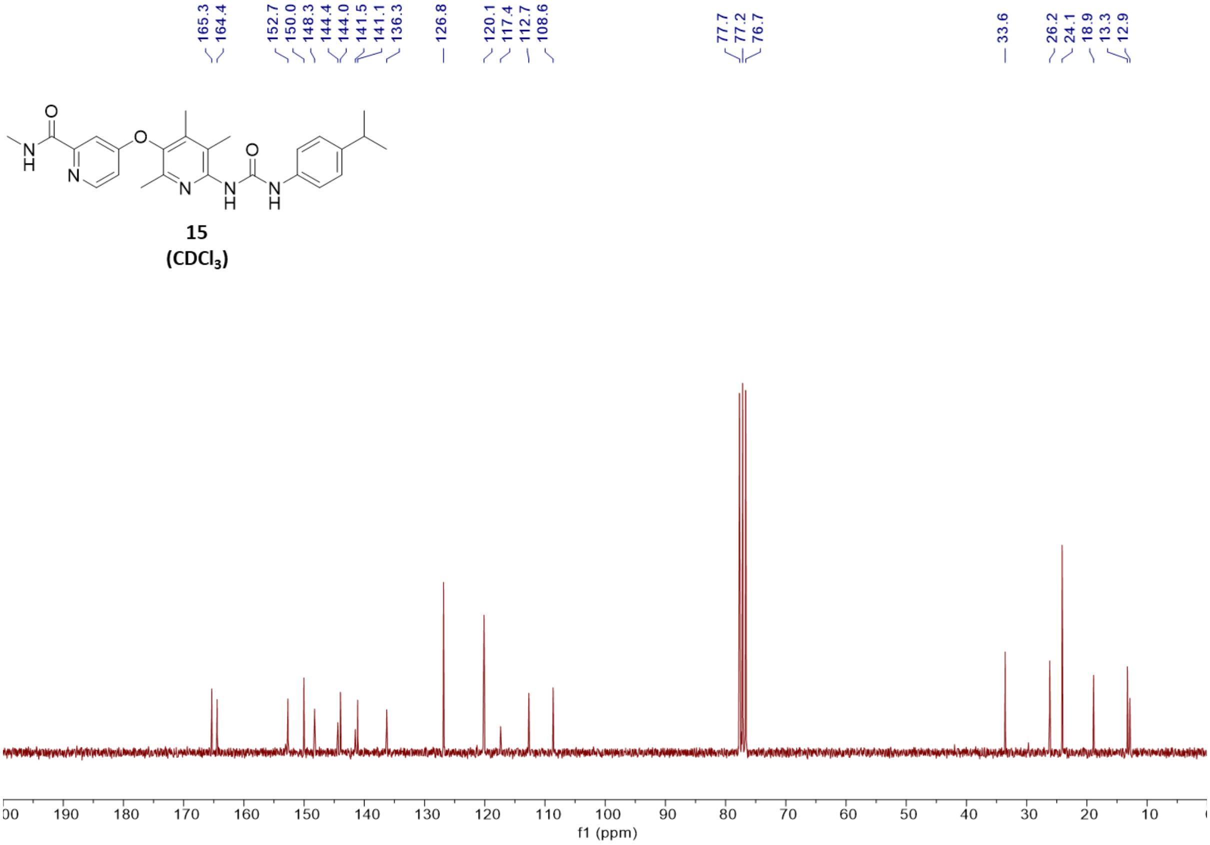

165.3
164.4
152.7
150.0
148.3
144.4
144.0
141.5
141.1
136.3
126.8
120.1
117.4
112.7
108.6
77.7
77.2
76.7
33.6
26.2
24.1
18.9
13.3
12.9
15
(CDCl3)
f1 (ppm)

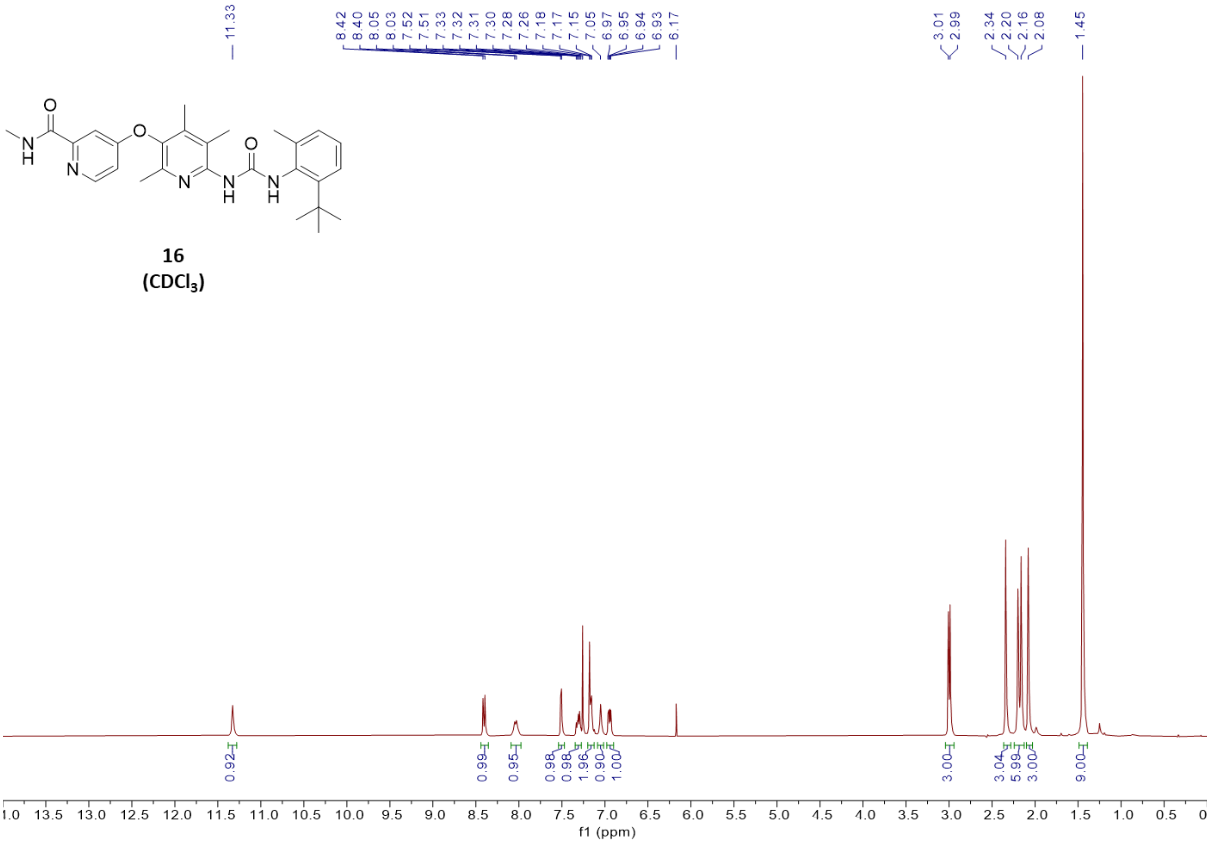

16
($CDCl_3$)

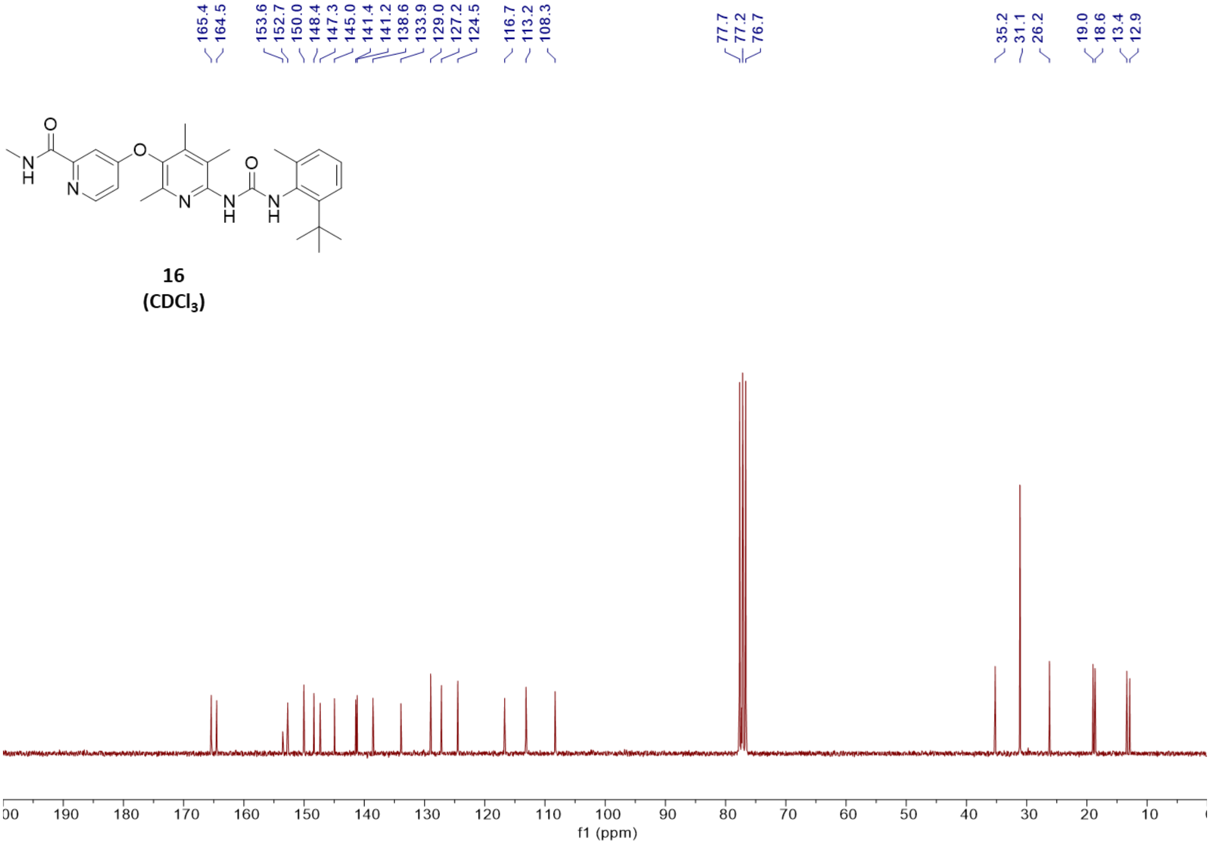

16
($CDCl_3$)

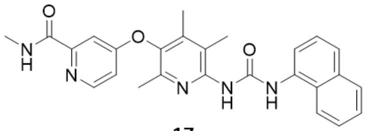

17
($CDCl_3$)

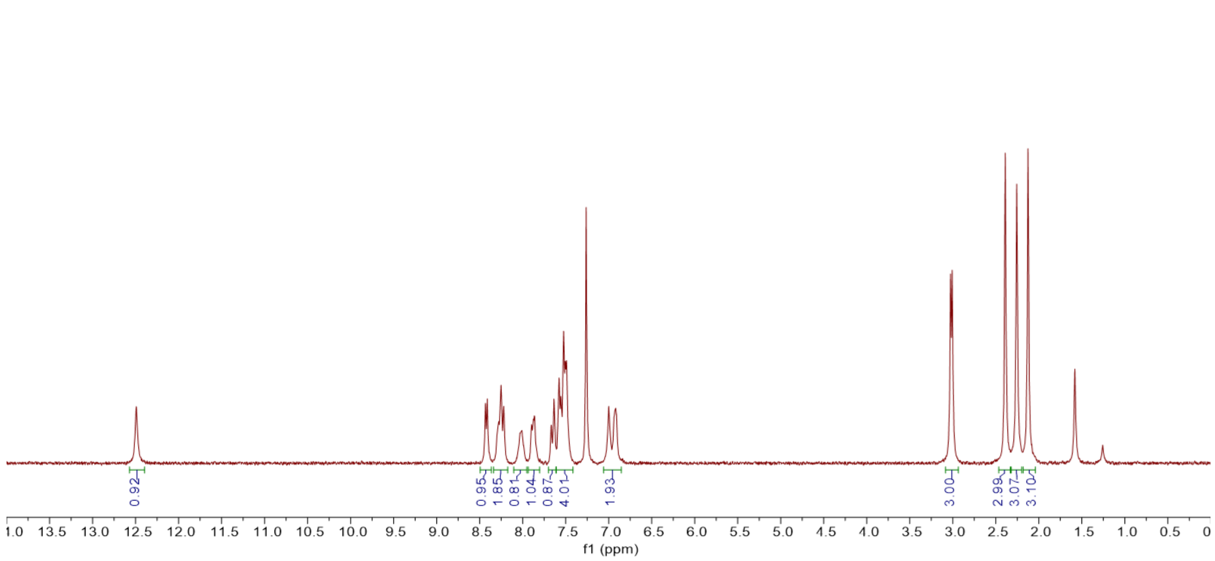

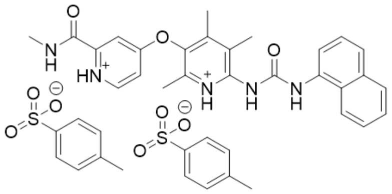

17-1
(CD_3OD)

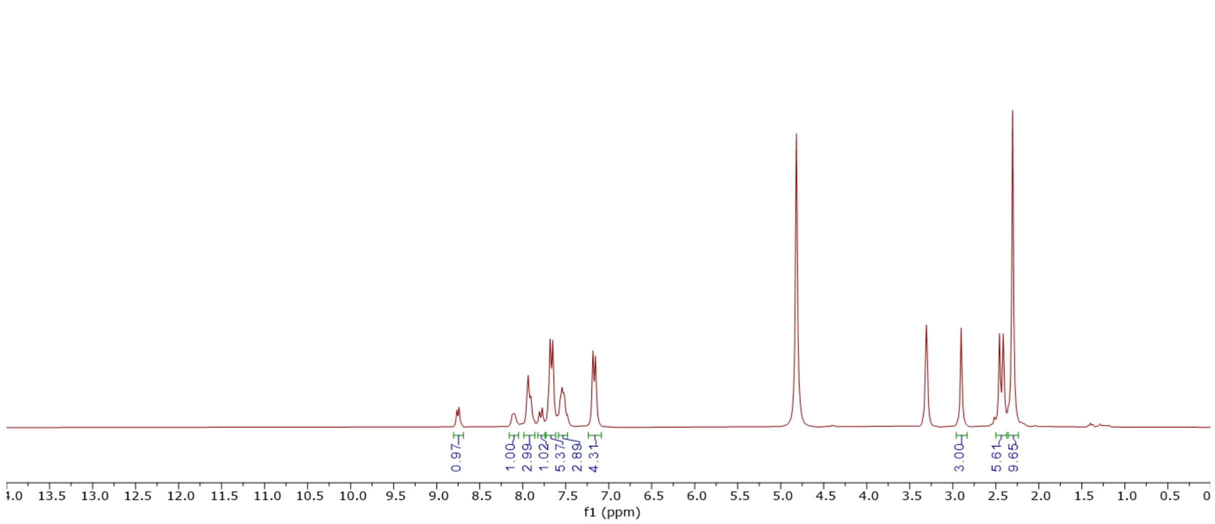

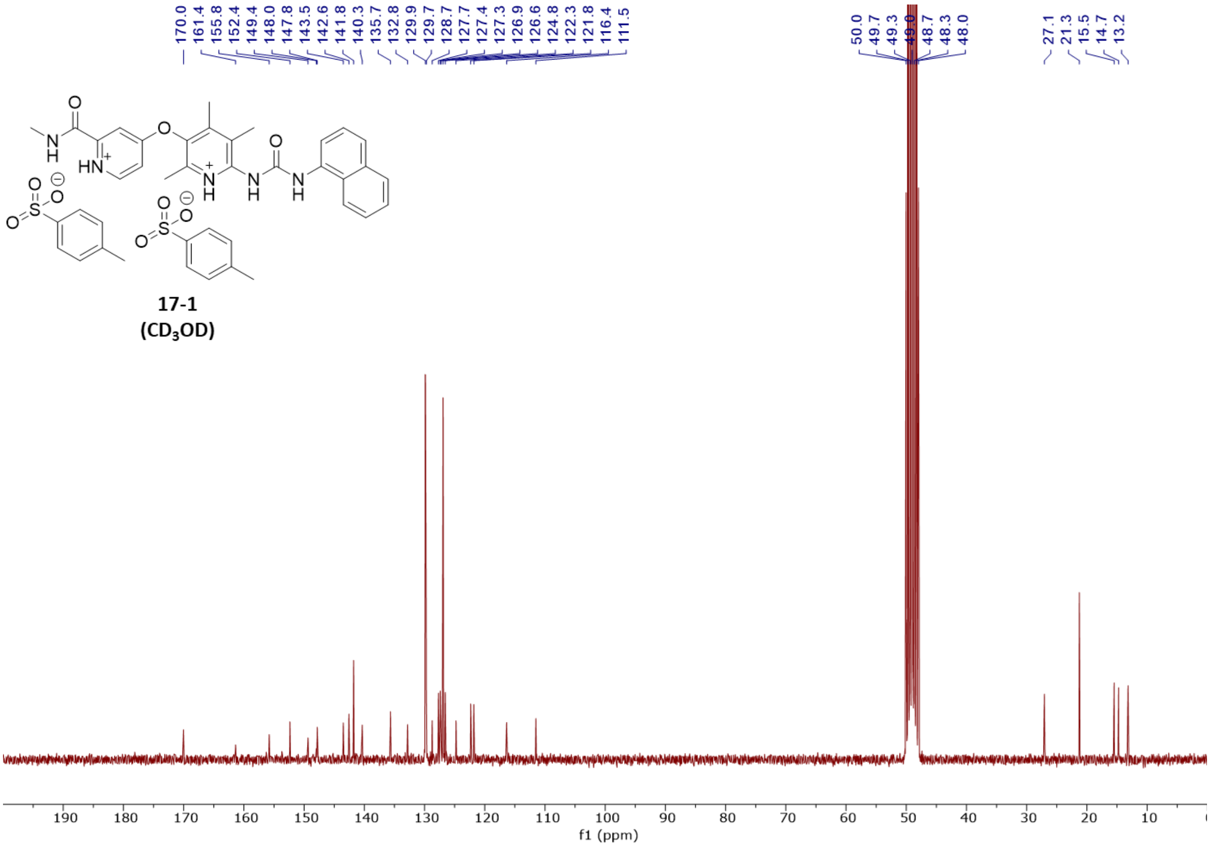

17-1
(CD_3OD)

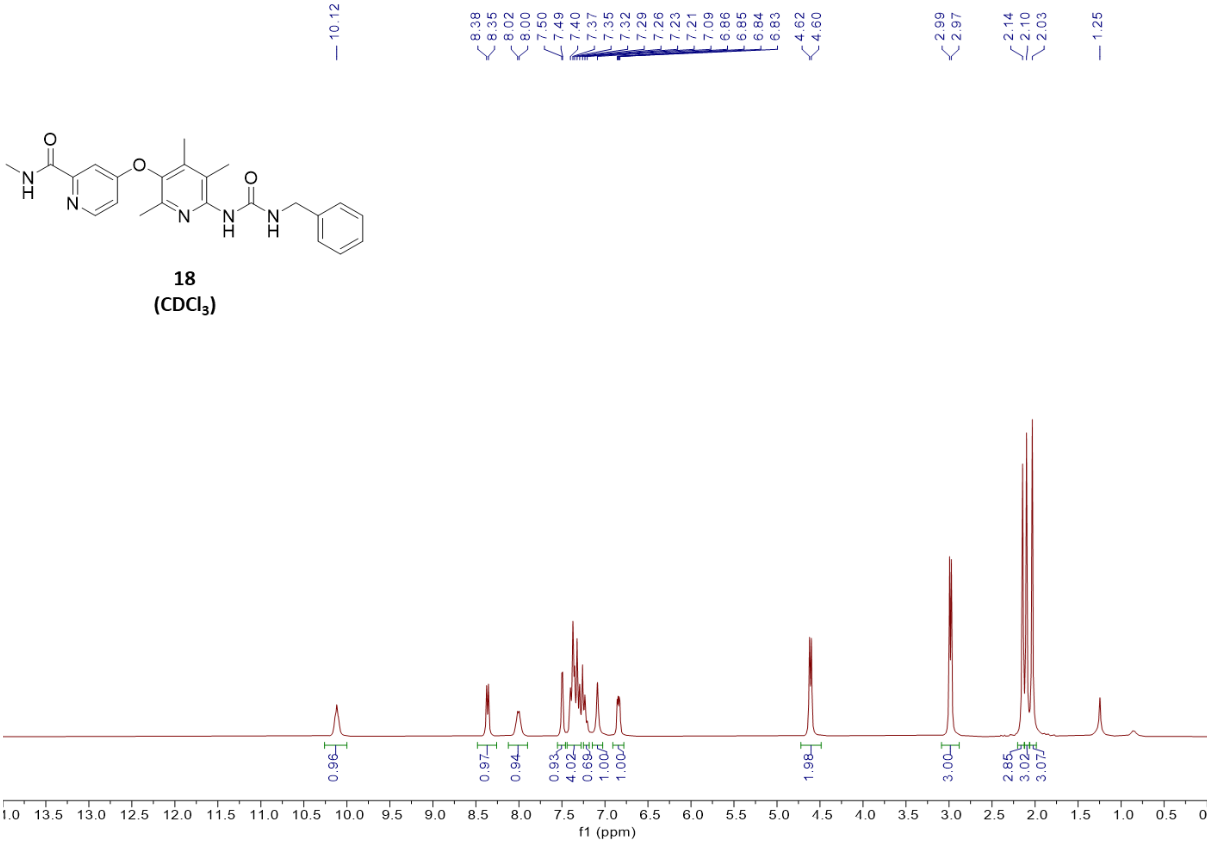

18
($CDCl_3$)

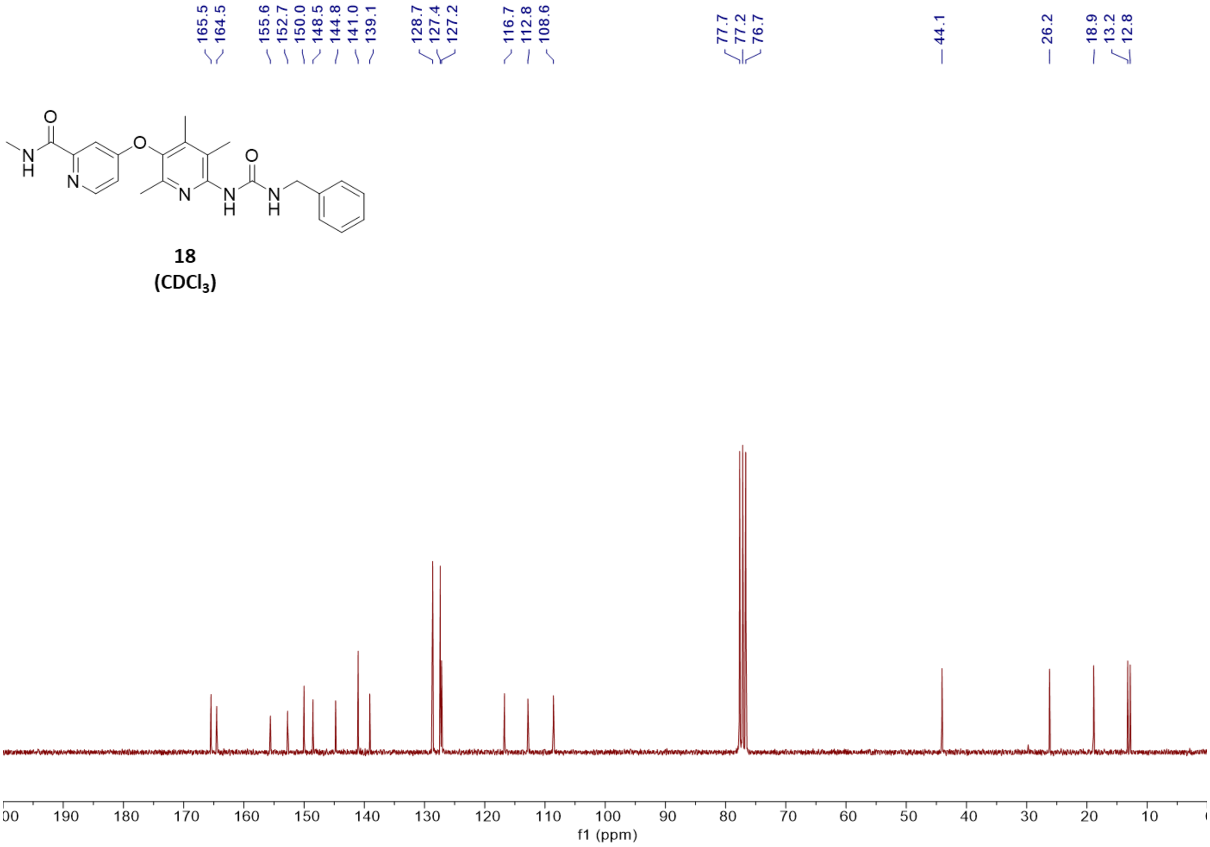

18
($CDCl_3$)

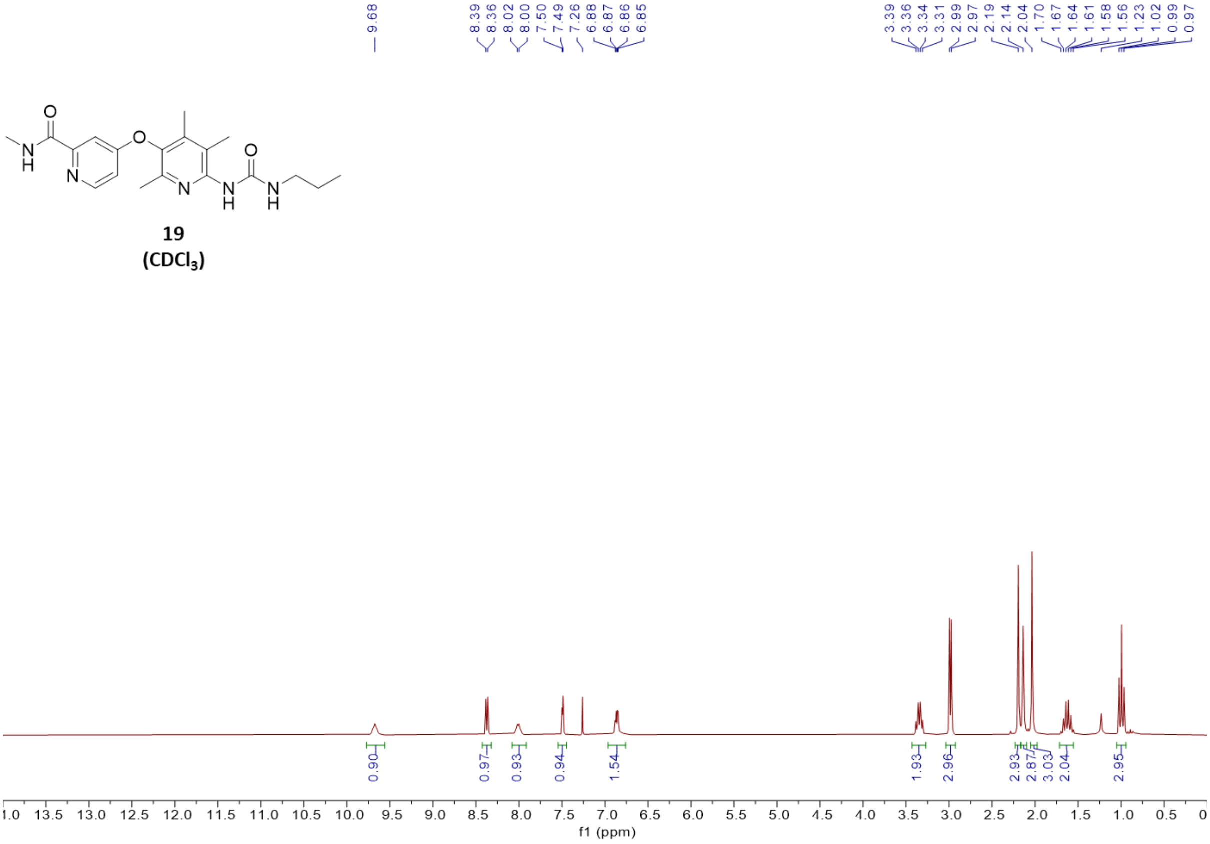

19
($CDCl_3$)

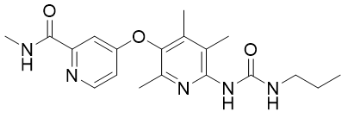

19
($CDCl_3$)

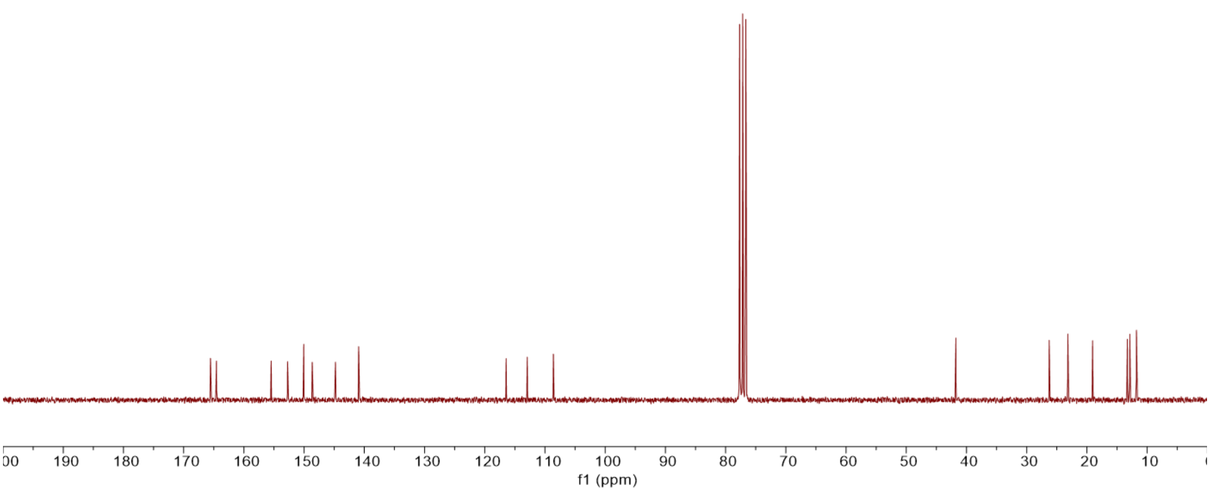

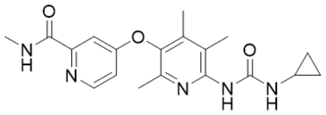

20
($CDCl_3$)

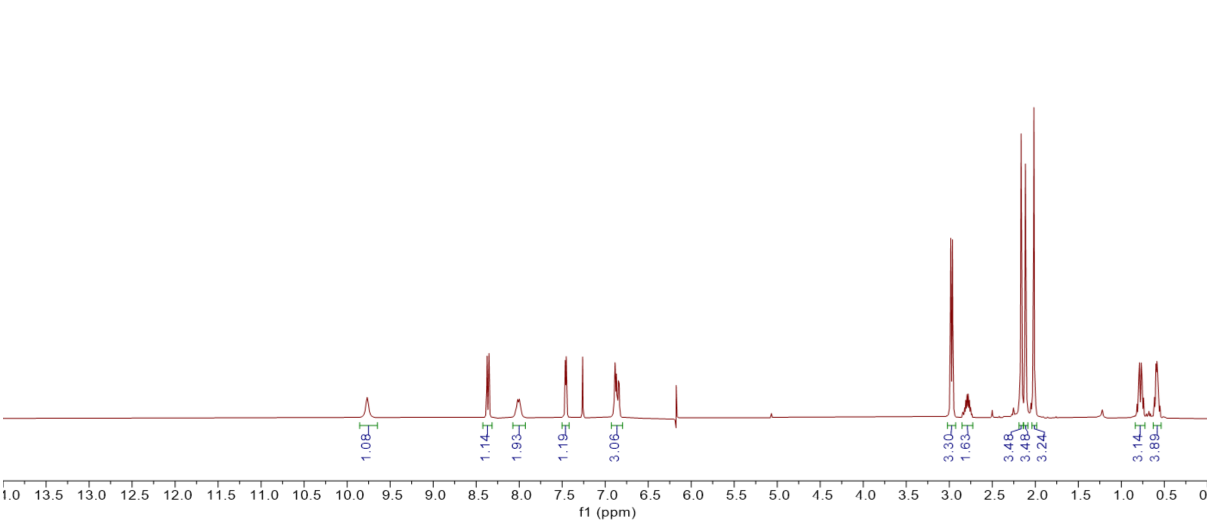

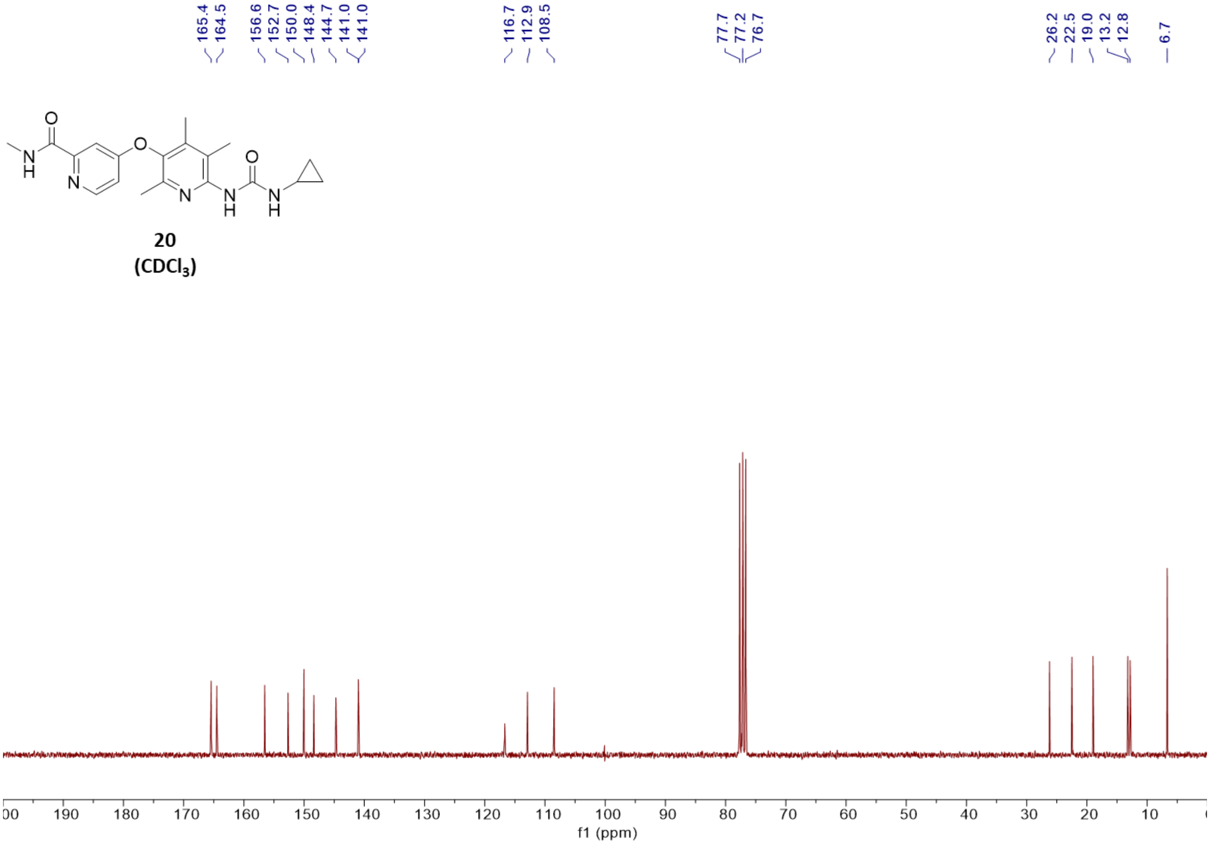

20
($CDCl_3$)

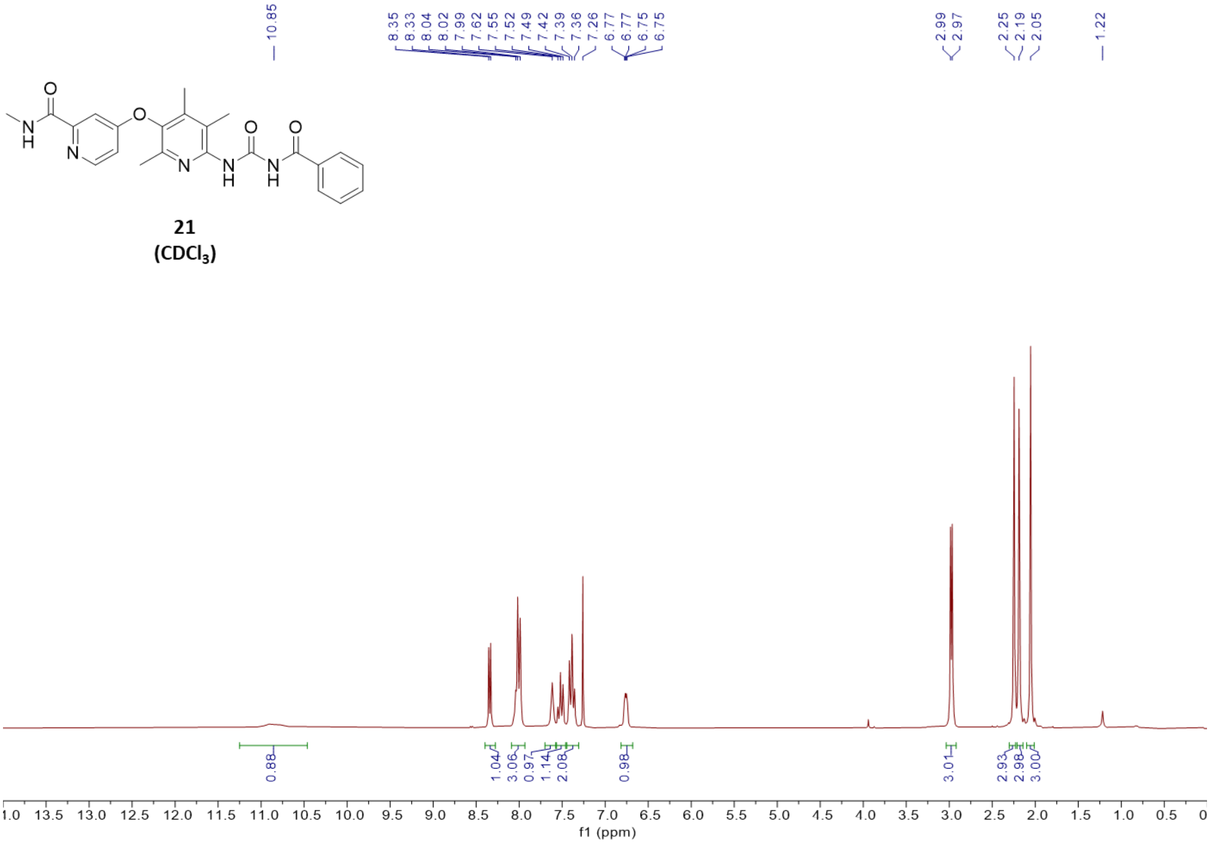

21
($CDCl_3$)

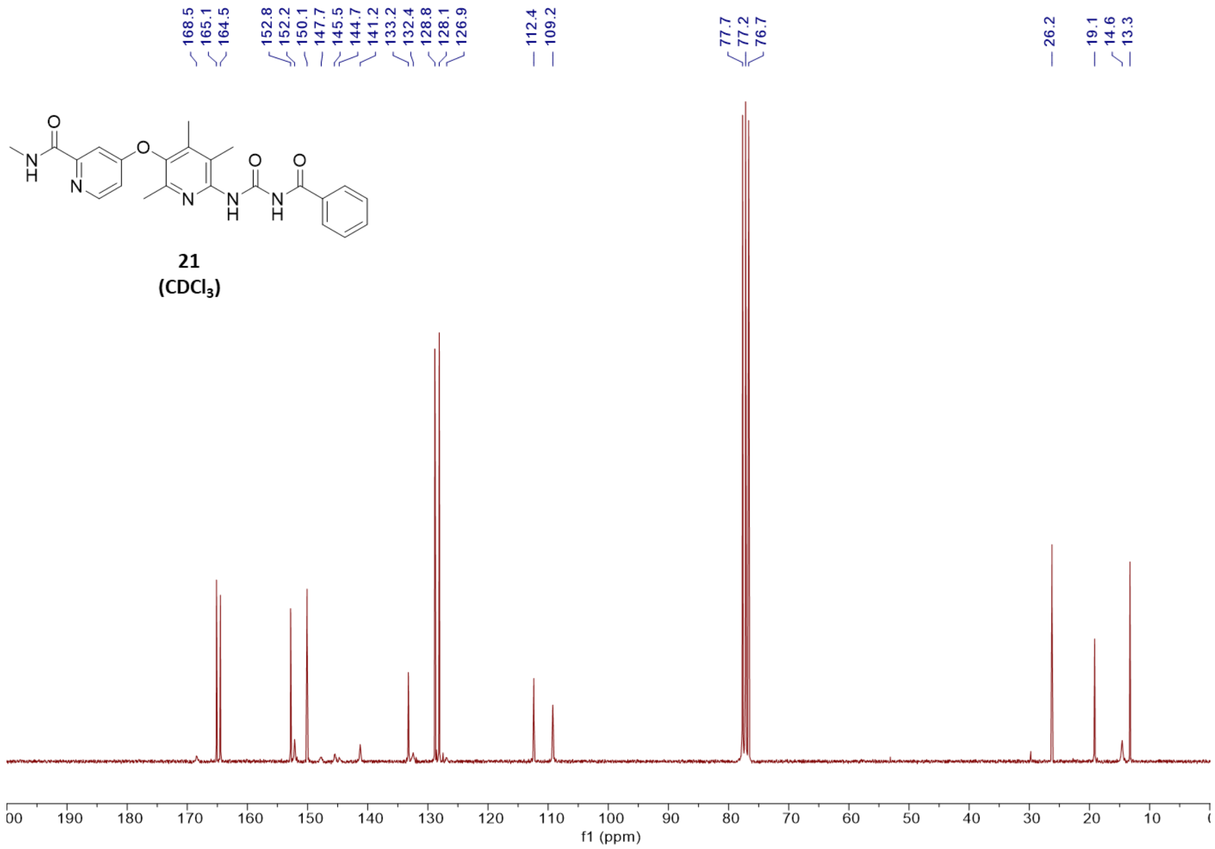

21
($CDCl_3$)

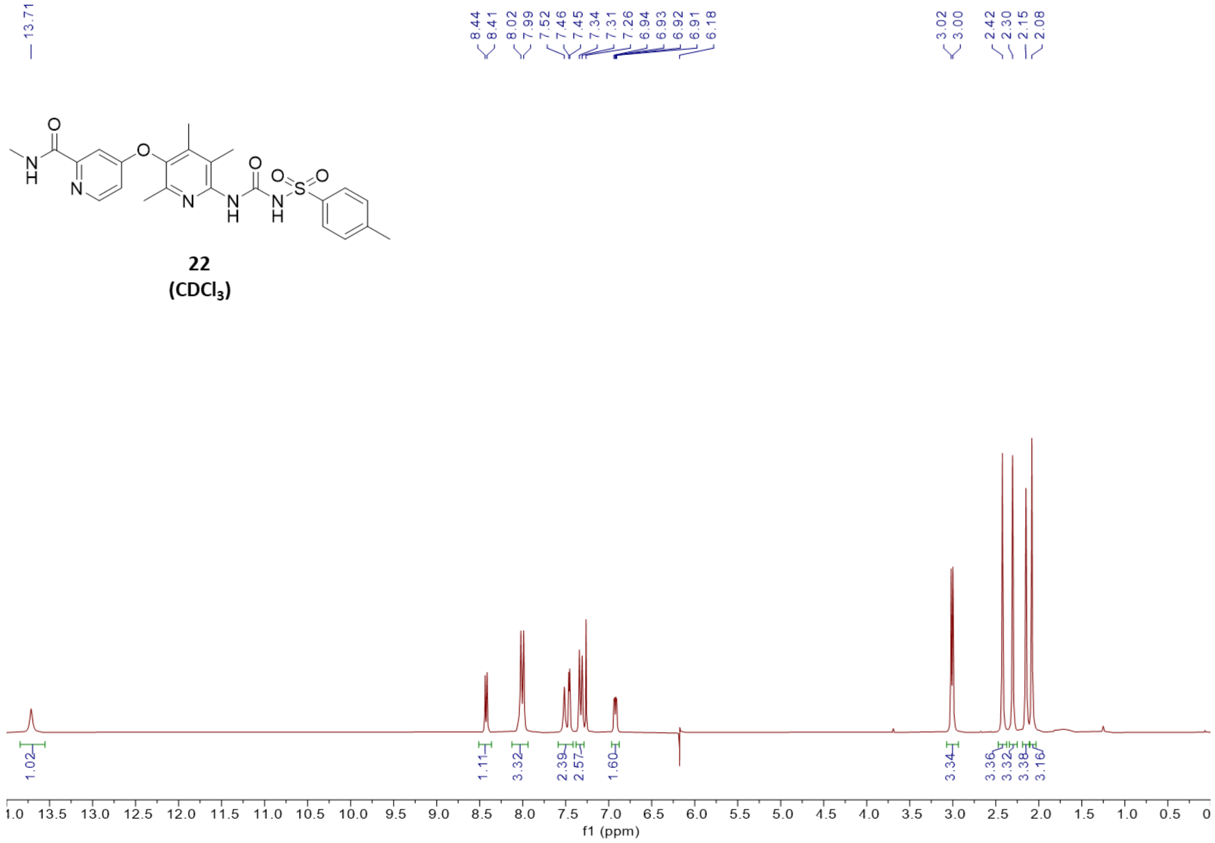

22
($CDCl_3$)

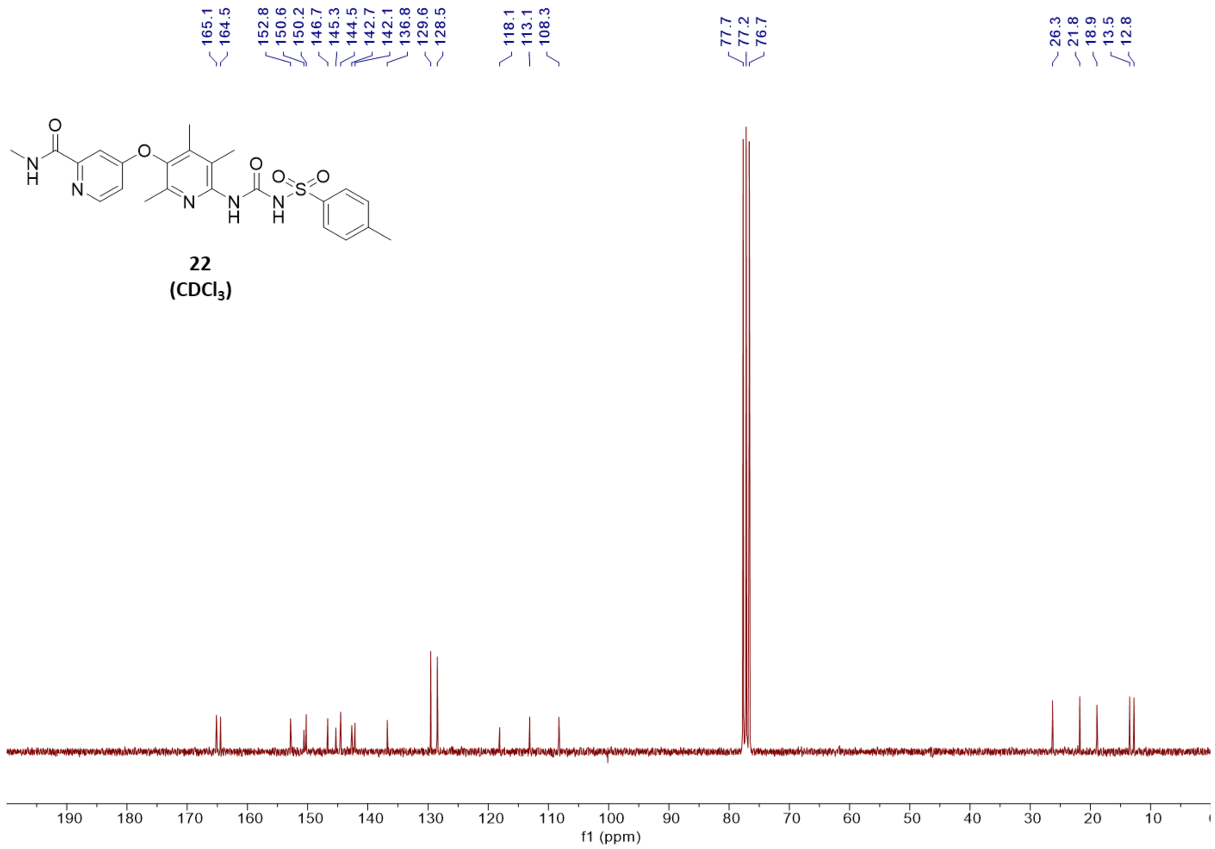

22
($CDCl_3$)

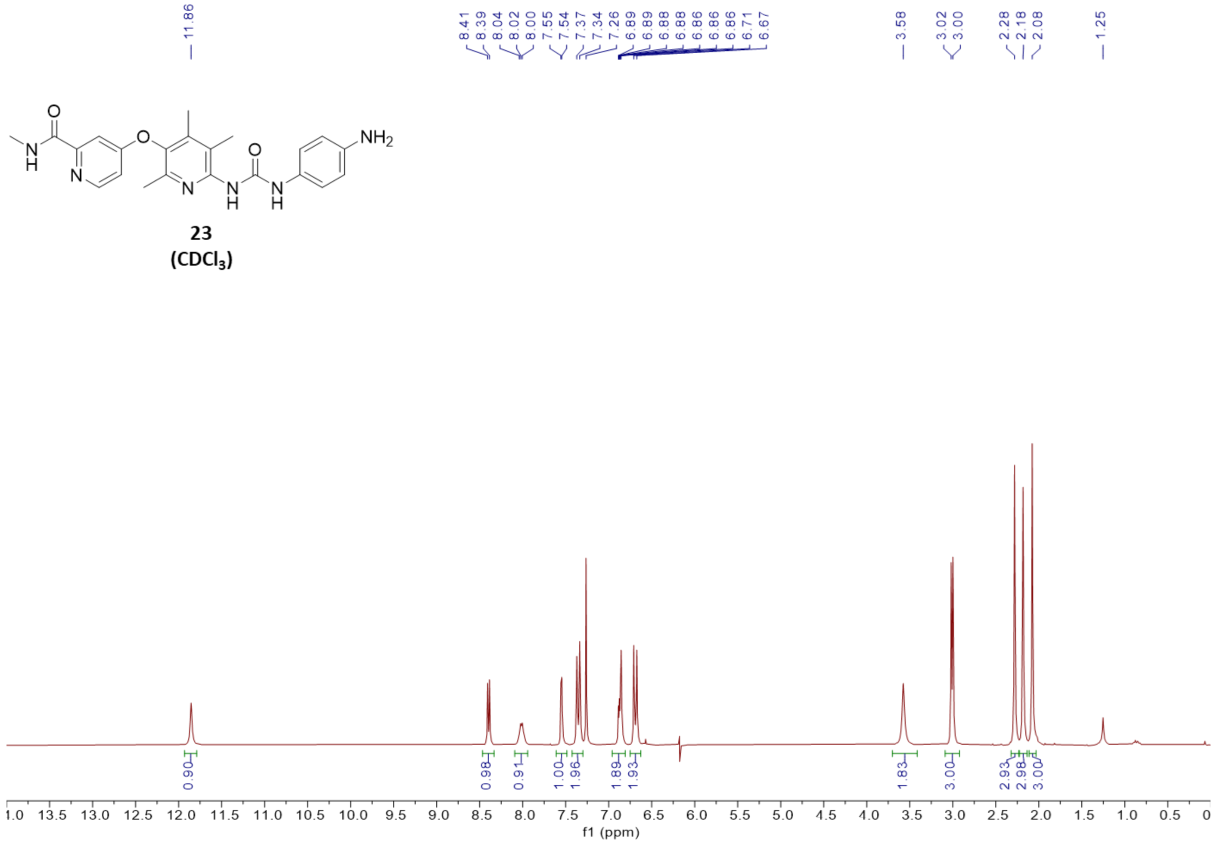

23
($CDCl_3$)

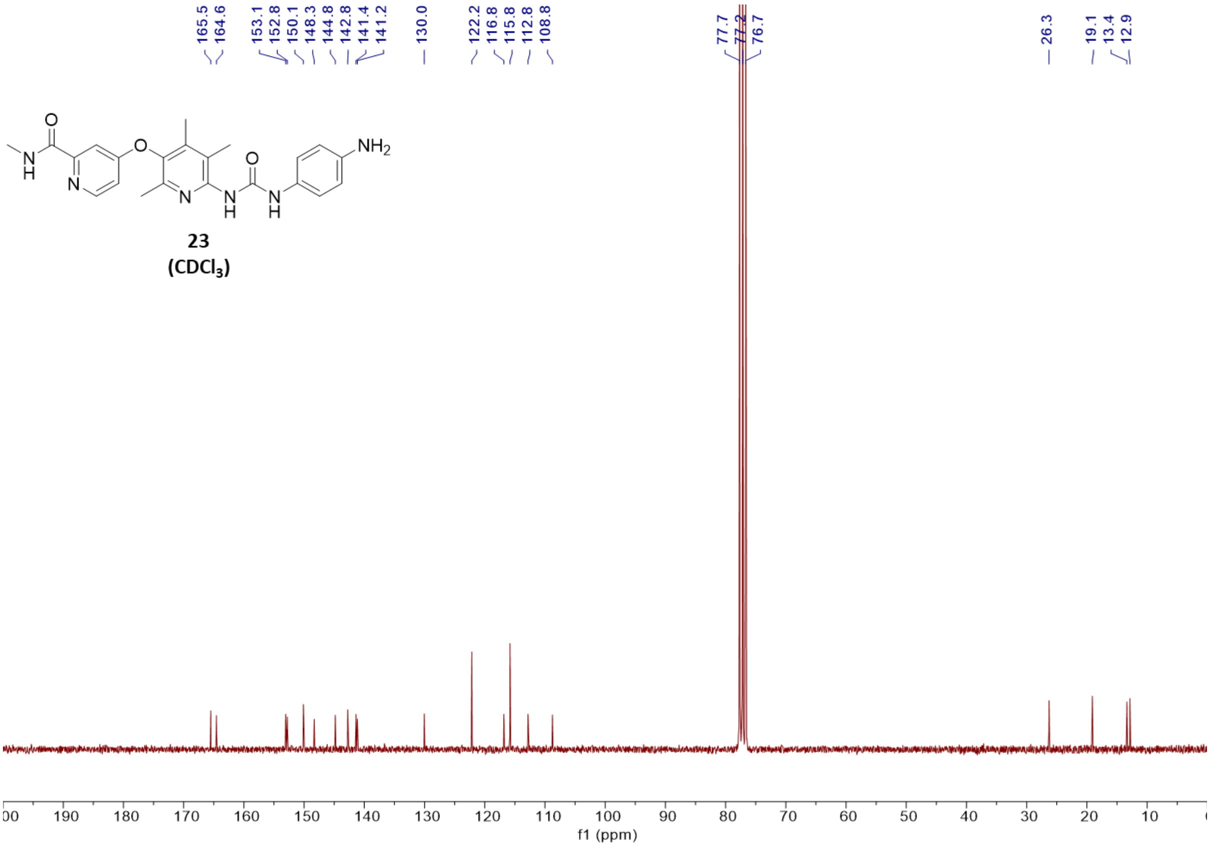
165.5
164.6
153.1
152.8
150.1
148.3
144.8
142.8
141.4
141.2
130.0
122.2
116.8
115.8
112.8
108.8
77.7
77.2
76.7
26.3
19.1
13.4
12.9
O
N
H
N
O
N
O
N
H
N
H
NH2
23
(CDCl3)
f1 (ppm)

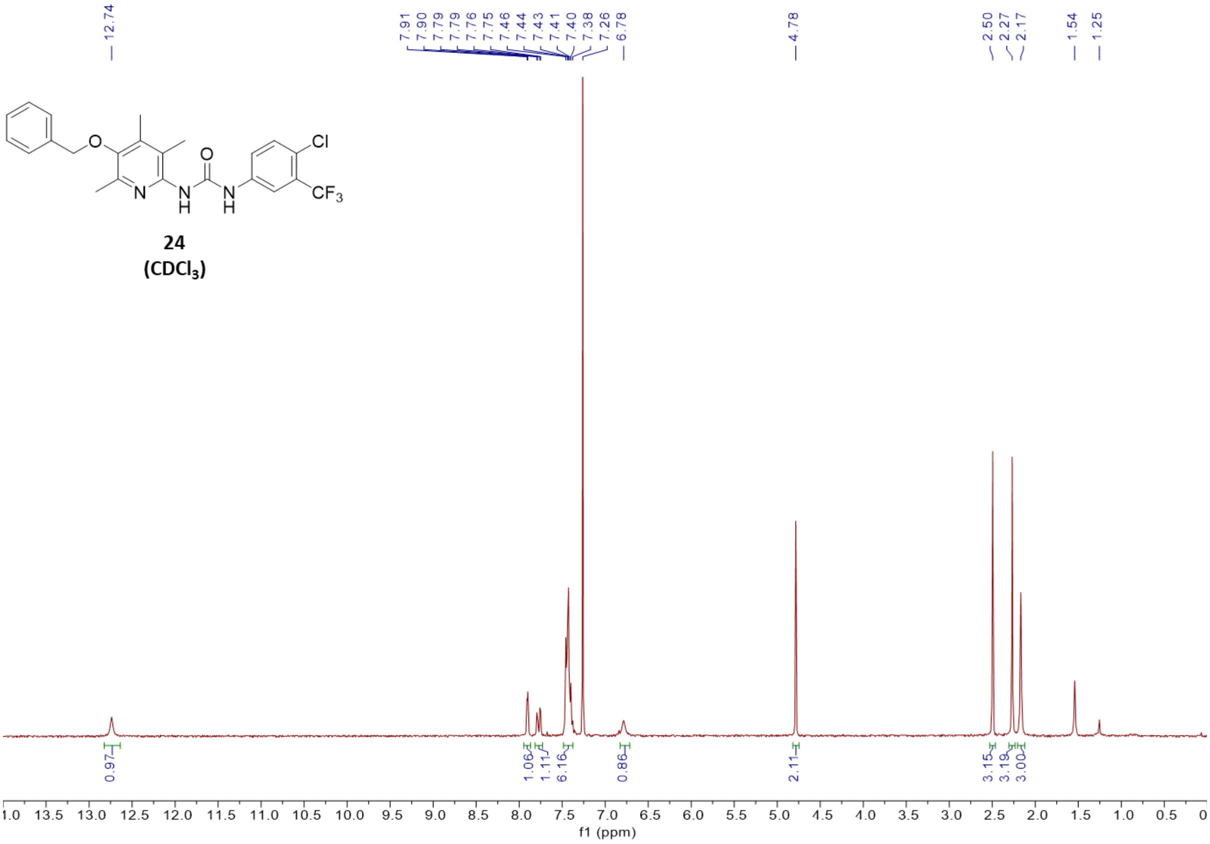
12.74
7.91
7.90
7.79
7.79
7.76
7.75
7.46
7.44
7.43
7.41
7.40
7.38
7.26
6.78
4.78
2.50
2.27
2.17
1.54
1.25
O
N
N
H
O
N
H
Cl
CF3
24
(CDCl3)
0.97
1.06
1.11
6.16
0.86
2.11
3.15
3.19
3.00
f1 (ppm)

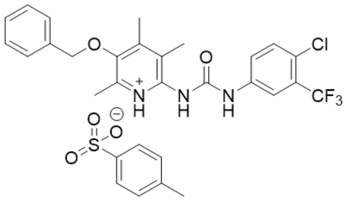

24-1
(CD_3OD)

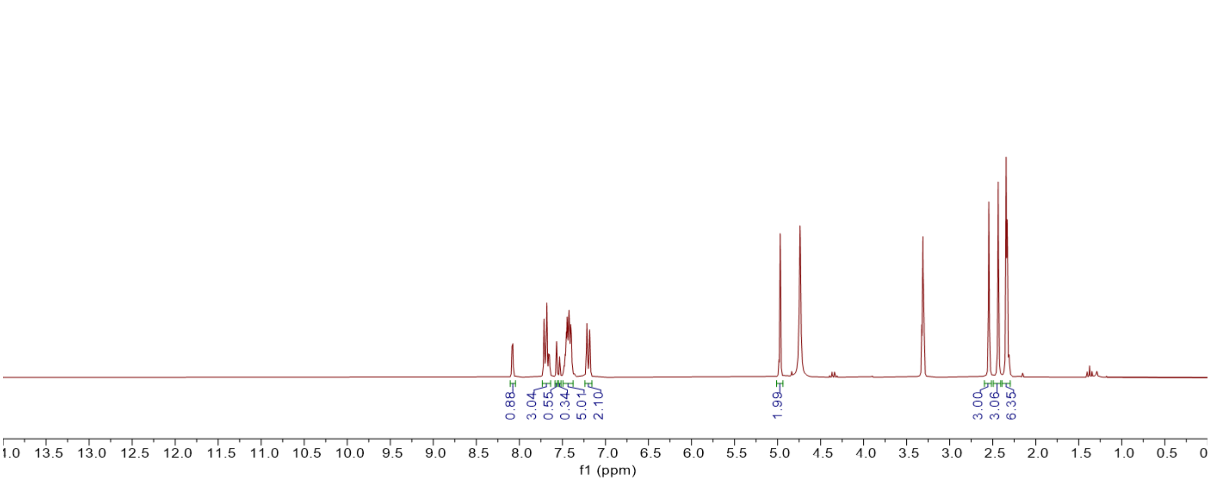

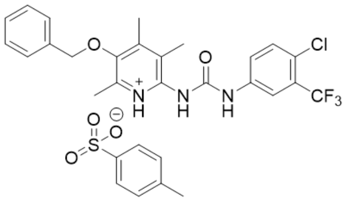

24-1
(CD_3OD)

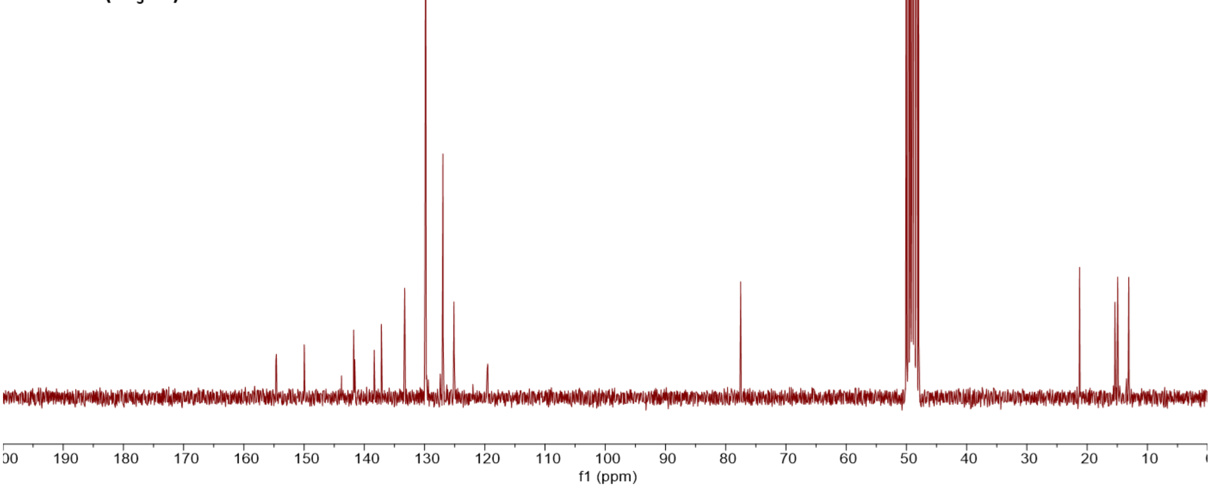

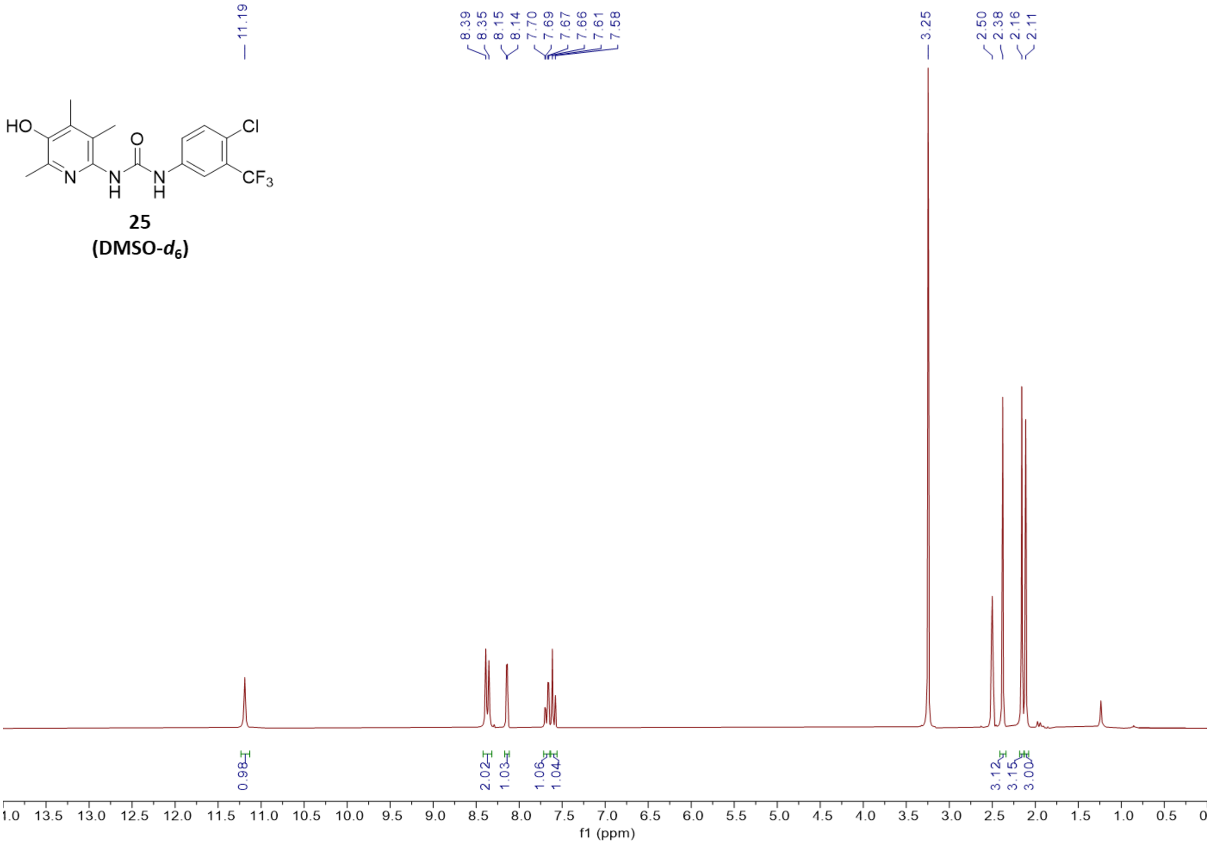
11.19
8.39
8.35
8.15
8.14
7.70
7.69
7.67
7.66
7.61
7.58
3.25
2.50
2.38
2.16
2.11
HO
N
N
H
O
N
H
Cl
CF_3
25
(DMSO-d_6)
0.98
2.02
1.03
1.06
1.04
3.12
3.15
3.00
f1 (ppm)

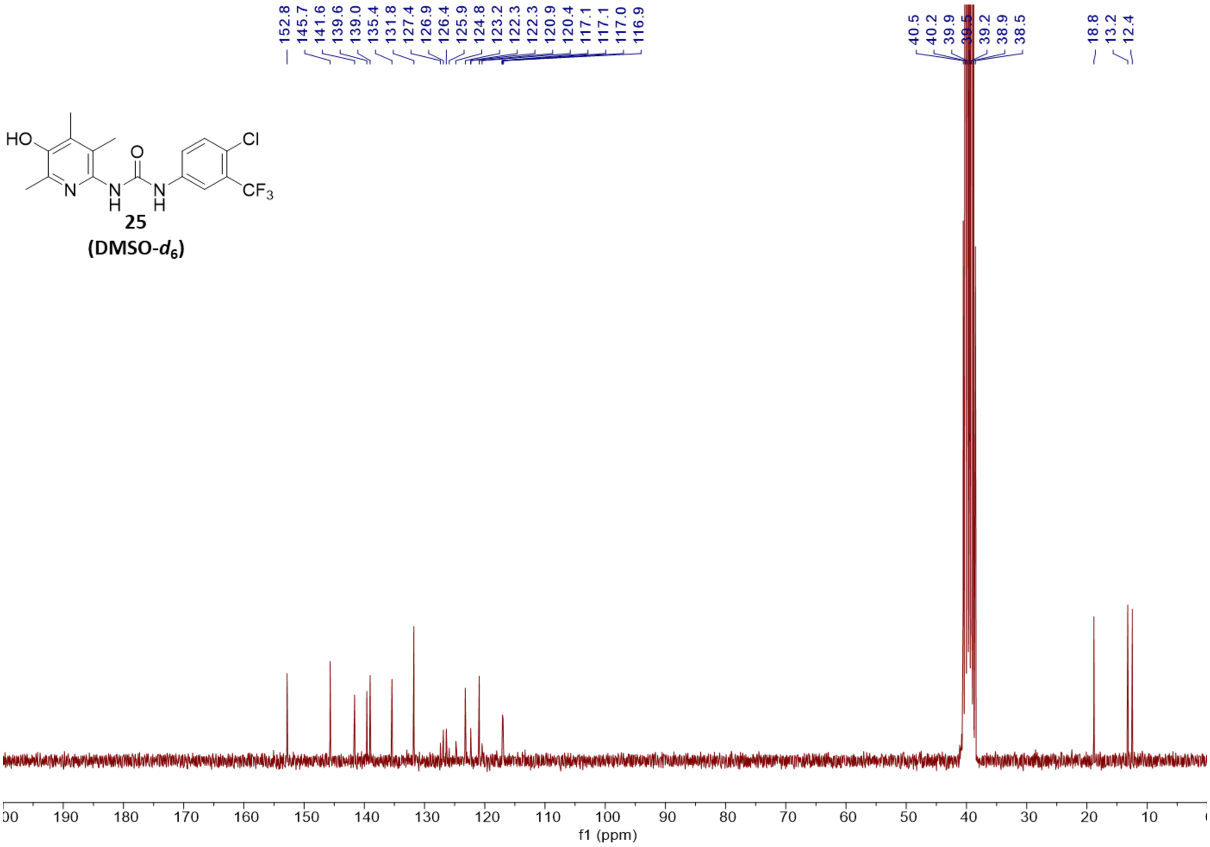
152.8
145.7
141.6
139.6
139.0
135.4
131.8
127.4
126.9
126.4
125.9
124.8
123.2
122.3
122.3
120.9
120.4
117.1
117.1
117.0
116.9
40.5
40.2
39.9
39.5
39.2
38.9
38.5
18.8
13.2
12.4
HO
N
N
H
O
N
H
Cl
CF_3
25
(DMSO-d_6)
f1 (ppm)

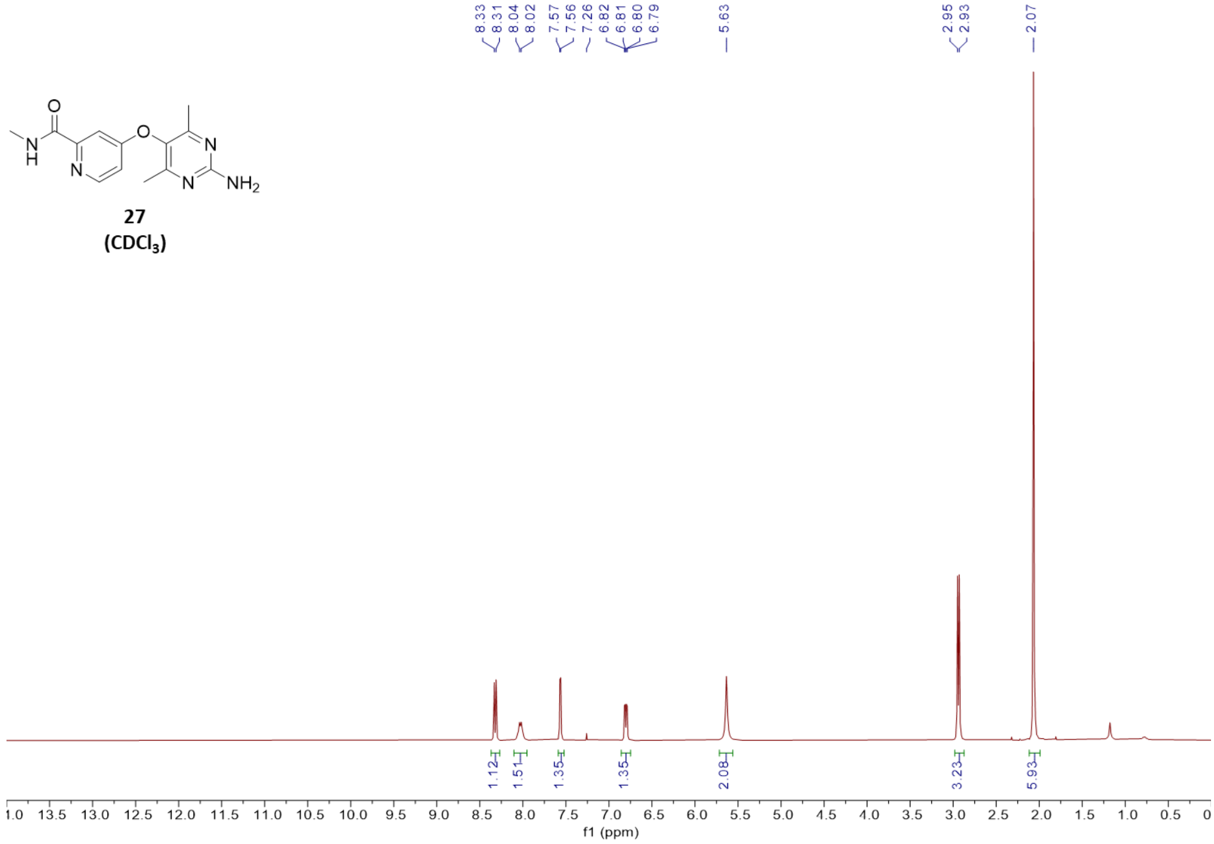

27
($CDCl_3$)

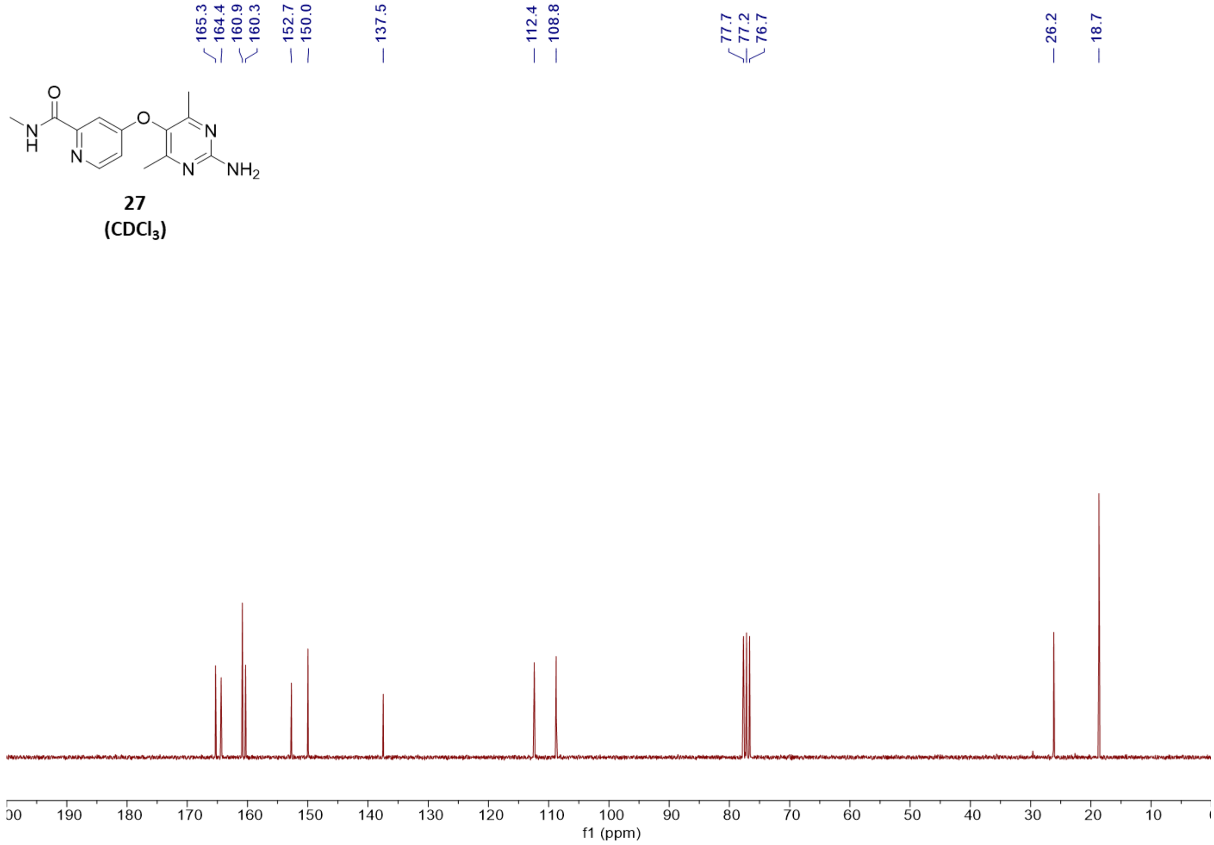

27
($CDCl_3$)

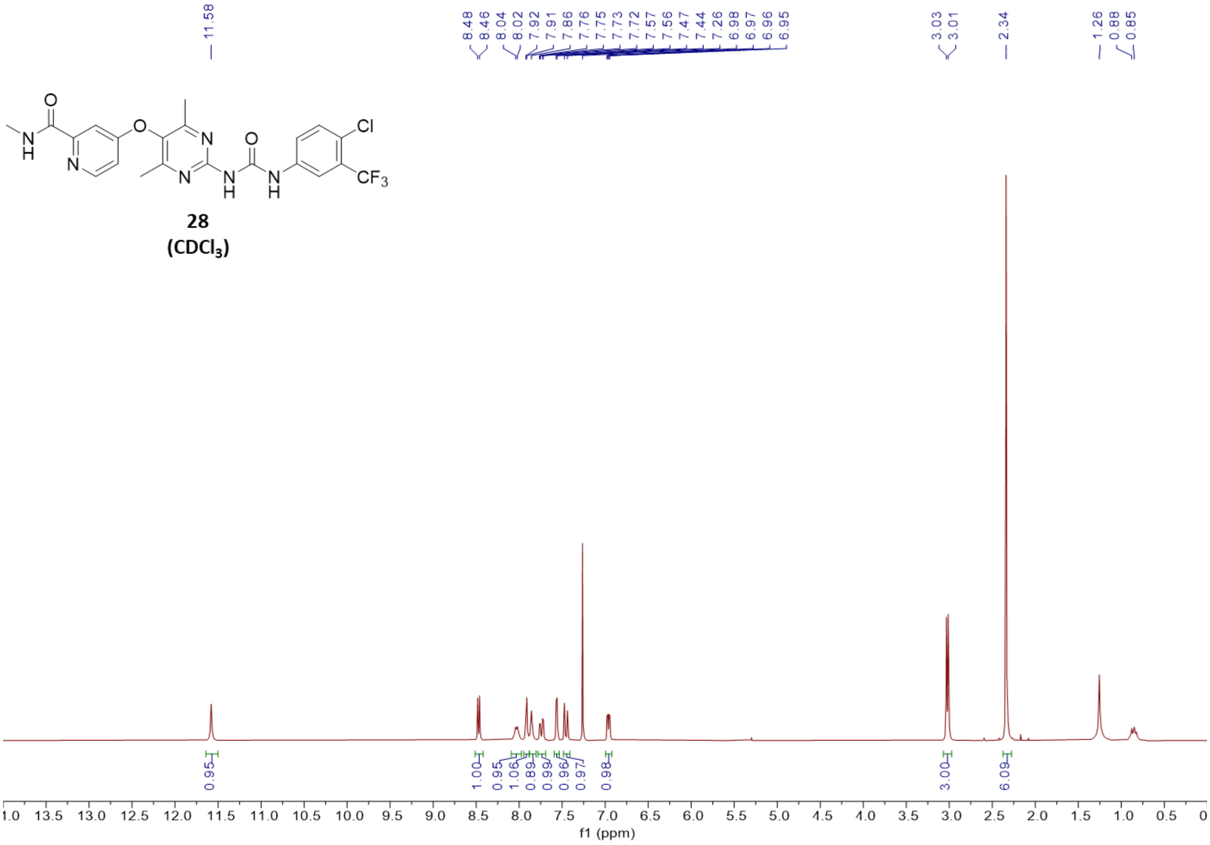

28
(CDCl$_3$)

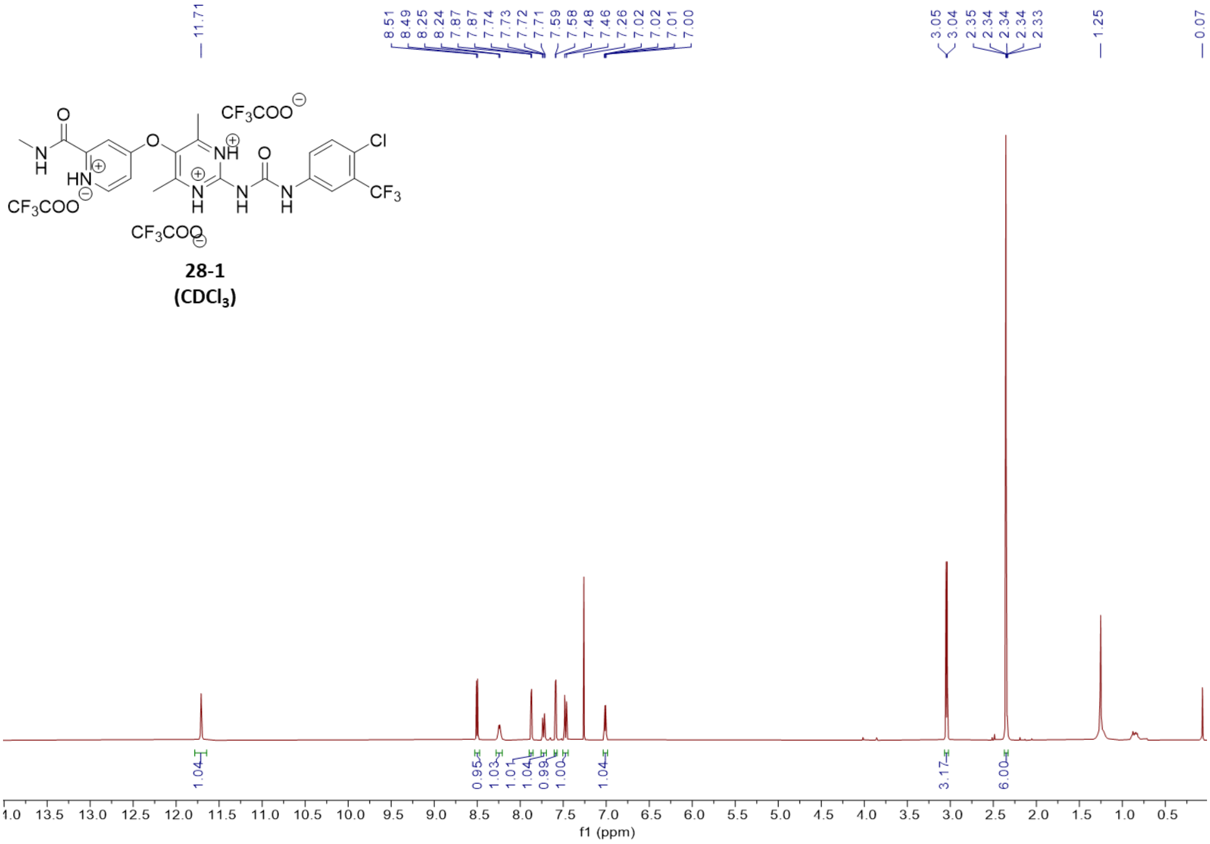

28-1
(CDCl$_3$)

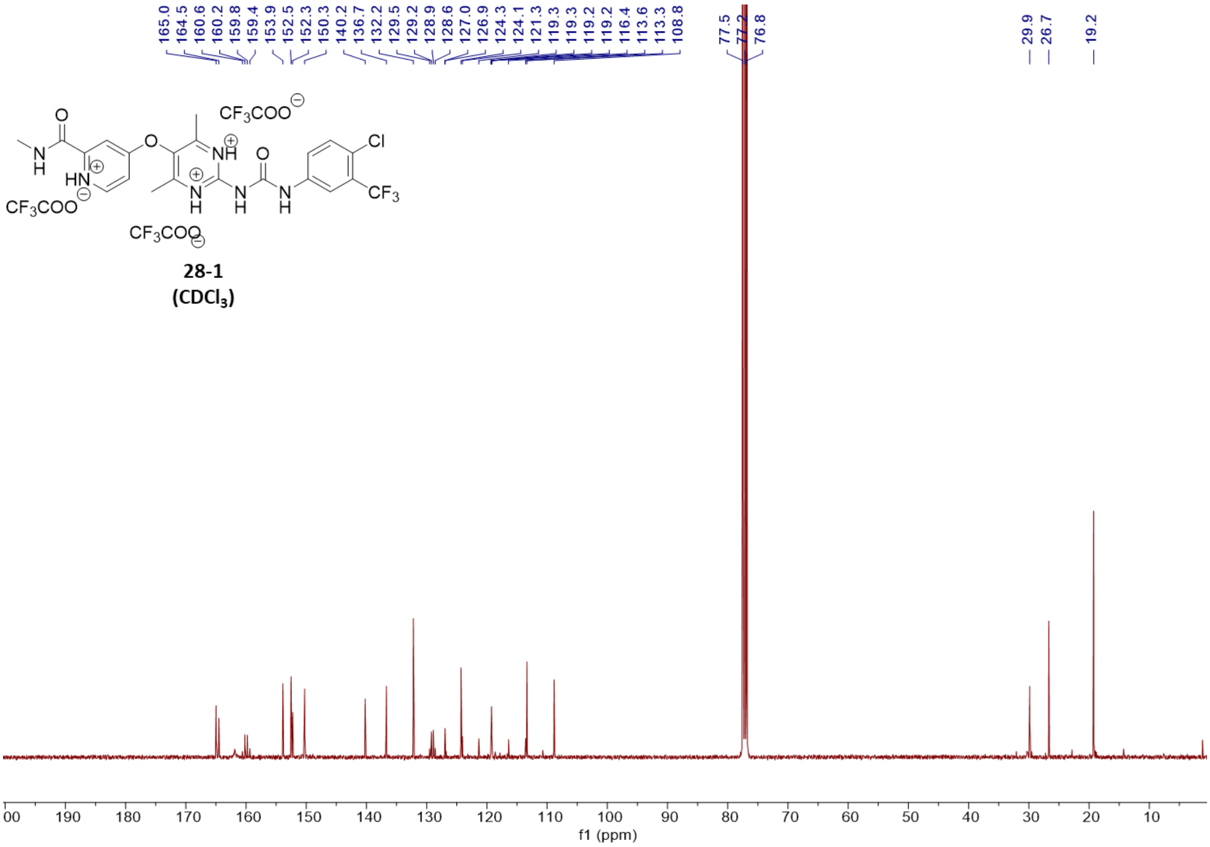

165.0
164.5
160.6
160.2
159.8
159.4
153.9
152.5
152.3
150.3
140.2
136.7
132.2
129.5
129.2
128.9
128.6
127.0
126.9
124.3
124.1
121.3
119.3
119.3
119.2
119.2
116.4
113.6
113.3
108.8
77.5
77.2
76.8
29.9
26.7
19.2
f1 (ppm)

28-1
($CDCl_3$)

<Chromatogram>

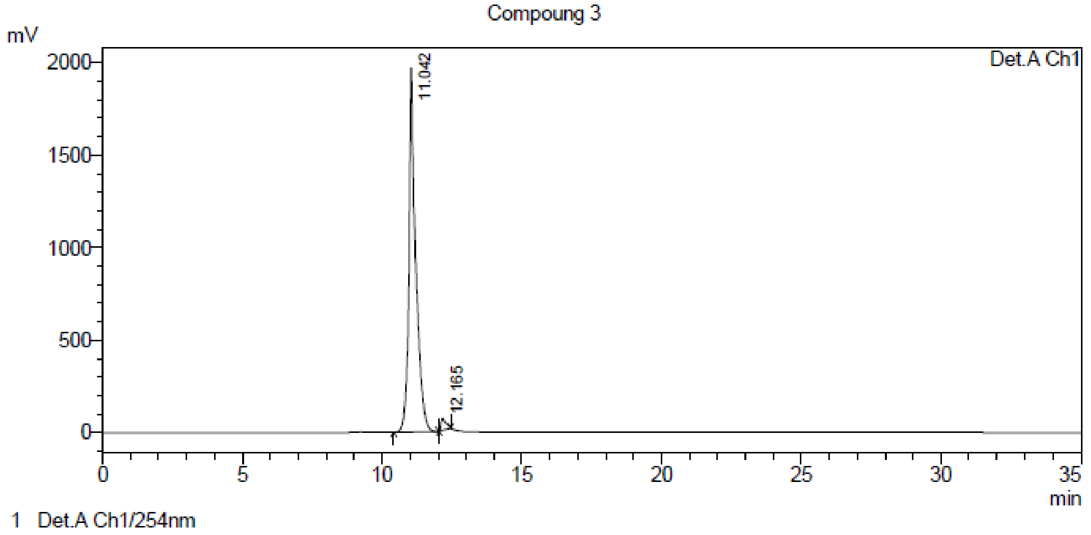

PeakTable

Detector A Ch1 254nm

Peak#	Ret. Time	Area	Height	Area %	Height %
1	11.042	33596459	1965100	97.806	96.955
2	12.165	753654	61720	2.194	3.045
Total		34350113	2026820	100.000	100.000

<Chromatogram>

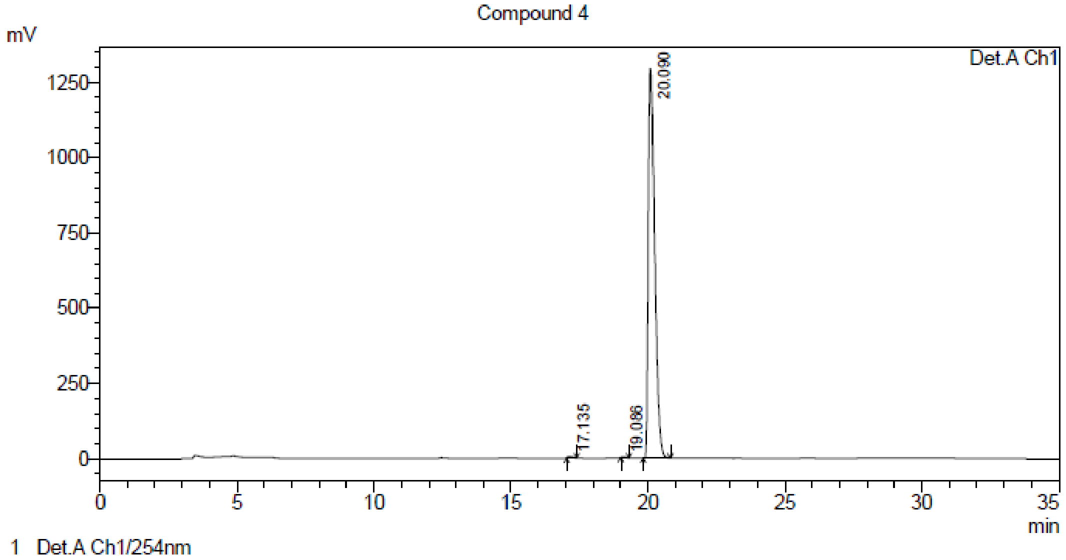

PeakTable

Detector A Ch1 254nm

Peak#	Ret. Time	Area	Height	Area %	Height %
1	17.135	60078	4885	0.290	0.376
2	19.086	27884	2495	0.134	0.192
3	20.090	20660354	1292729	99.576	99.432
Total		20748316	1300108	100.000	100.000

<Chromatogram>

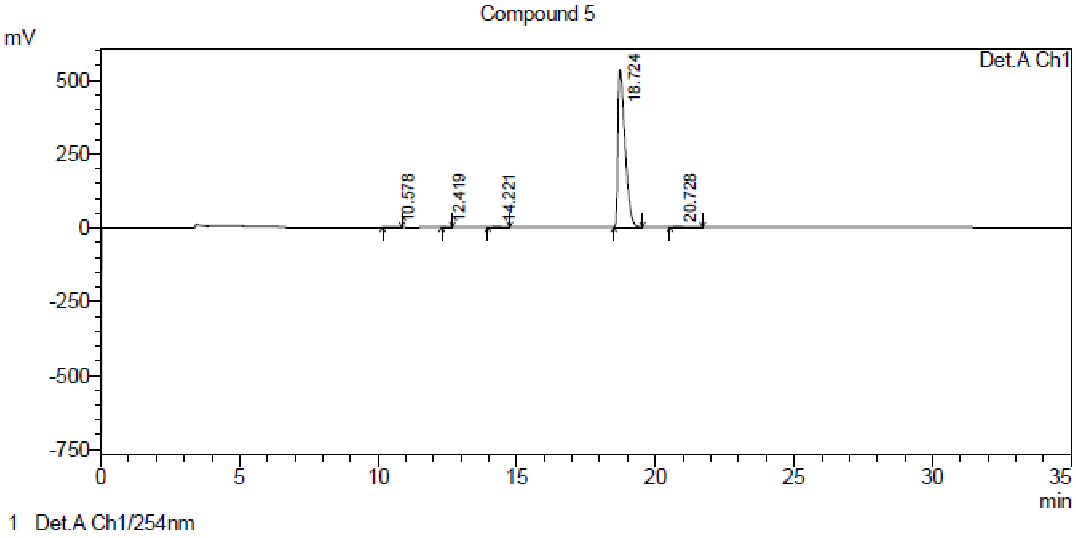

PeakTable

Detector A Ch1 254nm

Peak#	Ret. Time	Area	Height	Area %	Height %
1	10.578	15494	1014	0.162	0.188
2	12.419	9582	875	0.100	0.162
3	14.221	16288	650	0.170	0.121
4	18.724	9466298	533972	98.968	99.058
5	20.728	57337	2540	0.599	0.471
Total		9564998	539050	100.000	100.000

<Chromatogram>

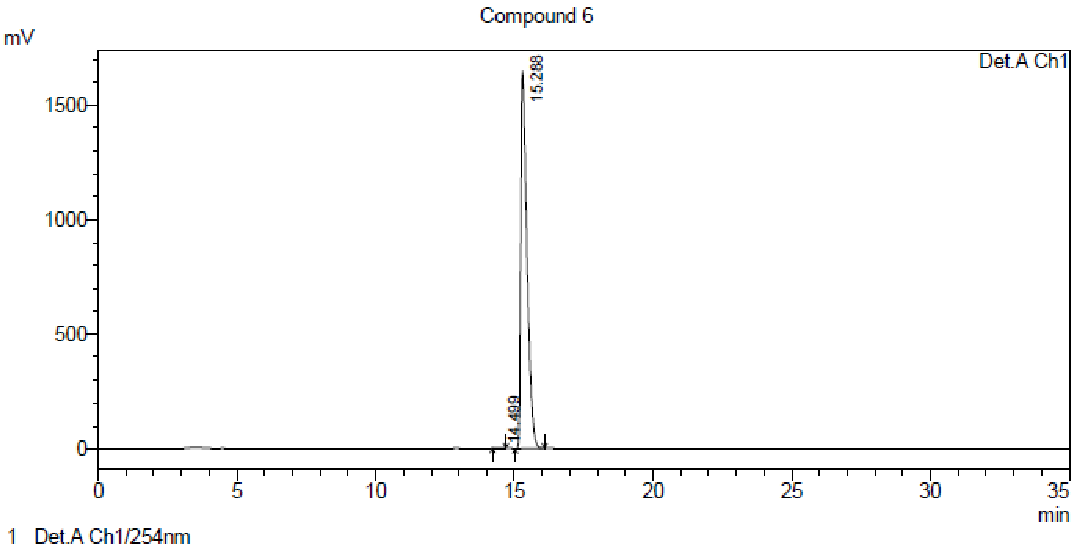

PeakTable

Detector A Ch1 254nm

Peak#	Ret. Time	Area	Height	Area %	Height %
1	14.499	27264	1494	0.105	0.091
2	15.288	25871604	1645308	99.895	99.909
Total		25898868	1646803	100.000	100.000

<Chromatogram>

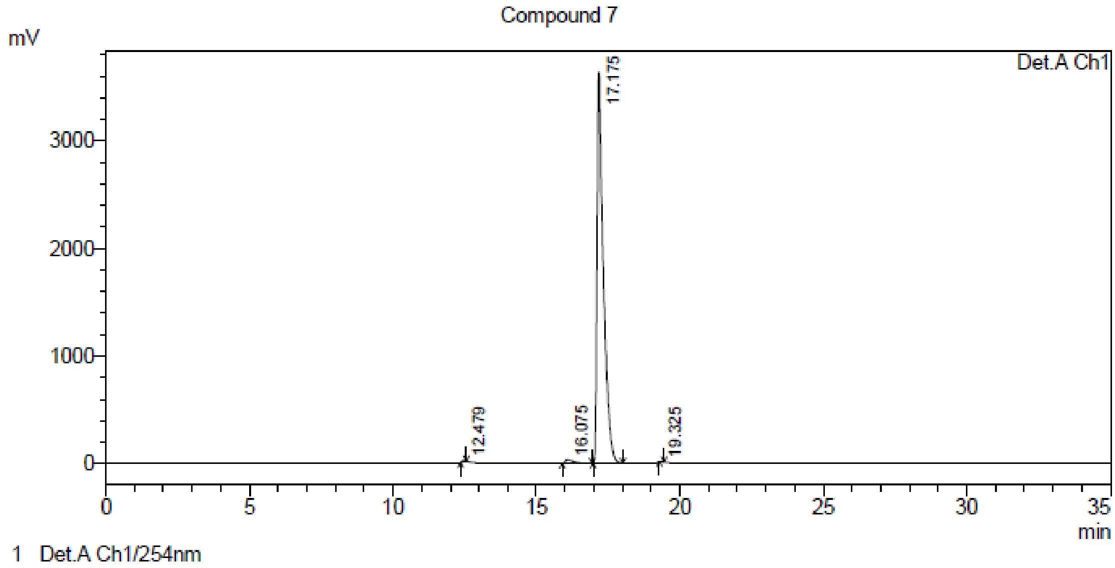

PeakTable

Detector A Ch1 254nm

Peak#	Ret. Time	Area	Height	Area %	Height %
1	12.479	116254	14753	0.208	0.400
2	16.075	618216	33729	1.106	0.916
3	17.175	55115934	3631835	98.644	98.578
4	19.325	23220	3894	0.042	0.106
Total		55873624	3684212	100.000	100.000

<Chromatogram>

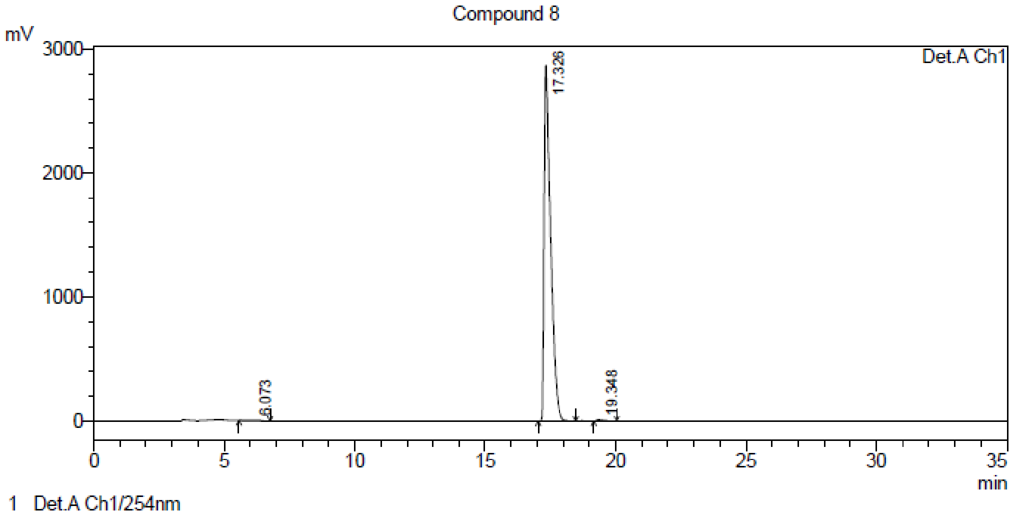

PeakTable

Detector A Ch1 254nm

Peak#	Ret. Time	Area	Height	Area %	Height %
1	6.073	134380	3766	0.272	0.131
2	17.326	49065317	2862625	99.386	99.516
3	19.348	168566	10153	0.341	0.353
Total		49368263	2876543	100.000	100.000

<Chromatogram>

Compound 9

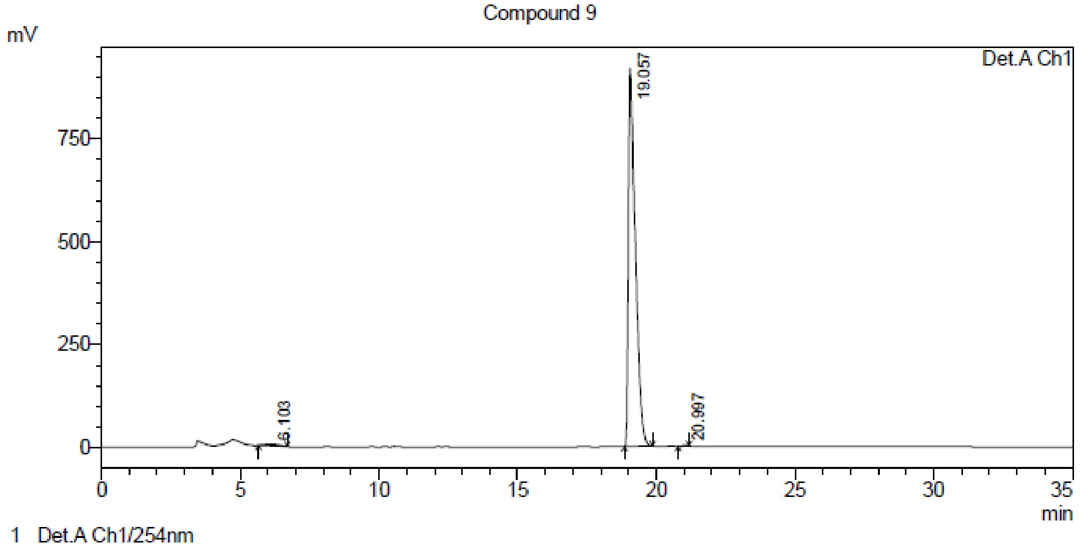

1 Det.A Ch1/254nm

PeakTable

Detector A Ch1 254nm

Peak#	Ret. Time	Area	Height	Area %	Height %
1	6.103	151885	4263	0.942	0.461
2	19.057	15947781	919150	98.925	99.320
3	20.997	21358	2027	0.132	0.219
Total		16121024	925440	100.000	100.000

<Chromatogram>

Compound 10

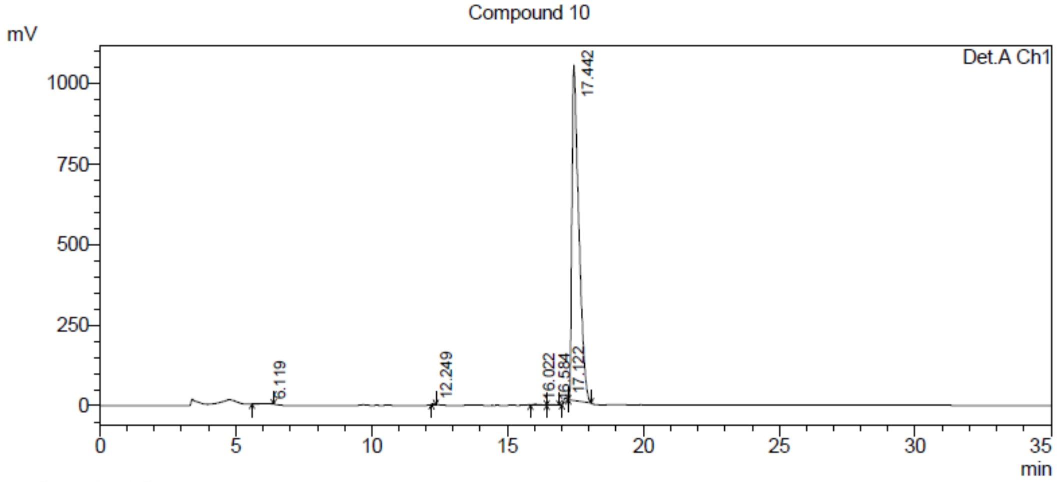

1 Det.A Ch1/254nm

PeakTable

Detector A Ch1 254nm

Peak#	Ret. Time	Area	Height	Area %	Height %
1	6.119	56710	2203	0.306	0.207
2	12.249	10211	2167	0.055	0.204
3	16.022	64720	3388	0.349	0.319
4	16.584	28975	1848	0.156	0.174
5	17.122	106257	12928	0.573	1.217
6	17.442	18277442	1039572	98.561	97.878
Total		18544315	1062105	100.000	100.000

<Chromatogram>

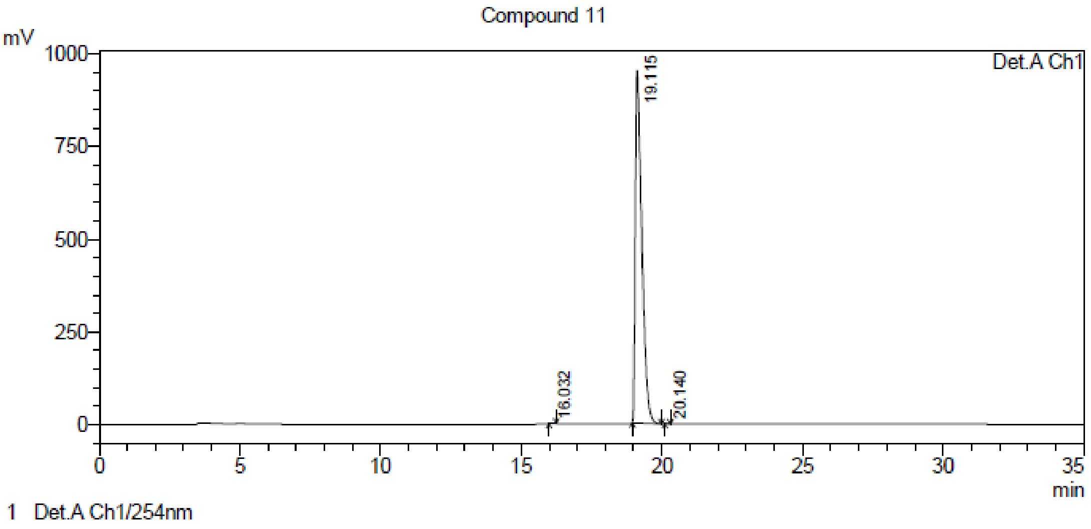

PeakTable

Detector A Ch1 254nm

Peak#	Ret. Time	Area	Height	Area %	Height %
1	16.032	4234	410	0.027	0.043
2	19.115	15665398	951651	99.965	99.940
3	20.140	1186	163	0.008	0.017
Total		15670817	952224	100.000	100.000

<Chromatogram>

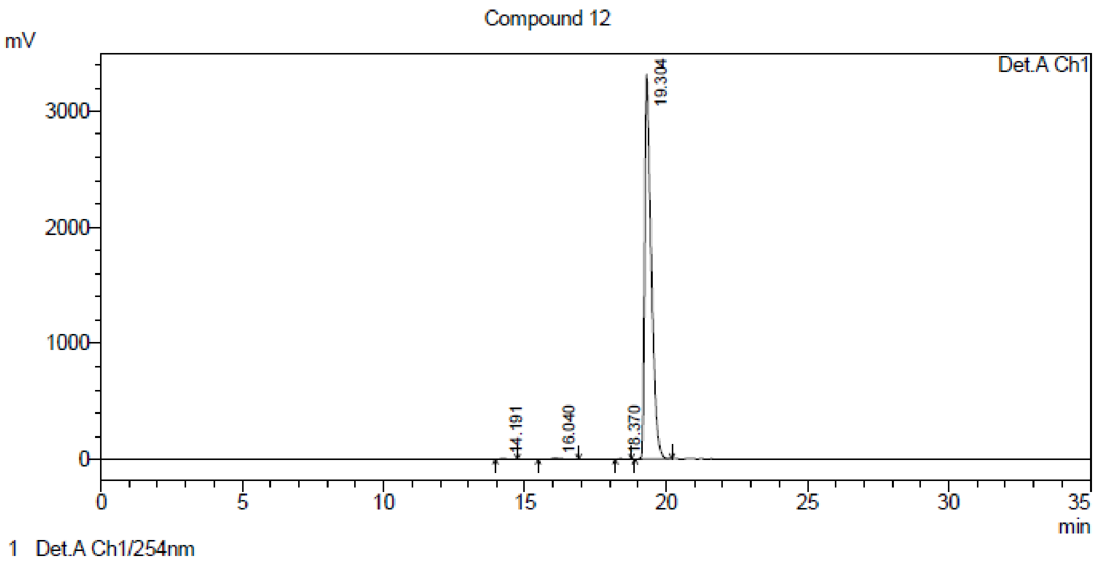

PeakTable

Detector A Ch1 254nm

Peak#	Ret. Time	Area	Height	Area %	Height %
1	14.191	101857	4465	0.189	0.134
2	16.040	216247	10339	0.402	0.311
3	18.370	45291	2808	0.084	0.084
4	19.304	53445193	3307876	99.325	99.470
Total		53808588	3325487	100.000	100.000

<Chromatogram>

Compound 13

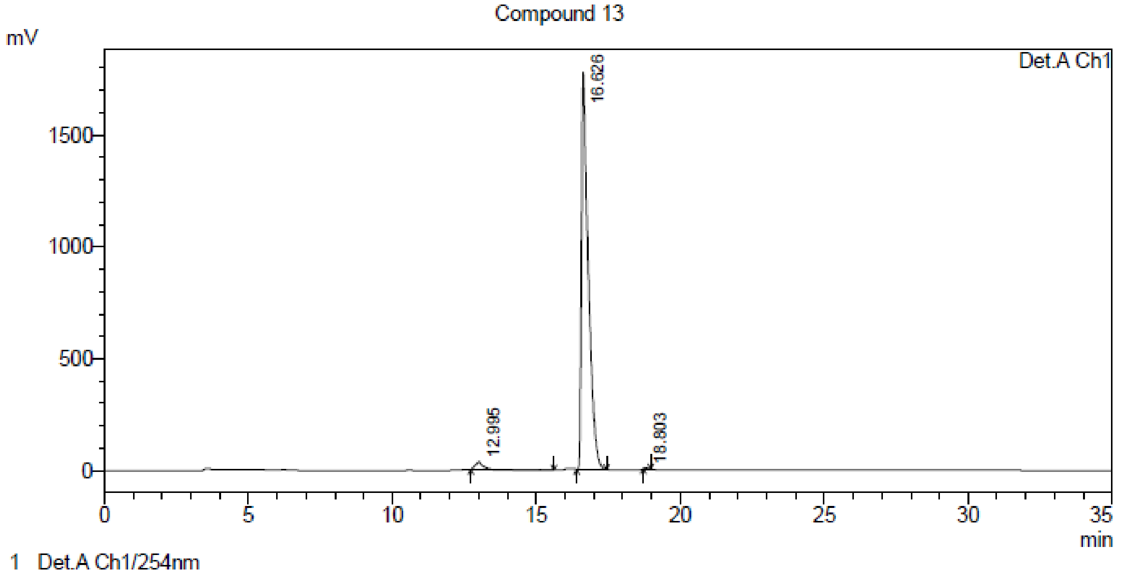

1 Det.A Ch1/254nm

PeakTable

Detector A Ch1 254nm

Peak#	Ret. Time	Area	Height	Area %	Height %
1	12.995	485816	34470	1.634	1.899
2	16.626	29197625	1776420	98.224	97.849
3	18.803	42001	4583	0.141	0.252
Total		29725442	1815472	100.000	100.000

<Chromatogram>

Compound 14

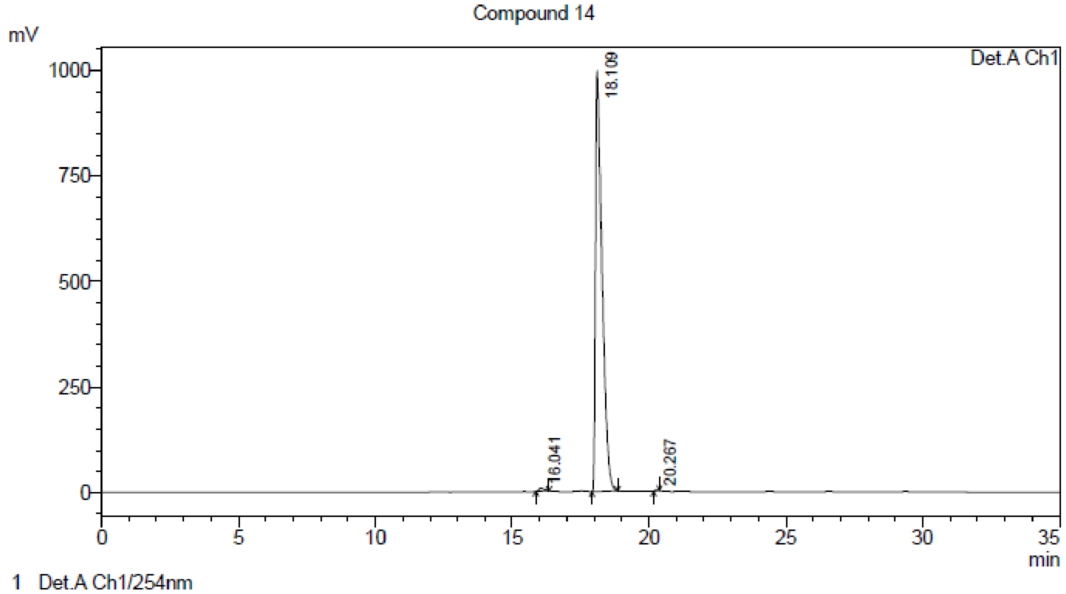

1 Det.A Ch1/254nm

PeakTable

Detector A Ch1 254nm

Peak#	Ret. Time	Area	Height	Area %	Height %
1	16.041	105150	7821	0.635	0.777
2	18.109	16449050	997192	99.276	99.039
3	20.267	14767	1852	0.089	0.184
Total		16568967	1006865	100.000	100.000

<Chromatogram>

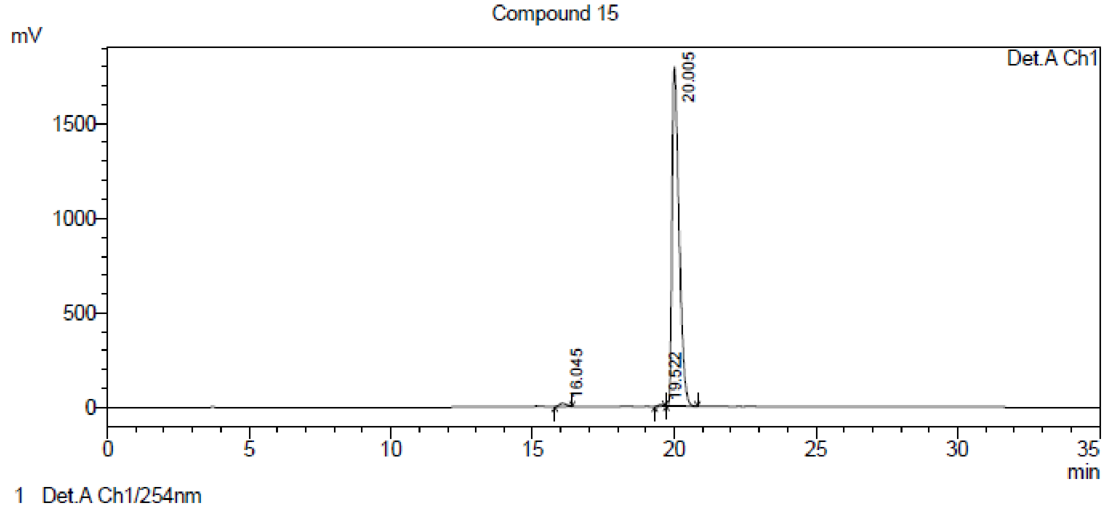

PeakTable

Detector A Ch1 254nm

Peak#	Ret. Time	Area	Height	Area %	Height %
1	16.045	303195	18619	0.966	1.024
2	19.522	87870	7575	0.280	0.416
3	20.005	30981395	1792907	98.753	98.560
Total		31372459	1819101	100.000	100.000

<Chromatogram>

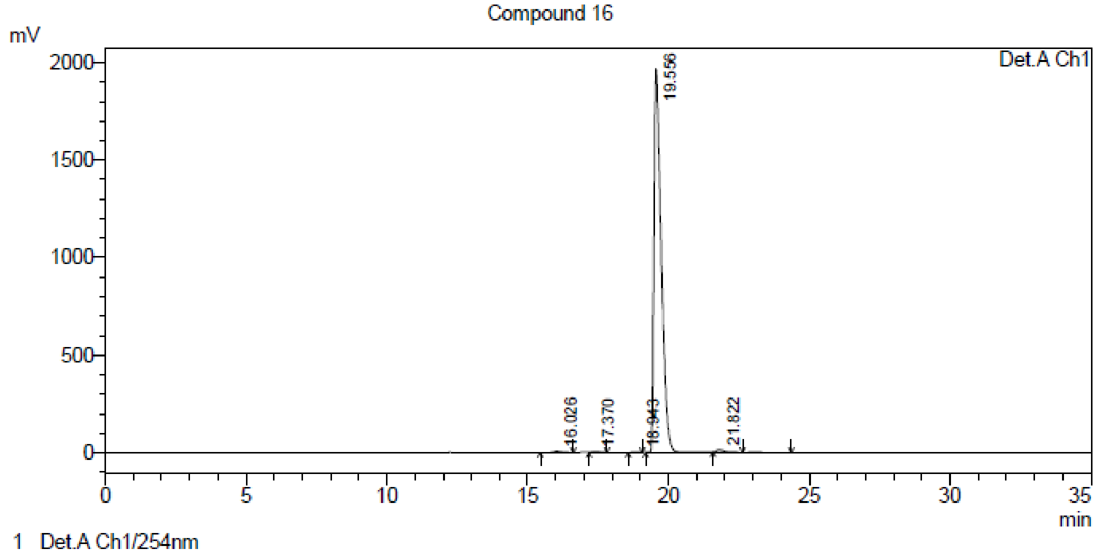

PeakTable

Detector A Ch1 254nm

Peak#	Ret. Time	Area	Height	Area %	Height %
1	16.026	201032	8119	0.551	0.407
2	17.370	136624	5995	0.374	0.301
3	18.943	65574	3224	0.180	0.162
4	19.556	35858447	1963586	98.264	98.548
5	21.822	230106	11601	0.631	0.582
Total		36491782	1992524	100.000	100.000

<Chromatogram>

Compound 17

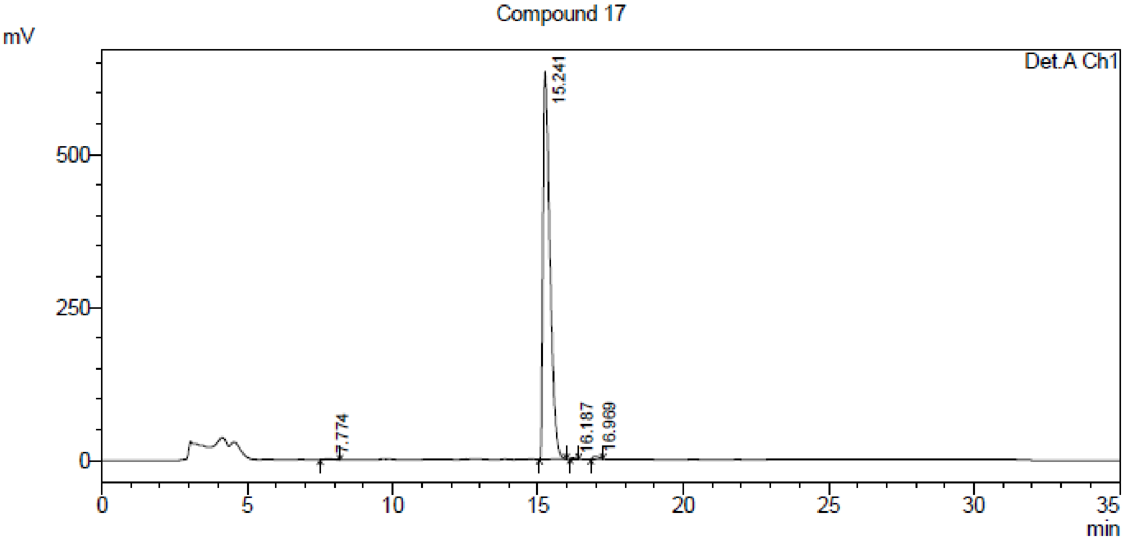

1 Det.A Ch1/254nm

PeakTable

Detector A Ch1 254nm

Peak#	Ret. Time	Area	Height	Area %	Height %
1	7.774	47055	2237	0.439	0.349
2	15.241	10586665	634034	98.866	98.793
3	16.187	13563	1207	0.127	0.188
4	16.969	60801	4304	0.568	0.671
Total		10708085	641782	100.000	100.000

<Chromatogram>

Compound 18

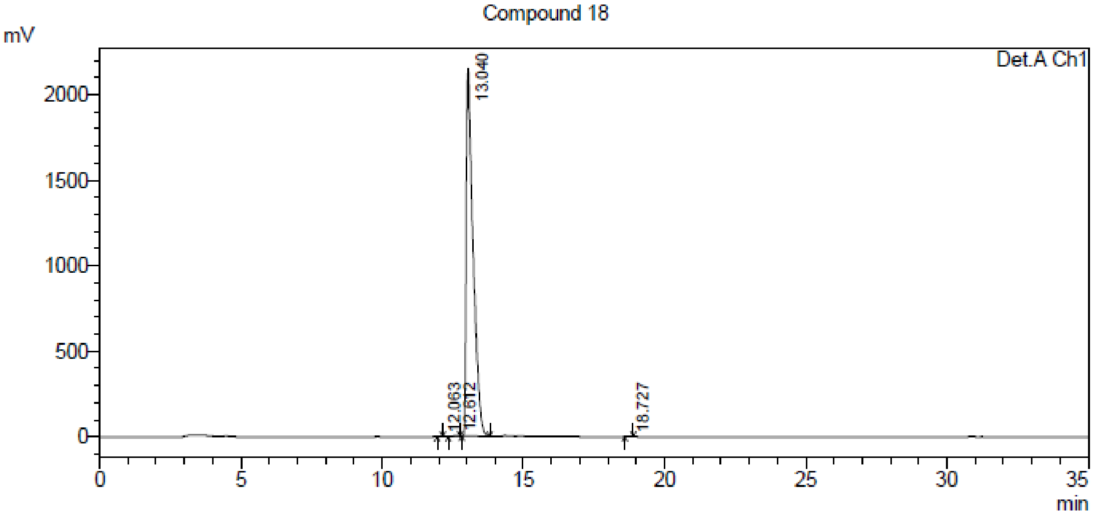

1 Det.A Ch1/254nm

PeakTable

Detector A Ch1 254nm

Peak#	Ret. Time	Area	Height	Area %	Height %
1	12.063	5992	934	0.017	0.043
2	12.612	35820	3229	0.101	0.150
3	13.040	35352242	2147173	99.824	99.720
4	18.727	20649	1866	0.058	0.087
Total		35414702	2153202	100.000	100.000

<Chromatogram>

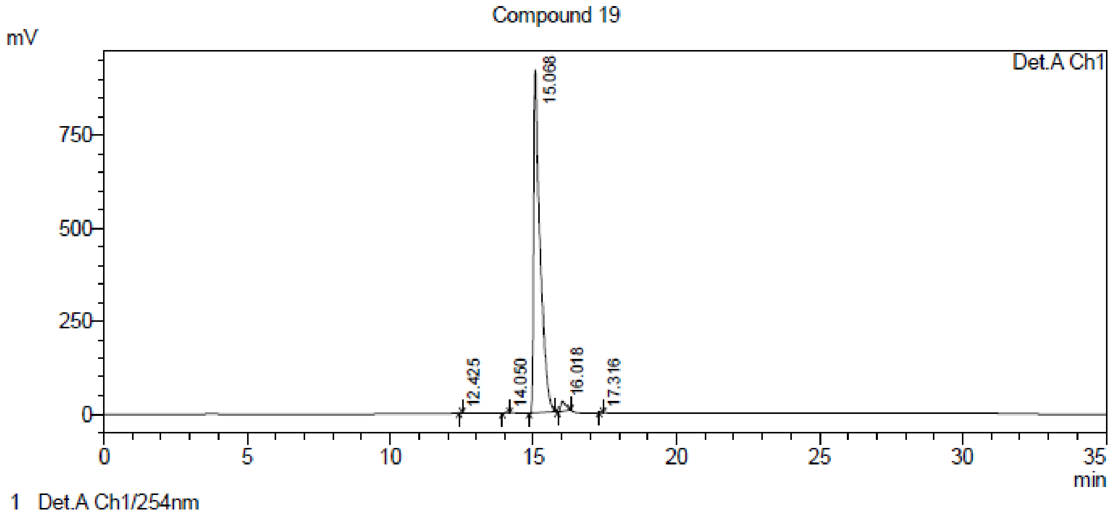

PeakTable

Detector A Ch1 254nm

Peak#	Ret. Time	Area	Height	Area %	Height %
1	12.425	1105	243	0.008	0.025
2	14.050	15441	2183	0.105	0.229
3	15.068	14340158	922686	97.591	96.918
4	16.018	335557	26593	2.284	2.793
5	17.316	1835	319	0.012	0.034
Total		14694097	952023	100.000	100.000

<Chromatogram>

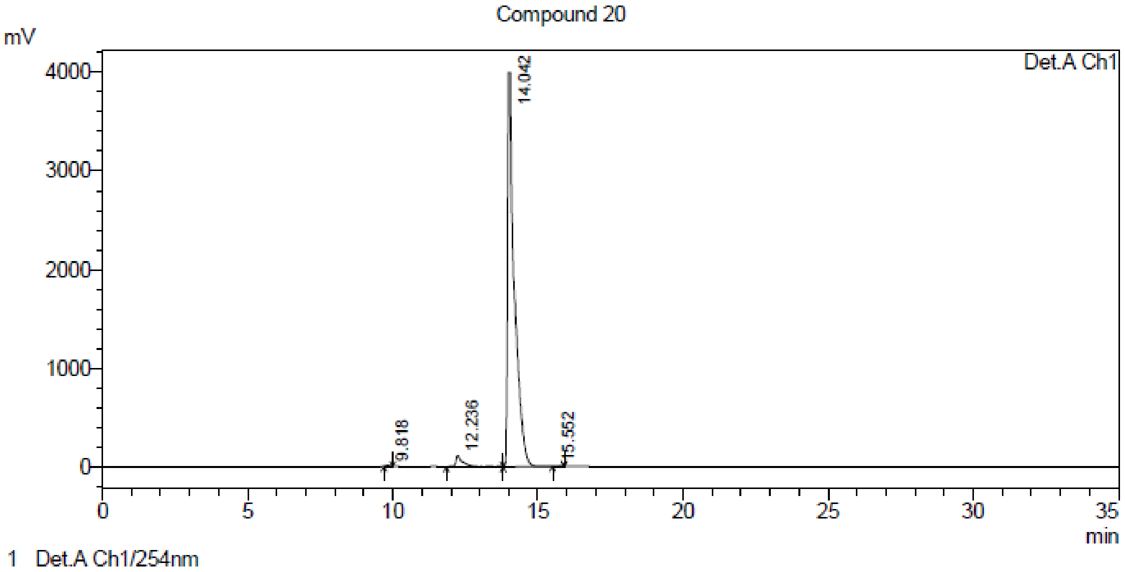

PeakTable

Detector A Ch1 254nm

Peak#	Ret. Time	Area	Height	Area %	Height %
1	9.818	95307	10692	0.142	0.259
2	12.236	1800851	113169	2.690	2.745
3	14.042	65011641	3994499	97.099	96.906
4	15.552	45882	3695	0.069	0.090
Total		66953681	4122055	100.000	100.000

<Chromatogram>

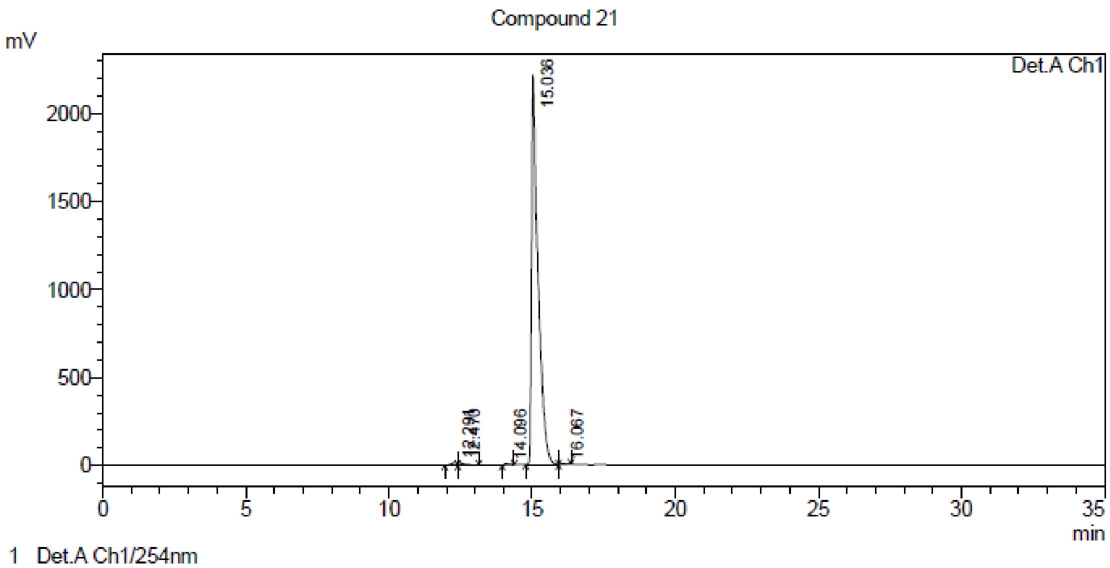

PeakTable

Detector A Ch1 254nm

Peak#	Ret. Time	Area	Height	Area %	Height %
1	12.291	195048	15975	0.551	0.709
2	12.470	194476	12263	0.550	0.544
3	14.096	93342	8418	0.264	0.373
4	15.036	34839914	2213179	98.480	98.178
5	16.067	55010	4409	0.155	0.196
Total		35377790	2254244	100.000	100.000

<Chromatogram>

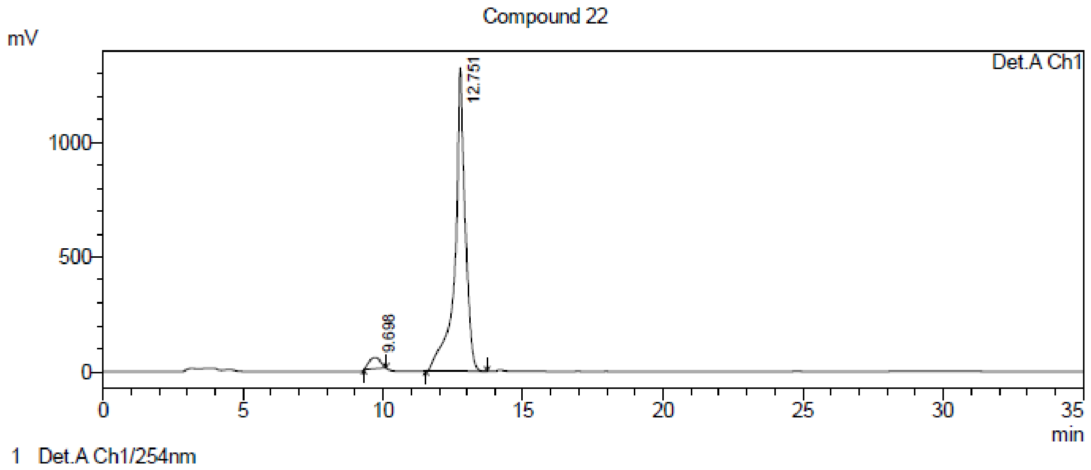

PeakTable

Detector A Ch1 254nm

Peak#	Ret. Time	Area	Height	Area %	Height %
1	9.698	1380392	49054	3.938	3.580
2	12.751	33673988	1321039	96.062	96.420
Total		35054380	1370093	100.000	100.000

<Chromatogram>

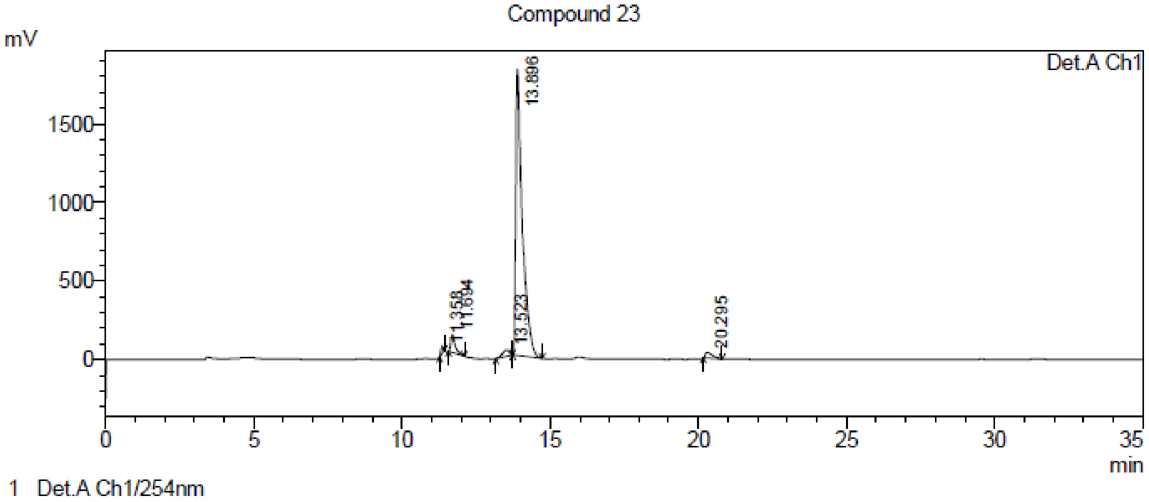

PeakTable

Detector A Ch1 254nm

Peak#	Ret. Time	Area	Height	Area %	Height %
1	11.358	209009	33515	0.699	1.636
2	11.694	1164302	114837	3.893	5.606
3	13.523	670449	41725	2.242	2.037
4	13.896	27243131	1821041	91.092	88.902
5	20.295	620324	37249	2.074	1.818
Total		29907214	2048368	100.000	100.000

<Chromatogram>

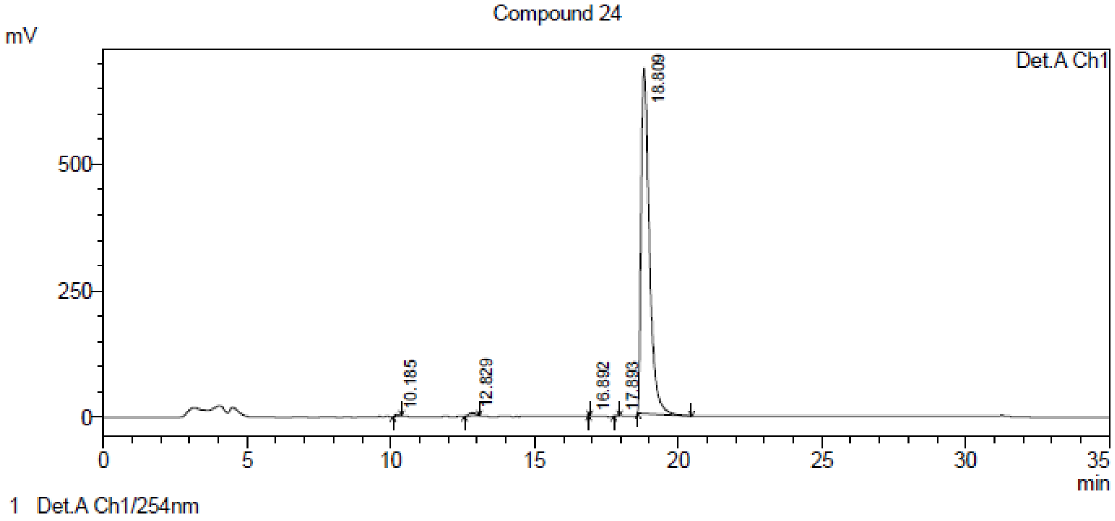

PeakTable

Detector A Ch1 254nm

Peak#	Ret. Time	Area	Height	Area %	Height %
1	10.185	31891	3962	0.233	0.573
2	12.829	84724	5328	0.618	0.770
3	16.892	59	30	0.000	0.004
4	17.893	5661	586	0.041	0.085
5	18.809	13579333	681993	99.107	98.568
Total		13701669	691898	100.000	100.000

<Chromatogram>

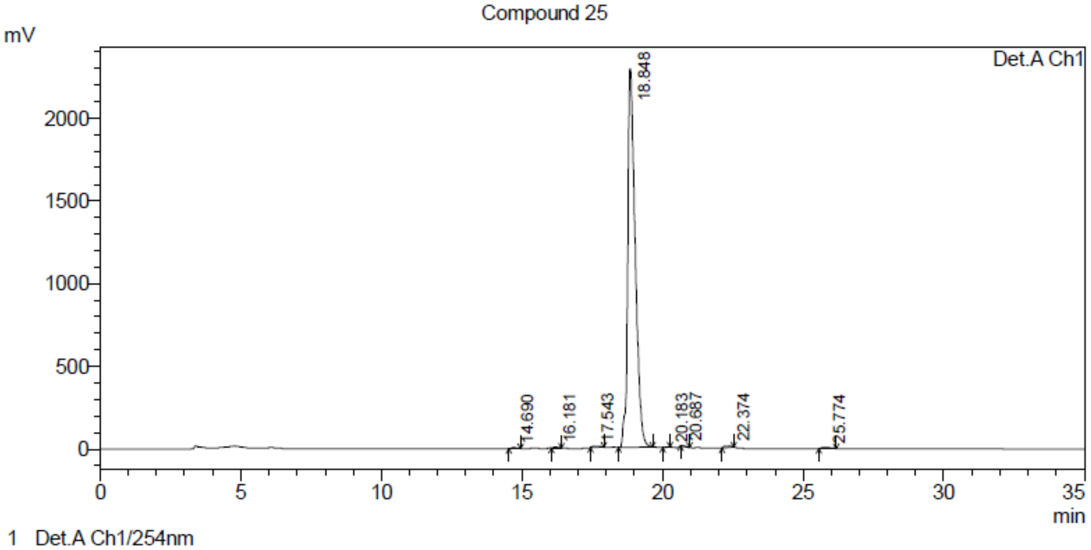

PeakTable

Detector A Ch1 254nm

Peak#	Ret. Time	Area	Height	Area %	Height %
1	14.690	69098	6226	0.161	0.267
2	16.181	51731	4983	0.121	0.214
3	17.543	162697	9197	0.379	0.394
4	18.848	42253857	2288815	98.491	98.177
5	20.183	19922	2370	0.046	0.102
6	20.687	31817	2959	0.074	0.127
7	22.374	169706	9144	0.396	0.392
8	25.774	142493	7623	0.332	0.327
Total		42901321	2331316	100.000	100.000

<Chromatogram>

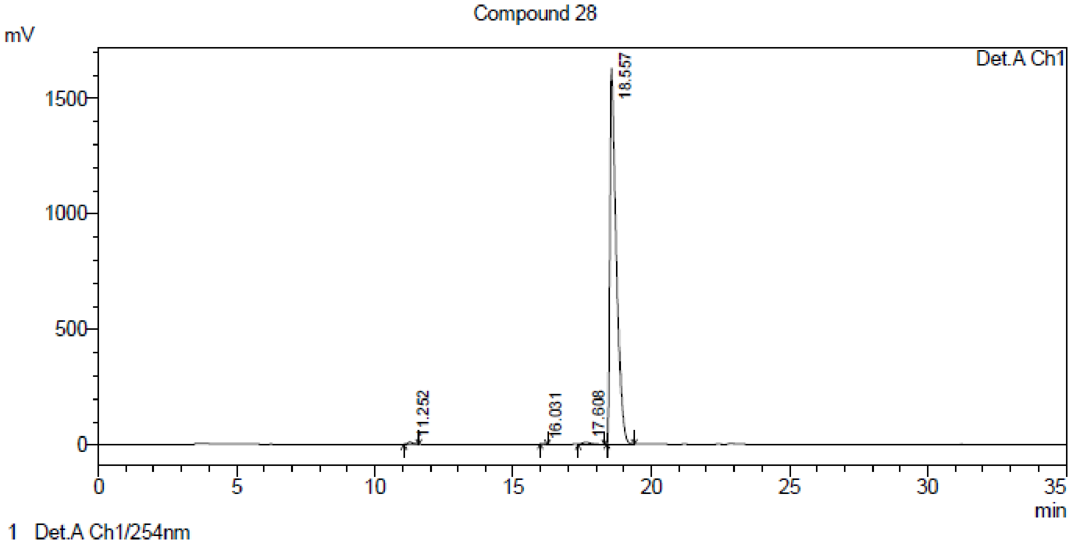

PeakTable

Detector A Ch1 254nm

Peak#	Ret. Time	Area	Height	Area %	Height %
1	11.252	129460	11486	0.478	0.697
2	16.031	2712	332	0.010	0.020
3	17.608	209993	10193	0.776	0.619
4	18.557	26725639	1625948	98.736	98.664
Total		27067805	1647959	100.000	100.000

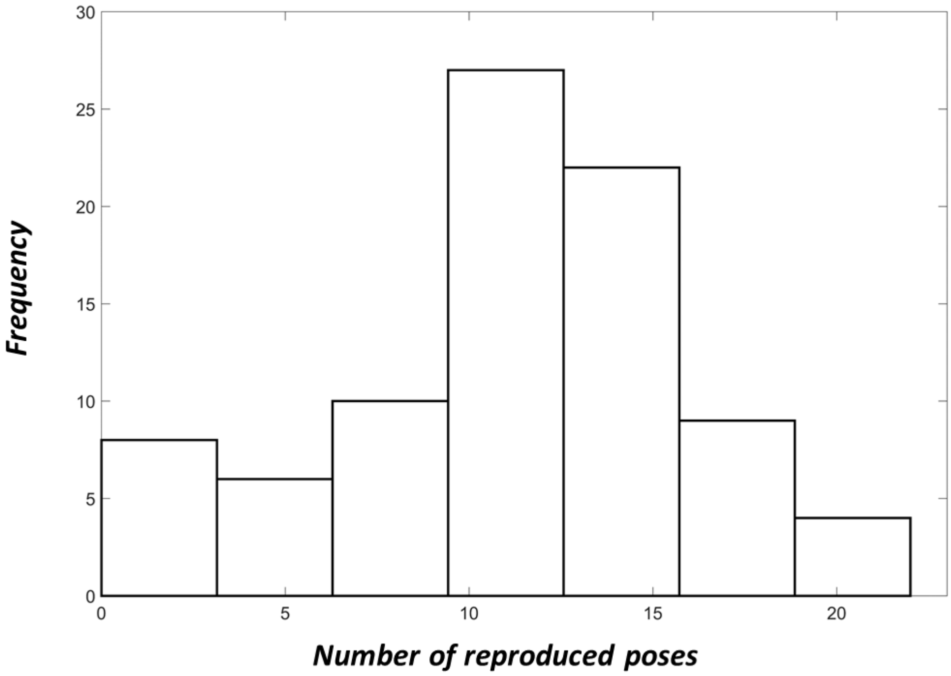

Figure S1. Pose reproducibility in docking simulation. The 45 crystal inhibitors were docked into the RCSB-deposited 86 B-Raf coordinates using Glide-SP. The poses reproduced within less than 2 Å rmsd compared to the crystal ligands were counted as a success. The 1UWJ-A coordinate that succeeded in 22 cases was chosen as a template for docking.

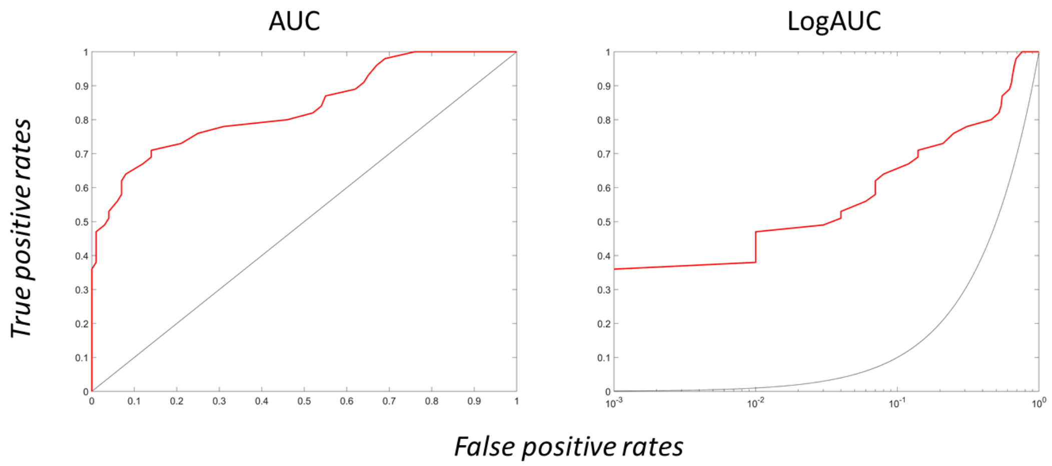

Figure S2. Enrichments of true positives in docking simulation. Docking simulation of crystal ligands and their property-matched but topologically different decoys were done with the coordinate of 1UWJ-A. The red and black lines indicate the enrichments in the current study and random selection, respectively. The values are 0.84 for AUC and 0.42 for LogAUC. The value of LogAUC corresponds to the area between the red and black lines.

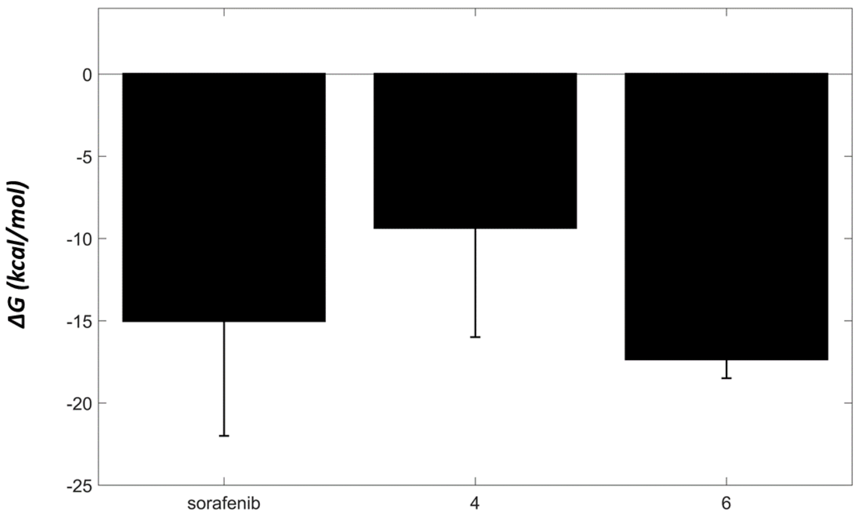

Figure S3. MMPBSA analyses of sorafenib, compounds 4 and 6. The mean (± standard deviation) values of MMPBSA-derived free energies for sorafenib, 4, and 6 in three independent runs with different random seeds were −15.0 (± 7.0), −9.3 (± 6.7), and −17.3 (± 1.2) kcal/mol, respectively.
